# Supplementary material for: Spatiotemporal brain hierarchies of auditory memory recognition and predictive coding
Source: Nat Commun. 2024 May 21;15:4313. doi: 10.1038/s41467-024-48302-4 (PMC11109219; doi:10.1038/s41467-024-48302-4)
Supplement: Supplementary file 1 — Supplementary information [file 41467_2024_48302_MOESM1_ESM.pdf]

Supplementary Information for

Spatiotemporal brain hierarchies of auditory memory recognition and predictive coding

Bonetti, L.[1,2,3,4], Fernández-Rubio, G.[1], Carlomagno, F.[1,5], Dietz, M. J.[6], Pantazis, D.[7], Vuust, P.[1], Kringelbach, M. L.[1,2,3]*

[1] Center for Music in the Brain, Department of Clinical Medicine, Aarhus University & The Royal Academy of Music, Aarhus/Aalborg, Denmark

[2] Centre for Eudaimonia and Human Flourishing, Linacre College, University of Oxford, Oxford, United Kingdom

[3] Department of Psychiatry, University of Oxford, Oxford, United Kingdom

[4] Department of Psychology, University of Bologna, Italy

[5] Department of Education, Psychology, Communication, University of Bari Aldo Moro, Italy

[6] Center of Functionally Integrative Neuroscience, Institute of Clinical Medicine, Aarhus University, Denmark

[7] McGovern Institute for Brain Research, Massachusetts Institute of Technology (MIT), MA 02139, Cambridge, USA

**Corresponding author: leonardo.bonetti@psych.ox.ac.uk*

The supplementary information related to this study is organised as Supplementary Methods *(i)* and Supplementary Figures (*ii*).

The Supplementary Figures are available in high definition at the following link: https://doi.org/10.5281/zenodo.10715160

SUPPLEMENTARY METHODS

Functional parcellation

After calculating the contrasts in magnetoencephalography (MEG) source space (see **Results**: *Univariate analysis on the MEG channels* in the main manuscript), our primary procedure involved analysing the time series obtained for the automated anatomical labelling (AAL) regions of interest (ROIs). In addition to this main approach, to enhance the robustness of our results, we conducted additional, parallel analyses using a functional parcellation derived from the data. The following section outlines the steps taken to create this functional parcellation:

1. For each contrast of the brain voxels time series computed between experimental conditions (see **Table 2**), we selected the significant brain voxels which showed greater t-values than the averaged t-value plus one standard deviation. This returned a binary representation of the 3559 brain voxels where "1" indicated the voxels which showed the strongest differences in the previous contrasts, while "0" otherwise.
2. We computed a series of k-means clusters (clustering solutions from k = 1 to k = 20) on the Montreal Neurological Institute (MNI) spatial coordinates of the selected voxels (the ones indicated by "1" in the binary brain). The best clustering solution was established using the silhouette coefficient, which is a value (ranging from −1 to +1) showing the similarity of an element with its own cluster (cohesion) when compared to other clusters (separation). A high silhouette coefficient value indicates that the element is well matched to its own cluster and poorly to the neighbouring clusters. This procedure allowed us to objectively determine what was the best clustering configuration of the brain voxels that showed the biggest differences between experimental conditions, as well as how the voxels were divided into distinct clusters.

The results of this clustering procedure showed that the best clustering configuration objectively involved four clusters (k = 4). As shown in **Figure S14**, the four clusters roughly corresponded to bilateral medial cingulate gyrus (MC), right hippocampal area and inferior temporal cortex (HITR), left auditory cortex (ACL), and bilateral ventromedial prefrontal cortex (VMPFC).

Finally, we wished to refine the spatial extent of those very broad ROIs. To this aim, for each of the brain voxels and time-point of the four broad ROIs, we observed the following steps:

1. We computed t-values contrasting memorised (M) versus any category of novel (N; i.e. M versus NT1, M versus NT2, M versus NT3, M versus NT4).

2. We isolated the strongest t-value in absolute terms. This allowed us to identify the peaks of differential activity occurring between M and N for each broad ROI.
3. We used those peaks (averaged in a time window of ±20 ms) and strict *t-value* thresholds (abs(t) > 3) to isolate the brain voxels that, for each ROI, mainly contributed to discriminate M versus N. This procedure refined the spatial extent of the four broad regions of interests that we previously identified (HITR, MC, VMPFC, ACL).
4. Finally, to cover potential brain hemispheric differences, we created two more ROIs which mirrored HITR and ACL in the opposite hemisphere (HITL and ACR, respectively). (There was no need to do that for MC and VMPFC since they were identified already across the two hemispheres). The final functional ROIs are depicted in **Figure S10**.

Statistical analysis on functional ROIs time series

After defining the functional ROIs, we computed one t-test for each time-point and each combination of M versus Ns. Finally, we corrected for multiple comparisons using one-dimensional (1D) cluster-based Monte-Carlo simulations (MCS, $\alpha = .05$, MCS *p-value* = .001). Confirming the results obtained using the AAL ROIs shown in **Figure 4**, this analysis returned several significant clusters of differential brain activity over time between M and Ns. As shown in **Figure S11**, M versus N was characterized by stronger activity in VMPFC, ACL, and HITR after 350 – 450 ms from the onset of each tone. Similarly, M presented stronger negative activity ($p < .001$) than N in the MC after 400 – 500 ms from the onset of each tone.

Contrast	ROI	Temporal extent of the largest clusters from the 1st tone of the sequence	Peak t-value	P-value
Positive activity				
M versus NT1 (onset deviation NT1: 350 ms)	VMPFC	900 – 1190	6.13	< .001
	ACL	900 – 1040	6.26	< .001
	HITR	1660 – 1930	5.45	< .001
M versus NT2 (onset deviation NT2: 700 ms)	VMPFC	1680 – 1930	5.94	< .001
	ACL	1260 – 1390	3.56	< .001
	HITR	1640 – 1930	6.12	< .001
M versus NT3 (onset deviation NT3: 1005 ms)	VMPFC	1310 – 1540	7.04	< .001
	ACL	1250 – 1390	4.80	< .001
	HITR	1310 – 1530	6.69	< .001
M versus NT4 (onset deviation NT4: 1400 ms)	VMPFC	1680 – 1910	8.92	< .001
	ACL	1640 – 1790	6.93	< .001
	HITR	1680 – 1900	7.45	< .001
Negative activity				
M versus NT1	MC	680 – 860	-5.18	< .001
M versus NT2	MC	980 – 1180	-5.77	< .001
M versus NT3	MC	1360 – 1550	-5.41	< .001
M versus NT4	MC	1730 – 1920	-5.40	< .001

Table S1. Largest clusters of stronger activity of memorised (M) versus novel sequences (Ns).

Largest clusters of significantly stronger activity of M versus Ns (two-sided t-tests were computed independently for each time-point and ROI, and Monte-Carlo simulations (MCS) employed to correct for multiple comparisons) computed for the six regions of interest (ROIs) considered in the study. The table shows the contrast, the correspondent ROI, the temporal extent (in ms) of the largest cluster, the peak t-value of the cluster and the associated Monte-Carlo simulations (MCS) p-value. The MC shows stronger negativity since the polarity of the MC signal was negative. All clusters are reported in detail in ***Supplementary Data 9****.*

Conversely, late N100 responses localised in AC were stronger for N versus M. For instance, the temporal extent of the largest cluster of ACL for M versus NT1 was 520 – 700 ms after the onset of the first tone of the sequence (peak *t-value* = 6.75, $p < .001$). Moreover, HIT and VMPFC showed a stronger response for N versus M occurring at about 250 – 300 ms after

altering the original memorised sequences. For instance, the temporal extent of the largest cluster of HITR for M versus NT4 was 1680 – 1900 ms after the onset of the first tone of the sequence (peak *t-value* = 7.45, $p < .001$); the temporal extent of the largest cluster of VMPFC for M versus NT4 was 1680 – 1910 ms after the onset of the first tone of the sequence (peak *t-value* = 8.92, $p < .001$). **Supplementary Data 13** reports the main significant clusters, while complete statistical results are reported in **Supplementary Data 9**.

Since source reconstruction can produce leakage of the brain signals between the ROIs [1], we took additional measures to ensure the robustness of our results. Specifically, we applied the multivariate source leakage correction method proposed by Colclough and colleagues [1] to the ROIs time series. The outcomes of this analysis, as illustrated in **Figure S15** and described in **Supplementary Data 13**, align with our initial findings, corroborating the robustness of our results.

Dynamic causal modelling – functional ROIs

Dynamic causal modelling (DCM) aims to infer the causal structure of interconnected or dispersed dynamical systems such as a network of brain regions. It employs a Bayesian model comparison approach that involves evaluating competing models which explain the generation of time series data associated with alternative network architectures and hierarchical relationships between ROIs. In this study, DCM was used to test our hypothesised model of brain hierarchies during recognition of memorised and novel musical sequences against five competing models, which are described in detail in the Methods section and illustrated in **Figure 1g**. These six models comprised the six AAL ROIs described in the previous section (ACL, ACR, HITL, HITR, VMPFC, MCC), and depicted in **Figure S8**. Our hypothesised model consisted of feedforward connections from ACL, ACR to HITL, HITR, VMPFC, MC and feedback connections from HITL, HITR, VMPFC, MCC to ACL, ACR (**Figure 6a**, top left). We computed a series of DCM analyses independently for each tone of the sequences, in a 350 ms time-window after each tone onset. Specifically, we computed an independent DCM for tones two, three, four and five for condition M. In relation to the N conditions, we were interested only in the tone that introduced the variation in the sequences. Thus, we computed four DCMs, organised as follows: tone two for NT1; tone three for NT2; tone four for NT3; tone five for NT4. To compare alternative dynamic causal models, we estimated the free energy F associated with the computation of each model (model evidence). To study the consistency of F across the population, we used a random-effects Bayesian model selection (RFX BMS)

procedure, which provides the posterior probability and protected exceedance probability of each model, indicating which of the alternative models has the strongest model evidence within the whole population. In addition, we computed the Bayesian omnibus risk (BOR), which represents the probability that the protected exceedance probability associated with each model comparison is attributable to chance within the sample of participants (**Figure S16**). This procedure was observed independently for the eight DCM analyses described above (four for M and four for Ns).

Figure S16 shows posterior probability, protected exceedance probability and BOR for each model in each of the eight DCM analyses that we computed. The results obtained from the analysis of functional ROIs largely corroborated the findings derived from the AAL ROIs. Specifically, they consistently supported our hypothesised model as the one with the strongest evidence across all N conditions and for tones one and four in condition M. In contrast, tones three and five revealed a similar model with the medial cingulate at the top of the hierarchy (model 6) as having the strongest evidence for condition M. These subtle differences may be attributed to the fact that the functional ROIs encompassed larger brain regions compared to the AAL ROIs, resulting in overlapping but not entirely identical results. As follows, we report the BOR values for each of the eight DCM analyses: $BOR_{M\text{-}tone2}$ = 5.49e-08; $BOR_{M\text{-}tone3}$ = .028; $BOR_{M\text{-}tone4}$ = .010; $BOR_{M\text{-}tone5}$ = 1.43e-04; $BOR_{NT1\text{-}tone2}$ = 9.33e-09; $BOR_{NT2\text{-}tone3}$ = .004; $BOR_{NT3\text{-}tone4}$ = 7.11e-05; $BOR_{NT4\text{-}tone5}$ = 2.07e-04.

Time-frequency analysis – functional ROIs

We computed the power spectra of the six ROIs of the functional parcellation using complex Morlet wavelet transform (from 1 to 60 Hz with 1-Hz intervals) [2]. As done for the AAL ROIs, this analysis was conducted for induced responses, independently for the six functional ROIs and for the four contrasts considered in this study (i.e., M versus NT1, M versus NT2, M versus NT3, M versus NT4). The *p*-values emerging from the contrasts (two-sided t-tests, independently conducted for each frequency and time-point) were binarized (α = .05) and then submitted to a 2D MCS (MCS *p*-value = .001). **Figures S17** and **S18** and **Supplementary Data 14** show that the outcome of this procedure closely aligned with the results obtained for the AAL ROIs.

SUPPLEMENTARY FIGURES

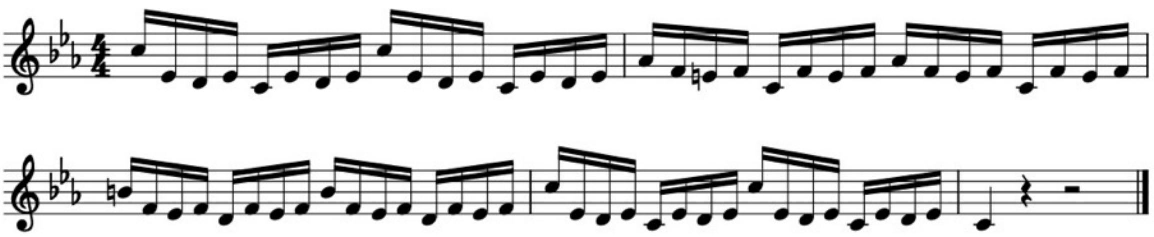

Figure S1. Musical piece used in the encoding phase of the auditory old/new paradigm

*In the first part of the auditory old/new paradigm, first participants listened to a short musical piece twice and were asked to memorise it as much as possible. The musical piece consisted of the first four bars of the right-hand part of Johann Sebastian Bach's Prelude No. 2 in C Minor, BWV 847. In this piece, each bar comprised 16 tones. Thus, the total number of tones was 16*4 = 64. Each tone lasted approximately 350 ms for a total duration of 22400 ms. In addition, to provide a sense of musical closure, we included a final tone after the four bars which lasted 1000 ms. Thus, the total was 23400 ms which correspond to 23.4 seconds. This figure illustrates the piece in musical notation.*

Melody 1

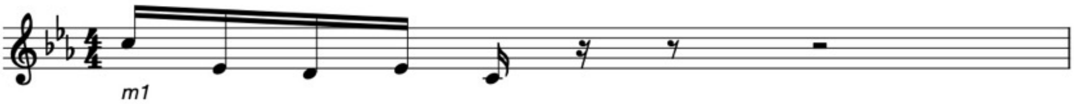

m1

Inverted melodic contour I

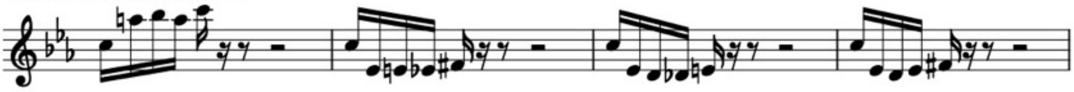

m1t1e1 *m1t2e1* *m1t3e1* *m1t4e1*

Same tone scrambled I

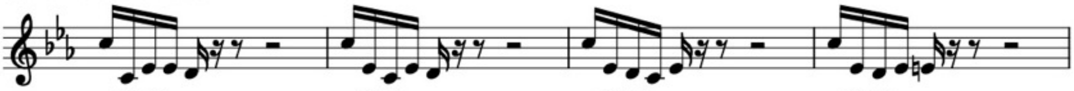

m1t1e2 *m1t2e2* *m1t3e2* *m1t4e2*

Same tone scrambled II

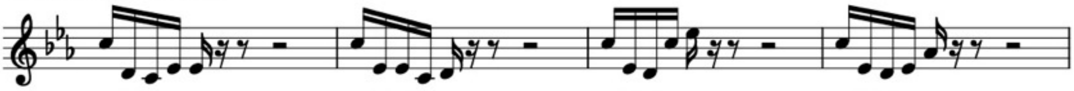

m1t1e3 *m1t2e3* *m1t3e3* *m1t4e3*

Same tone I

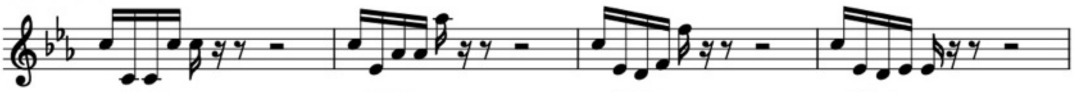

m1t1e4 *m1t2e4* *m1t3e4* *m1t4e4*

Same tone II

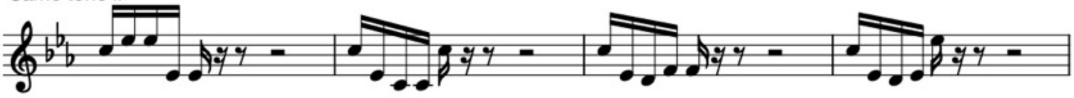

m1t1e5 *m1t2e5* *m1t3e5* *m1t4e5*

Scrambling intervals

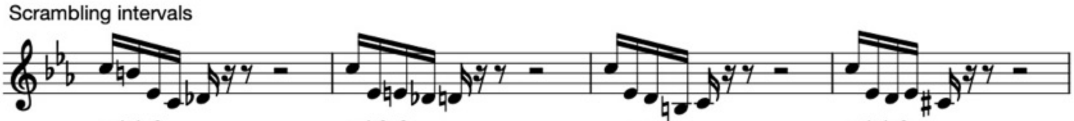

m1t1e6 *m1t2e6* *m1t3e6* *m1t4e6*

Inverted melodic contour II

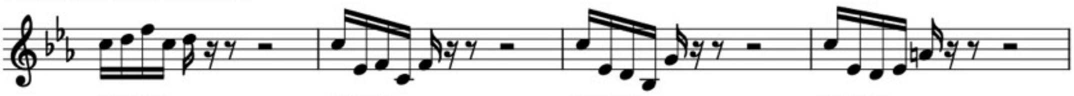

m1t1e7 *m1t2e7* *m1t3e7* *m1t4e7*

Same tone scrambled III

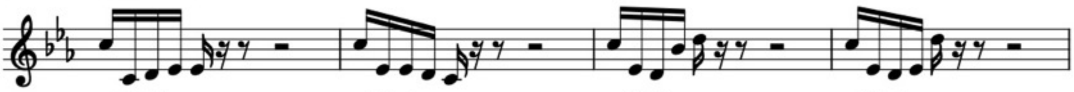

m1t1e8 *m1t2e8* *m1t3e8* *m1t4e8*

Same tone III

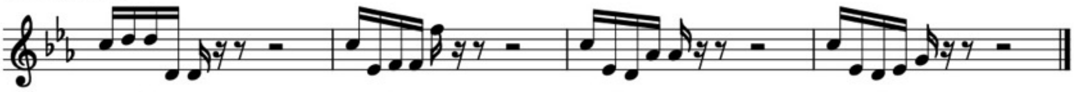

m1t1e9 *m1t2e9* *m1t3e9* *m1t4e9*

Melody 2

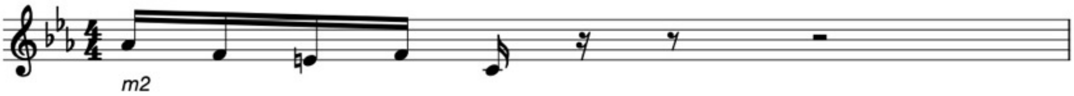

Inverted melodic contour I

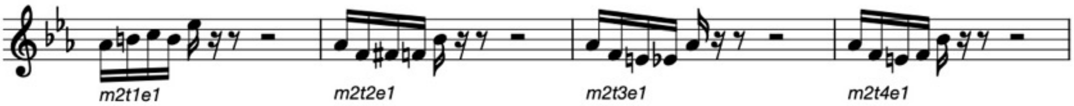

Same tone scrambled I

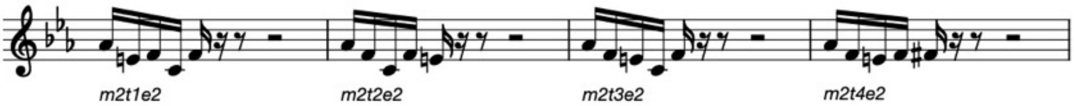

Same tone scrambled II

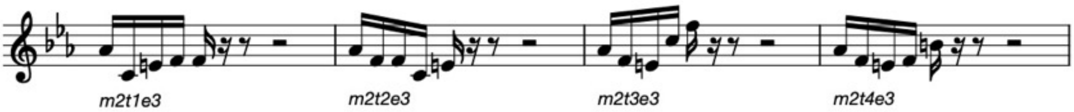

Same tone I

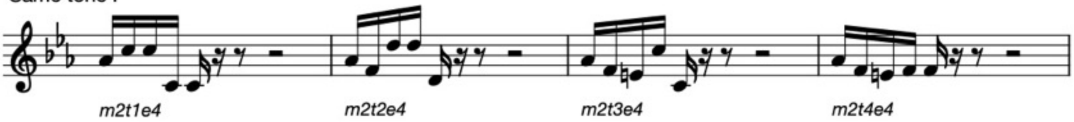

Same tone II

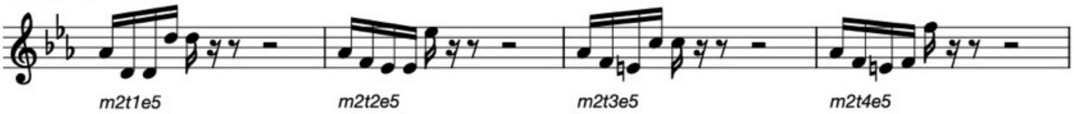

Scrambling intervals

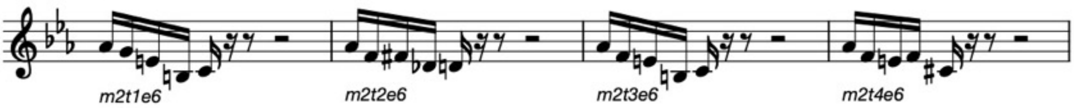

Inverted melodic contour II

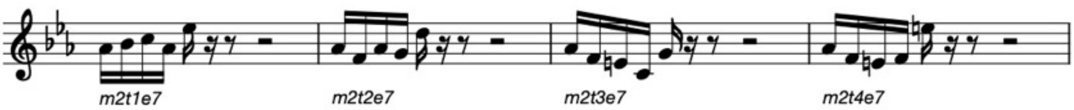

Same tone scrambled III

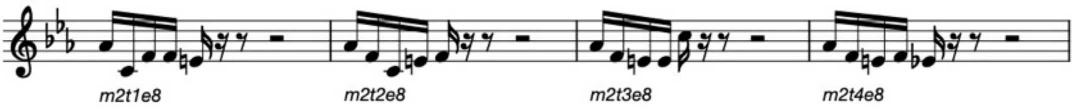

Same tone III

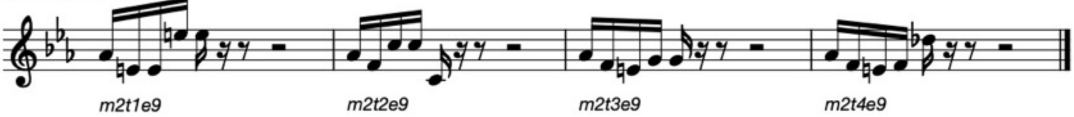

Melody 3

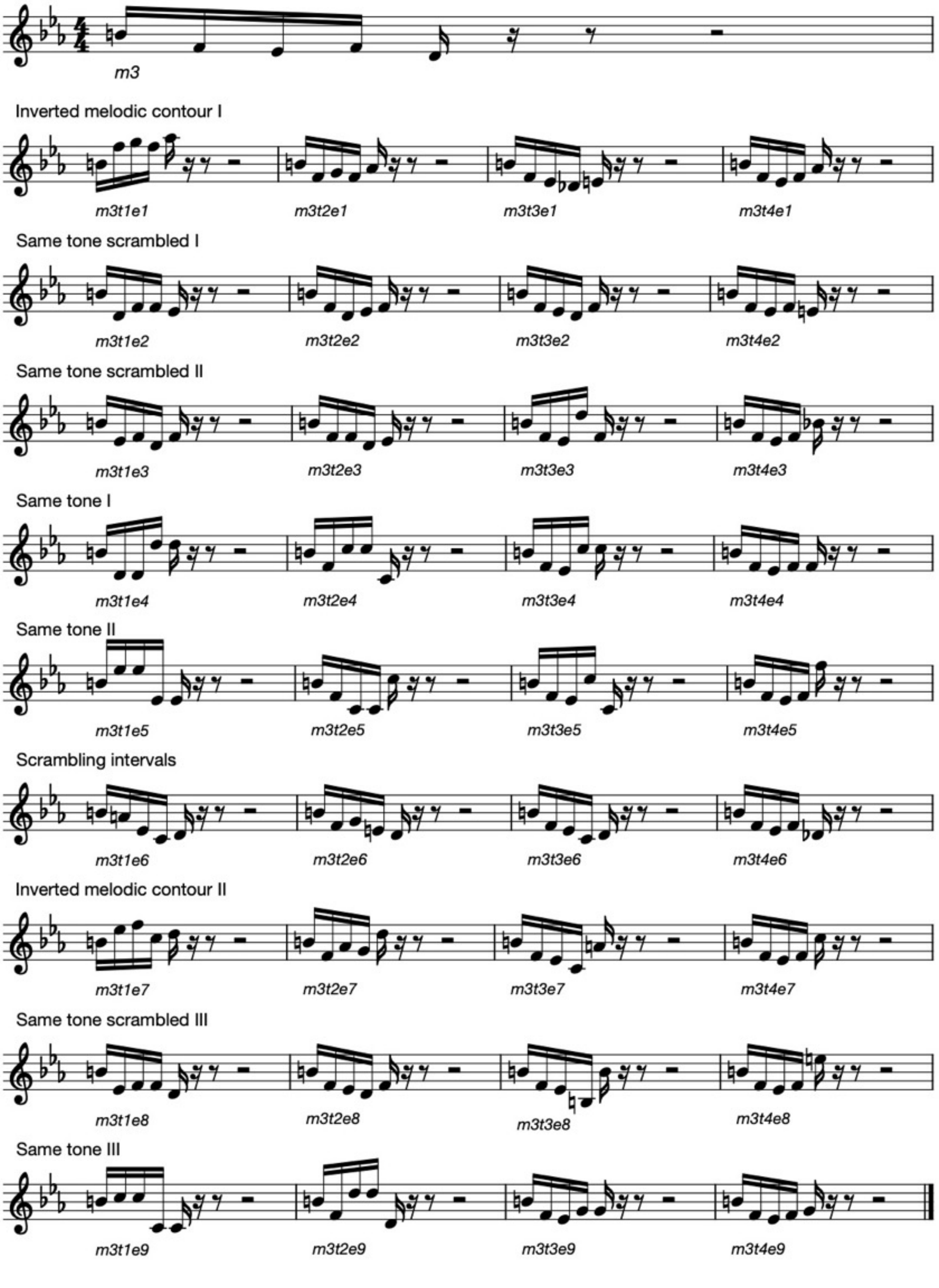
m3
Inverted melodic contour I
m3t1e1
m3t2e1
m3t3e1
m3t4e1
Same tone scrambled I
m3t1e2
m3t2e2
m3t3e2
m3t4e2
Same tone scrambled II
m3t1e3
m3t2e3
m3t3e3
m3t4e3
Same tone I
m3t1e4
m3t2e4
m3t3e4
m3t4e4
Same tone II
m3t1e5
m3t2e5
m3t3e5
m3t4e5
Scrambling intervals
m3t1e6
m3t2e6
m3t3e6
m3t4e6
Inverted melodic contour II
m3t1e7
m3t2e7
m3t3e7
m3t4e7
Same tone scrambled III
m3t1e8
m3t2e8
m3t3e8
m3t4e8
Same tone III
m3t1e9
m3t2e9
m3t3e9
m3t4e9

Figure S2. Auditory sequences used in the experiment

The figure shows all temporal sequences used in the experiment in musical notation. The memorised (M) sequences were three and comprised the first five tones of the first three measures of the musical piece. These three sequences (m1, m2, and m3) were presented nine times each, for a total of 27 trials. The novel (N) sequences were created through systematic variations of the three M sequences. This procedure consisted of changing every musical tone of the sequence after the first (NT1), second (NT2), third (NT3) or fourth (NT4) tone. We created nine variations for each of the original M sequences and each of the four categories of N. This resulted in 27 N sequences for each category, and 108 N in total. To be noted, as shown in this figure, the variations were created according to the following rules: (i) inverted melodic contours (used twice): the melodic contour of the variation was inverted with respect to the original M sequence (i.e., if the M sequence had the following melodic contour: down-down-up-down, the N sequence would be: up-up-down-up); (ii) same tone scrambled (used three times): the remaining tones of the M sequence were scrambled (e.g., M sequence: C-E-D-E-C, was converted into NT1 sequence: C-C-E-E-D), but in the case of NT4, where only the last tone is different from the M sequence, we substituted the last tone of the M sequence with a random tone; (iii) same tone (used three times): the same tone was repeatedly used, in some cases varying only the octave (e.g., M sequence: C-E-D-E-C, was transformed into NT1 sequence: C-E- E- E8- E8); (iv) scrambling intervals (used once): the intervals between the tones were scrambled (e.g., M sequence: 6thm – 2ndm – 2ndm – 3rdm, was adapted to NT1 sequence: 2ndm, 6thm, 3rdm, 2ndm). The harmonic structure of Ns with regards to Ms was preserved in most of the cases, with a few exceptions occurring for 'inverted melodic contours' and 'scrambling intervals' variations. This strategy was implemented to avoid potential confounding variables stemming from changes in harmony.

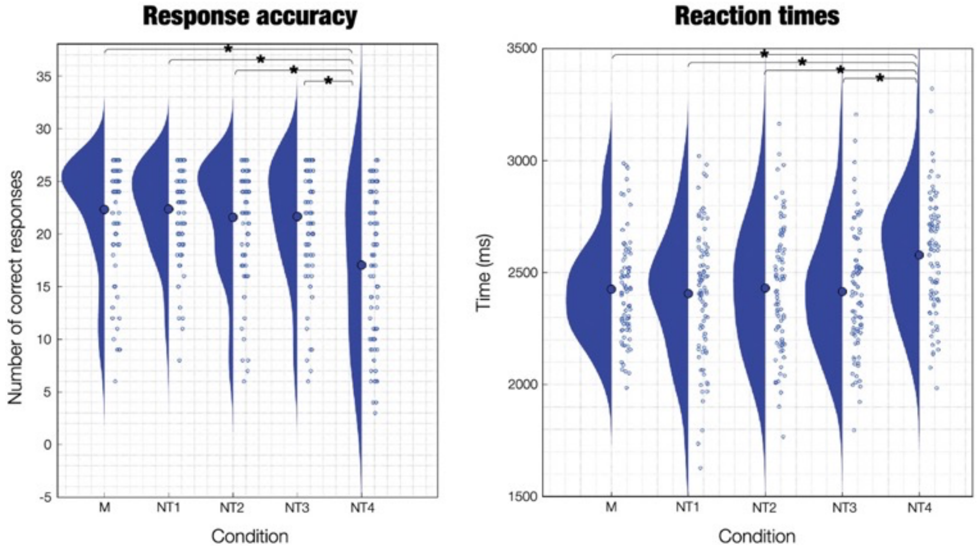

Figure S3. Behavioural performance in the auditory recognition task

Scatter and violin plots depicting the number of correct responses and reaction times for the experimental task. The plots illustrate each experimental condition separately. Each dot represents a participant (n = 83). The graphs and stars indicate that both accuracy and reaction times for novel T4 (NT4), tested with one-sided analysis of variance (ANOVA), were significantly different (p < .01) from memorised (M, accuracy: p = .001; reaction time: p = .0016), novel T1 (NT1, accuracy: p = .001; reaction time: p = .0013), novel T2 (NT2, accuracy: p = .0003; reaction time: p = .0054) and novel T3 (NT3, accuracy: p = .0001; reaction time: p = .0008).

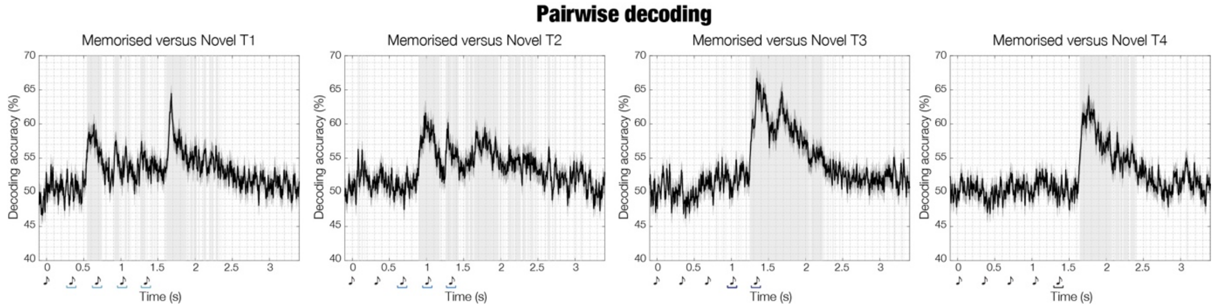

Figure S4. Pairwise decoding time series.

Multivariate pattern analysis decoding the different neural activity associated with memorised versus novel musical sequences. Each plot shows the decoding time series, averaged over participants (n = 83), for one of the four rounds of pairwise decoding that we computed (memorised [M] versus novel T1, M versus novel T2, M versus novel T3, M versus novel T4). Gray areas indicate the significant time-points returned by sign permutation test against chance level and one-dimensional Monte Carlo simulations (MCS; MCS, α = .05, MCS p-value = .001) applied to correct for multiple comparisons. Dot lines indicate standard errors. The sketch of the musical tones represents the onset of the sounds forming the temporal sequences and the graphs underneath the notes mark the number of tones changed.

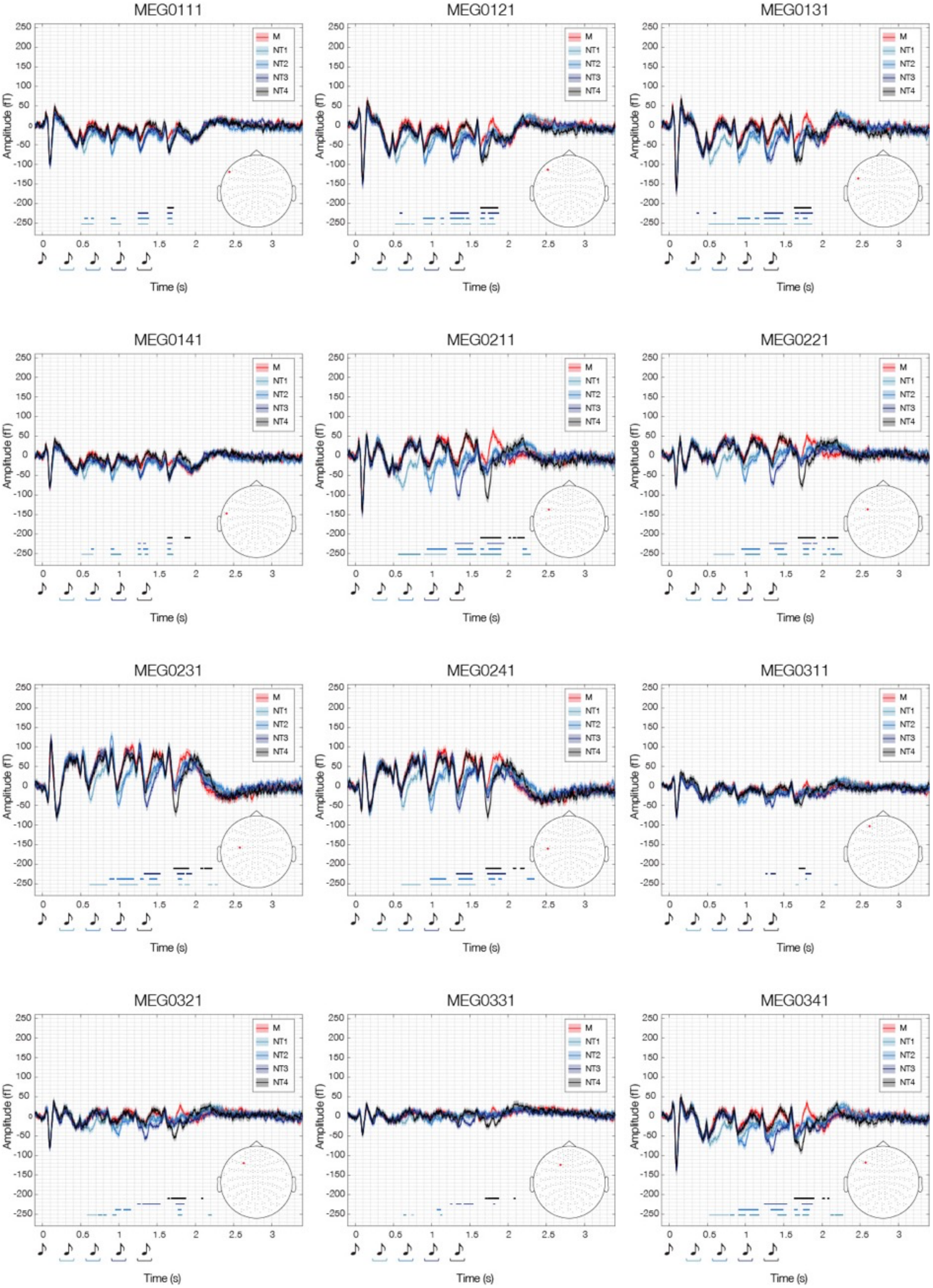
MEG0111
MEG0121
MEG0131
MEG0141
MEG0211
MEG0221
MEG0231
MEG0241
MEG0311
MEG0321
MEG0331
MEG0341
M
NT1
NT2
NT3
NT4
Amplitude (fT)
Time (s)

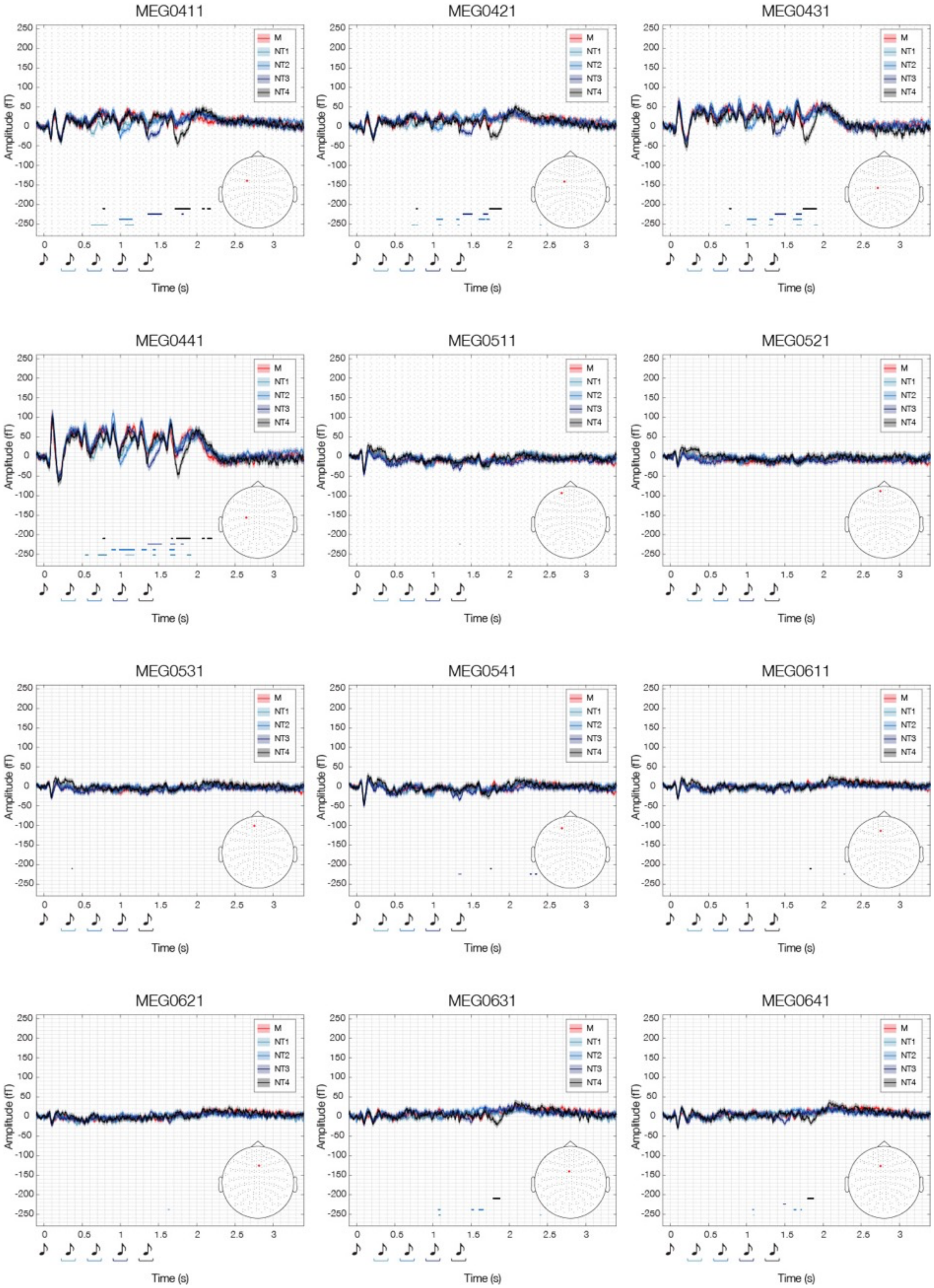
MEG0411
MEG0421
MEG0431
MEG0441
MEG0511
MEG0521
MEG0531
MEG0541
MEG0611
MEG0621
MEG0631
MEG0641
M
NT1
NT2
NT3
NT4
Amplitude (fT)
250
200
150
100
50
0
-50
-100
-150
-200
-250
0
0.5
1
1.5
2
2.5
3
Time (s)

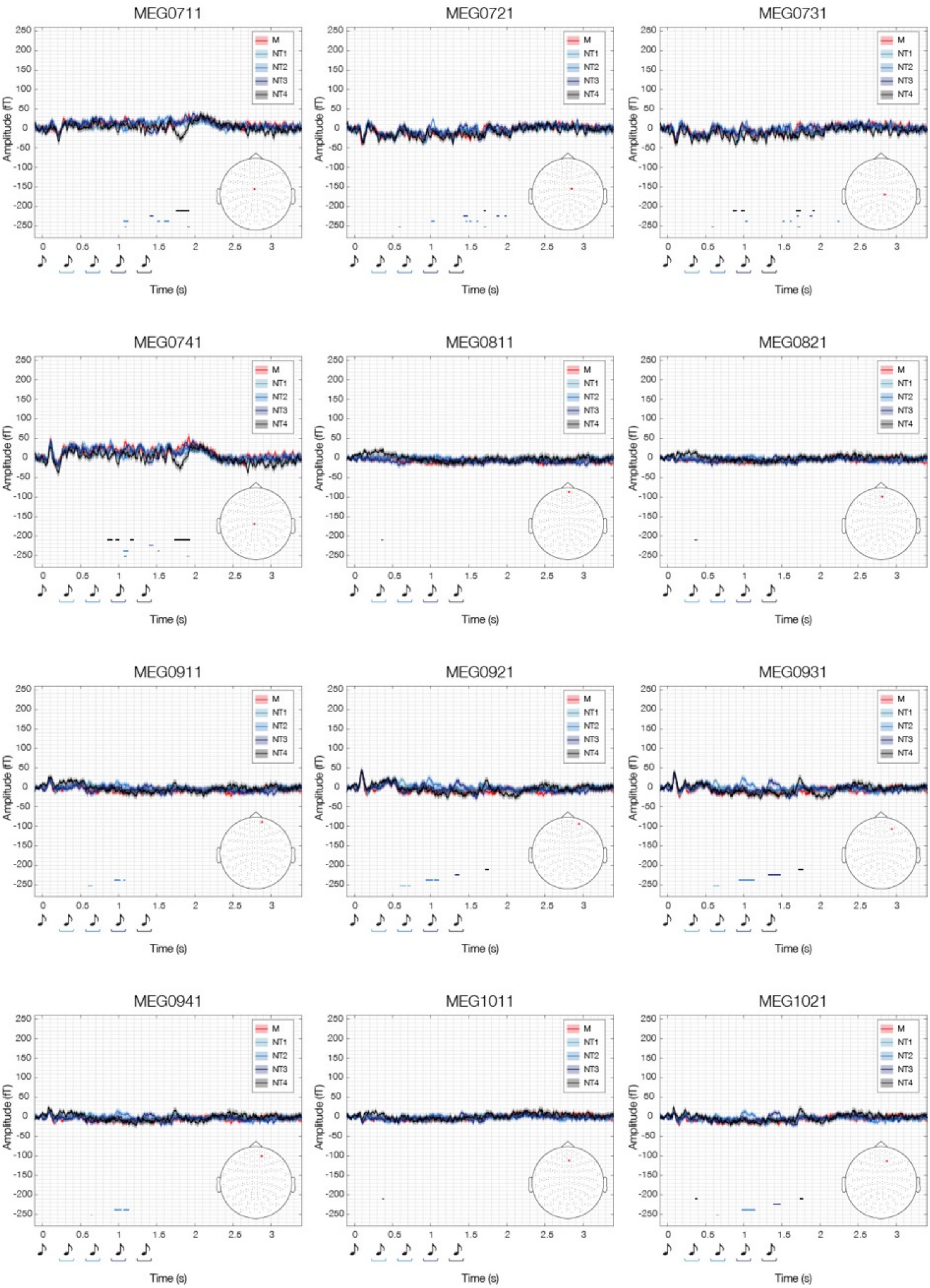
MEG0711
MEG0721
MEG0731
MEG0741
MEG0811
MEG0821
MEG0911
MEG0921
MEG0931
MEG0941
MEG1011
MEG1021
Amplitude (fT)
Time (s)
M
NT1
NT2
NT3
NT4

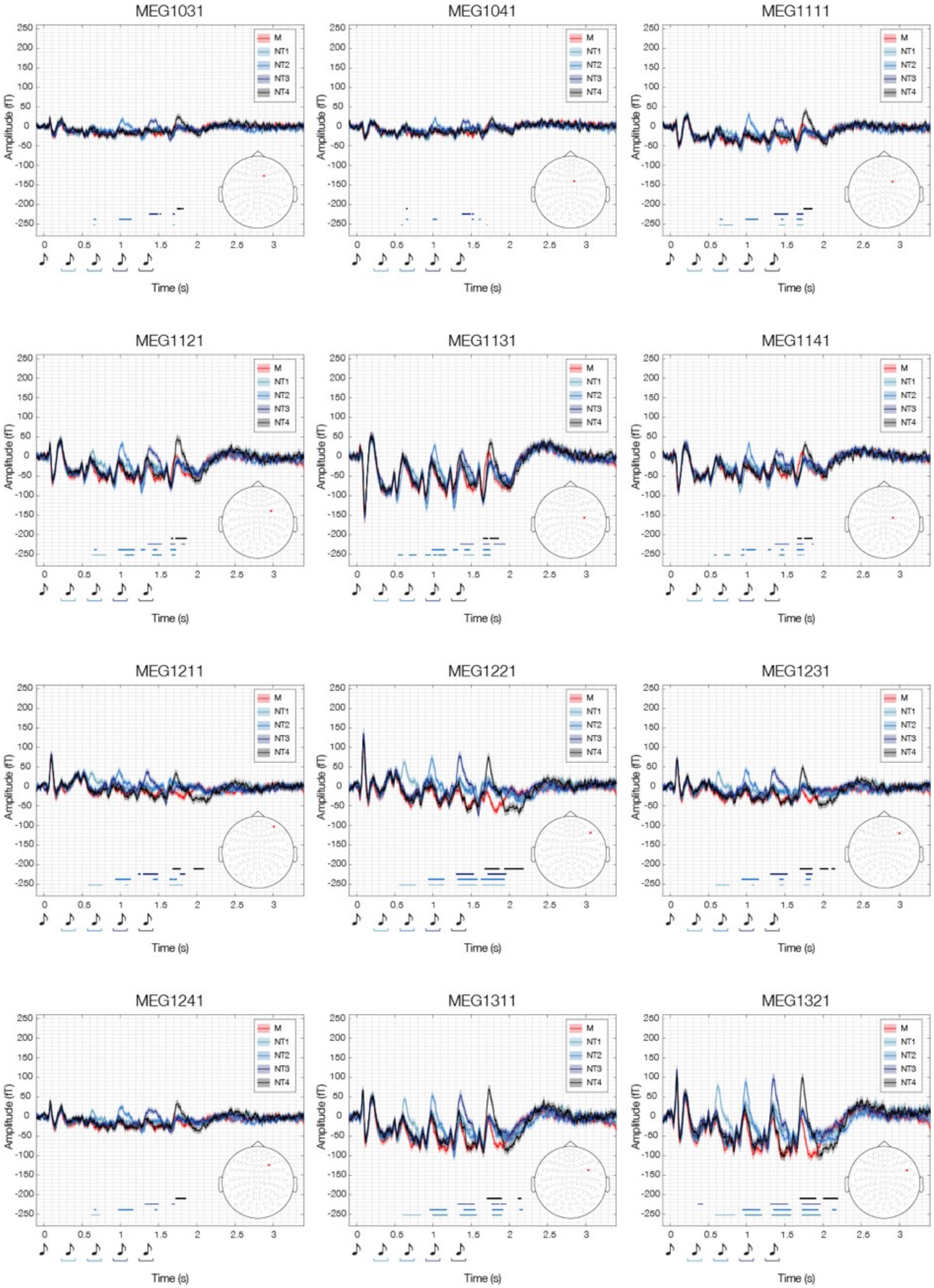
MEG1031
MEG1041
MEG1111
MEG1121
MEG1131
MEG1141
MEG1211
MEG1221
MEG1231
MEG1241
MEG1311
MEG1321
M
NT1
NT2
NT3
NT4
Amplitude (fT)
Time (s)

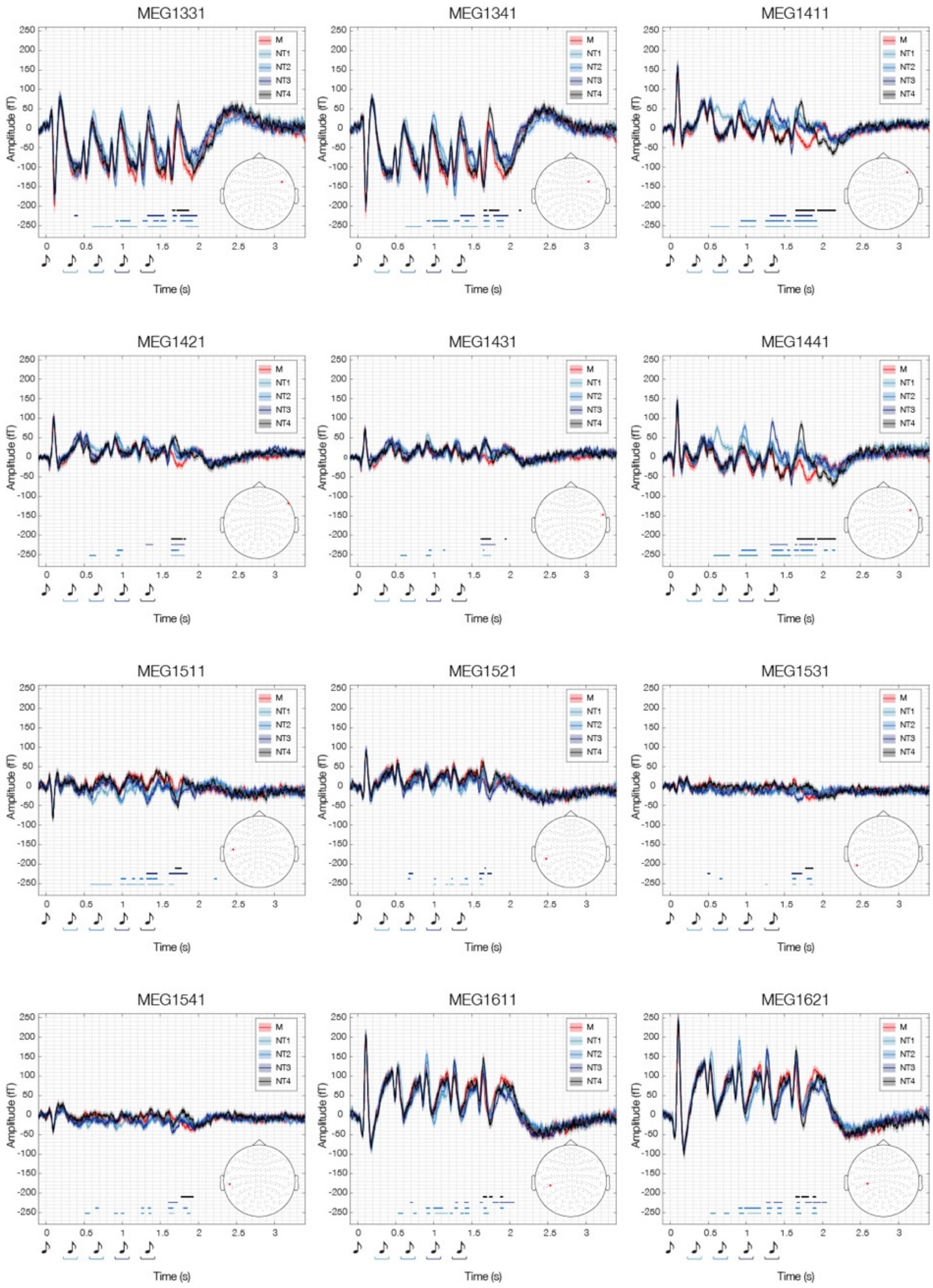
MEG1331
MEG1341
MEG1411
MEG1421
MEG1431
MEG1441
MEG1511
MEG1521
MEG1531
MEG1541
MEG1611
MEG1621
M
NT1
NT2
NT3
NT4
Amplitude (fT)
Time (s)

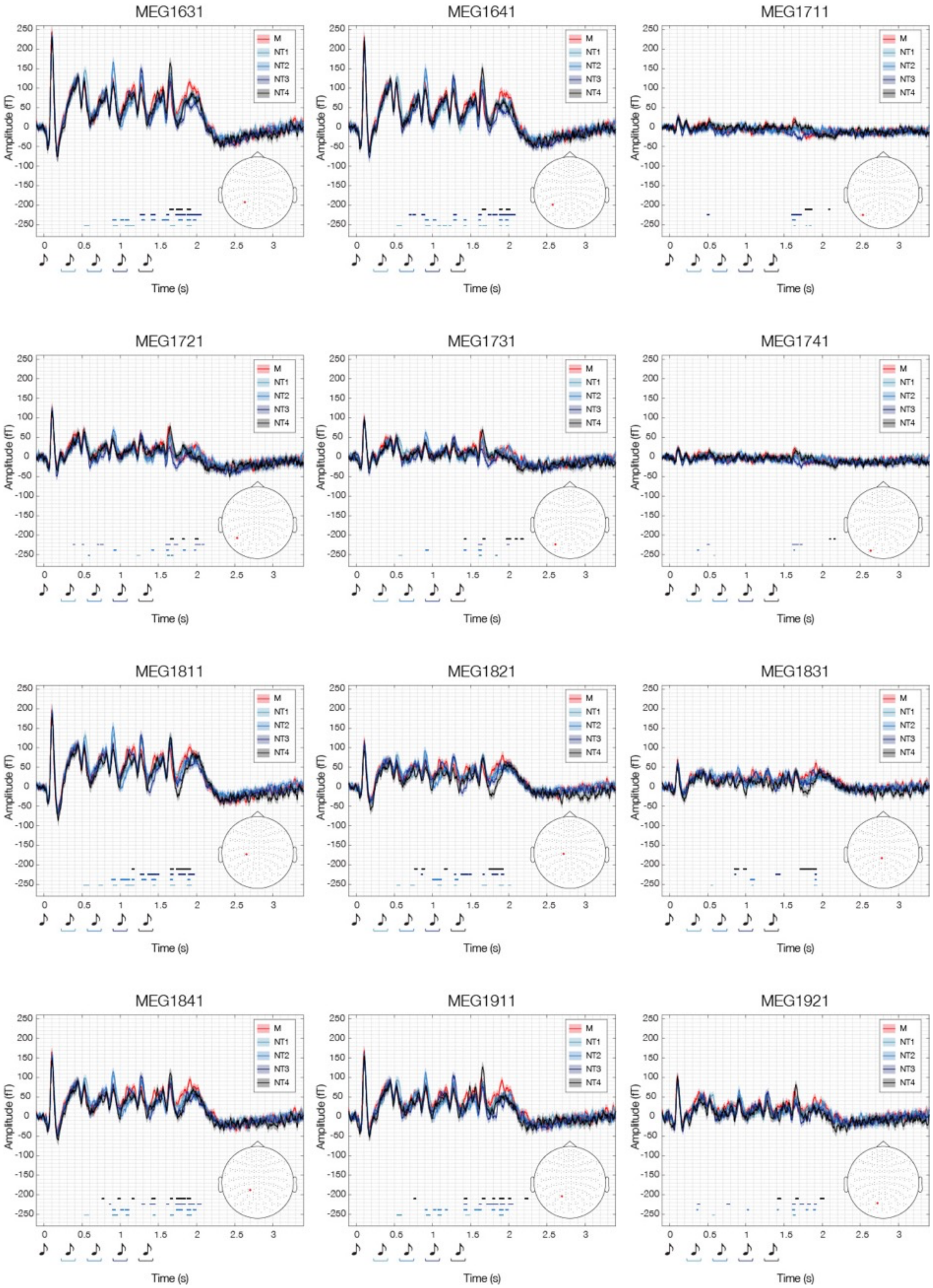
MEG1631
MEG1641
MEG1711
MEG1721
MEG1731
MEG1741
MEG1811
MEG1821
MEG1831
MEG1841
MEG1911
MEG1921
M
NT1
NT2
NT3
NT4
Amplitude (fT)
Time (s)

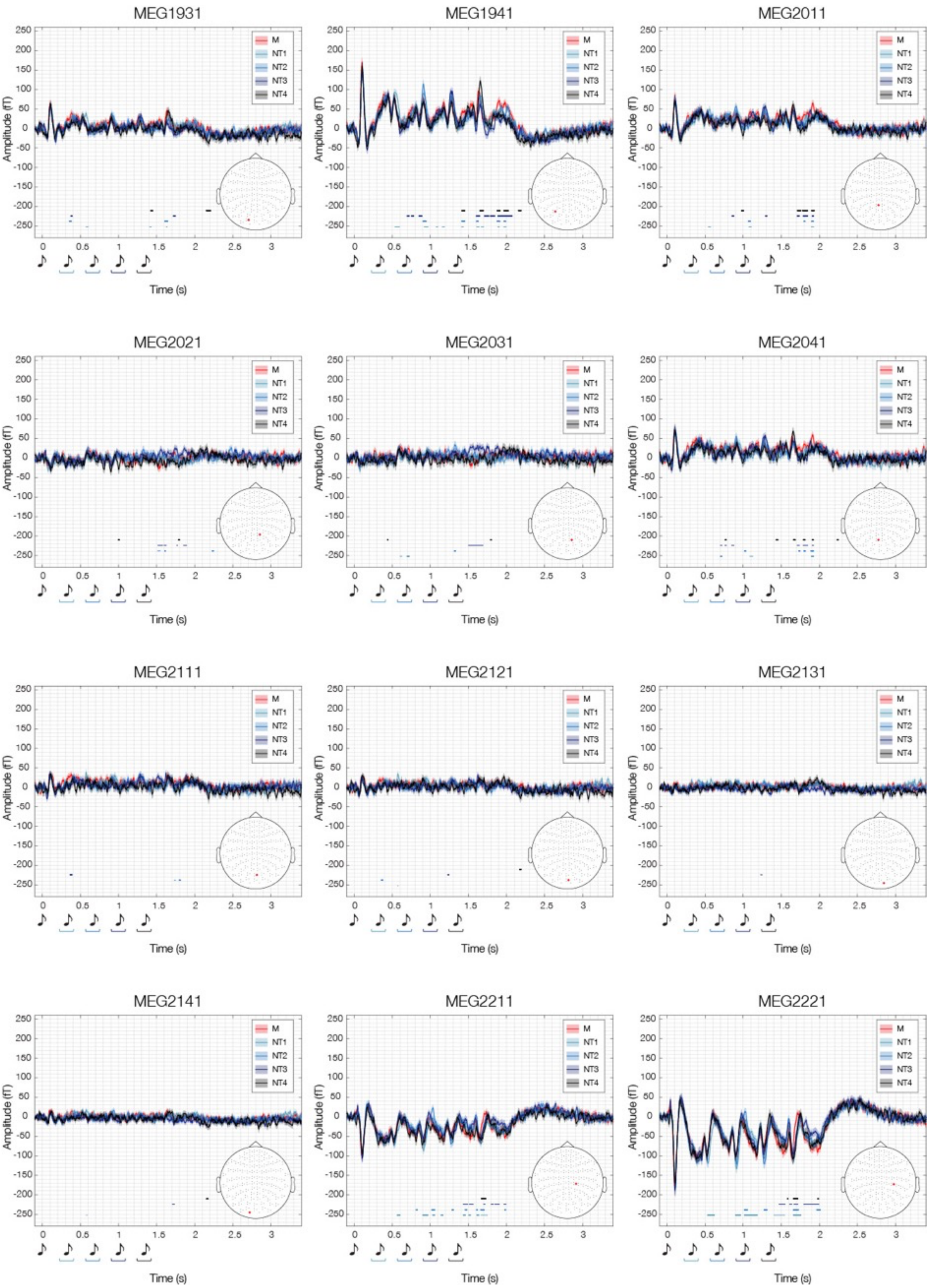
MEG1931
MEG1941
MEG2011
MEG2021
MEG2031
MEG2041
MEG2111
MEG2121
MEG2131
MEG2141
MEG2211
MEG2221
M
NT1
NT2
NT3
NT4
Amplitude (fT)
Time (s)

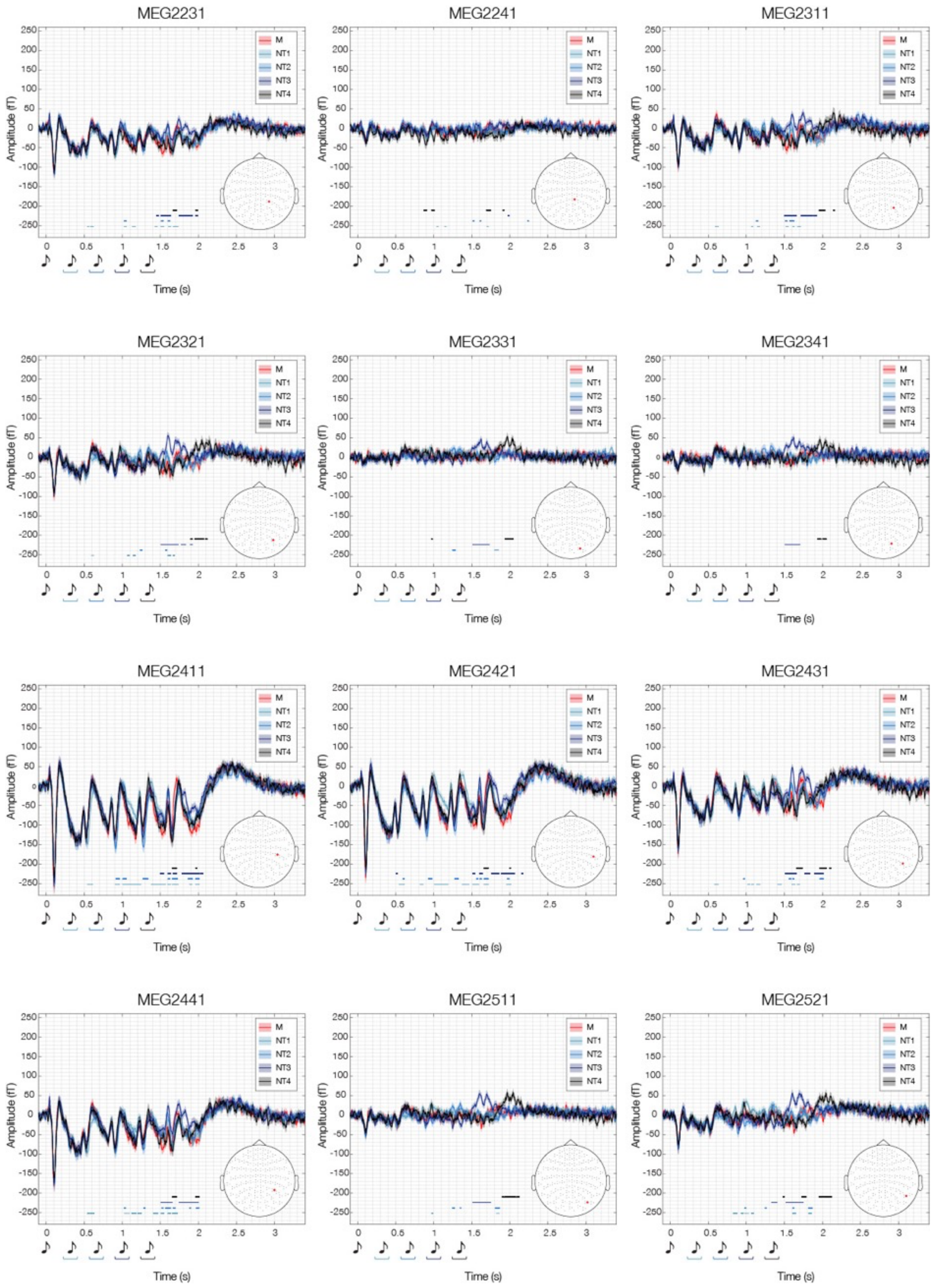
MEG2231
MEG2241
MEG2311
MEG2321
MEG2331
MEG2341
MEG2411
MEG2421
MEG2431
MEG2441
MEG2511
MEG2521
M
NT1
NT2
NT3
NT4
Amplitude (fT)
Time (s)

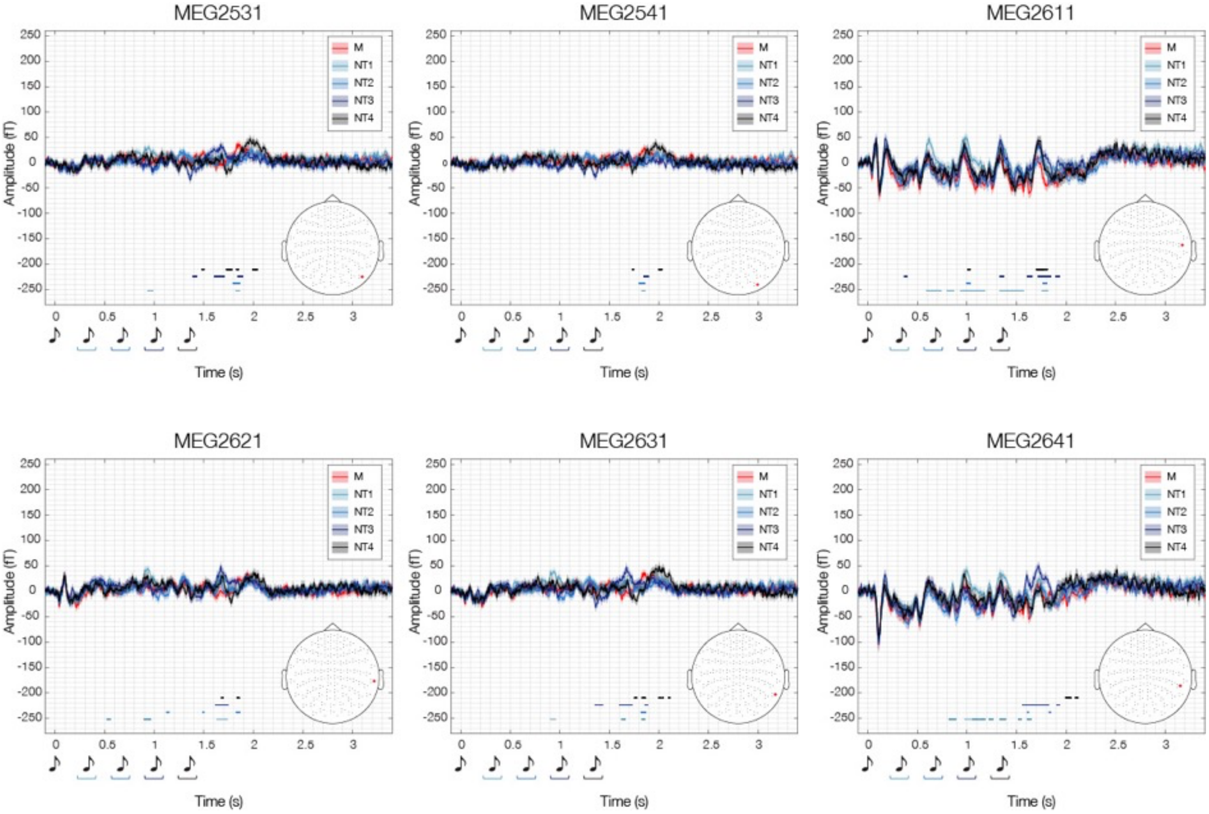
MEG2531
MEG2541
MEG2611
MEG2621
MEG2631
MEG2641
Amplitude (fT)
Time (s)
M
NT1
NT2
NT3
NT4

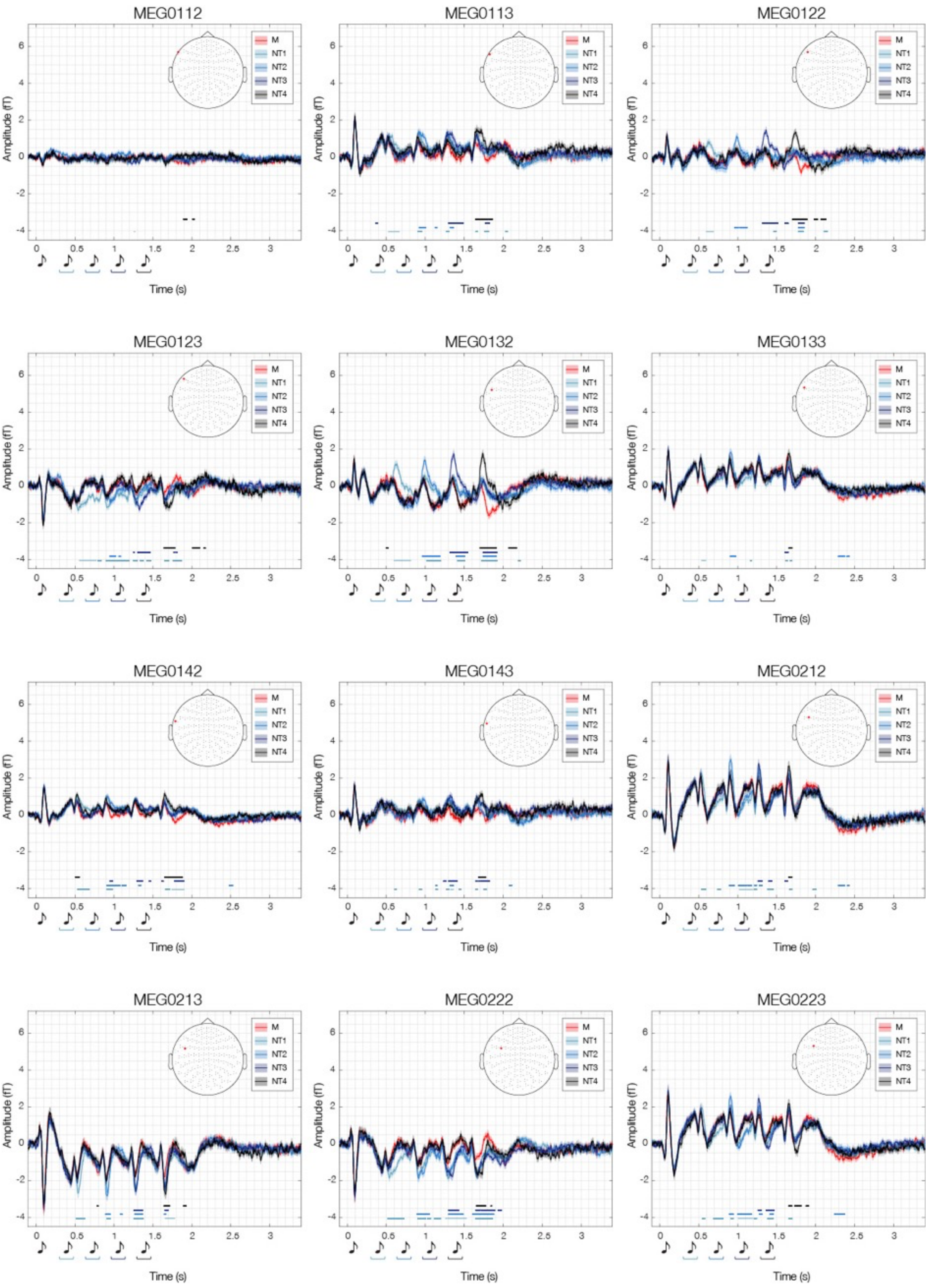
MEG0112
MEG0113
MEG0122
MEG0123
MEG0132
MEG0133
MEG0142
MEG0143
MEG0212
MEG0213
MEG0222
MEG0223
M
NT1
NT2
NT3
NT4
Amplitude (fT)
Time (s)

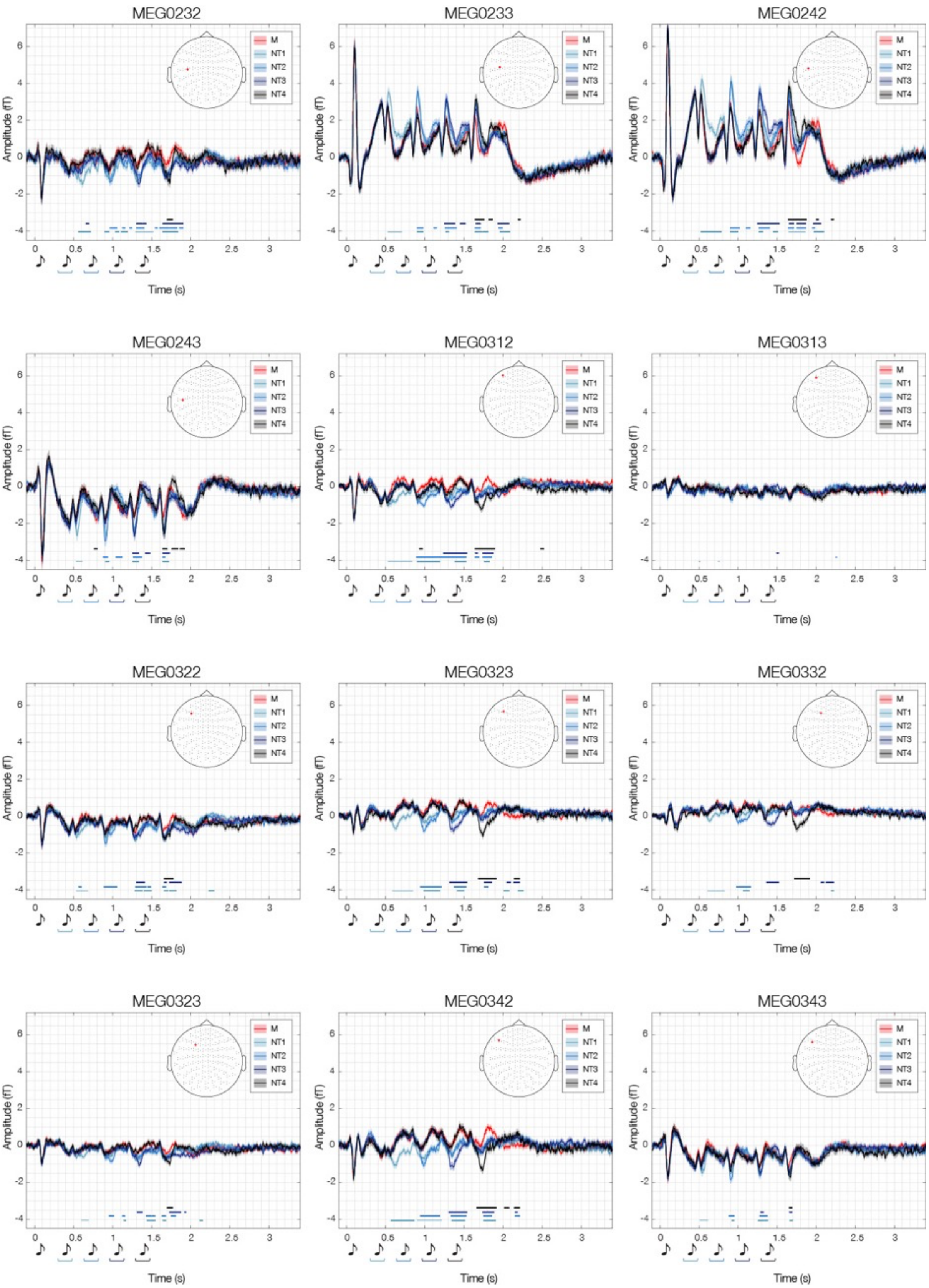
MEG0232
MEG0233
MEG0242
MEG0243
MEG0312
MEG0313
MEG0322
MEG0323
MEG0332
MEG0323
MEG0342
MEG0343
M
NT1
NT2
NT3
NT4
Amplitude (fT)
Time (s)

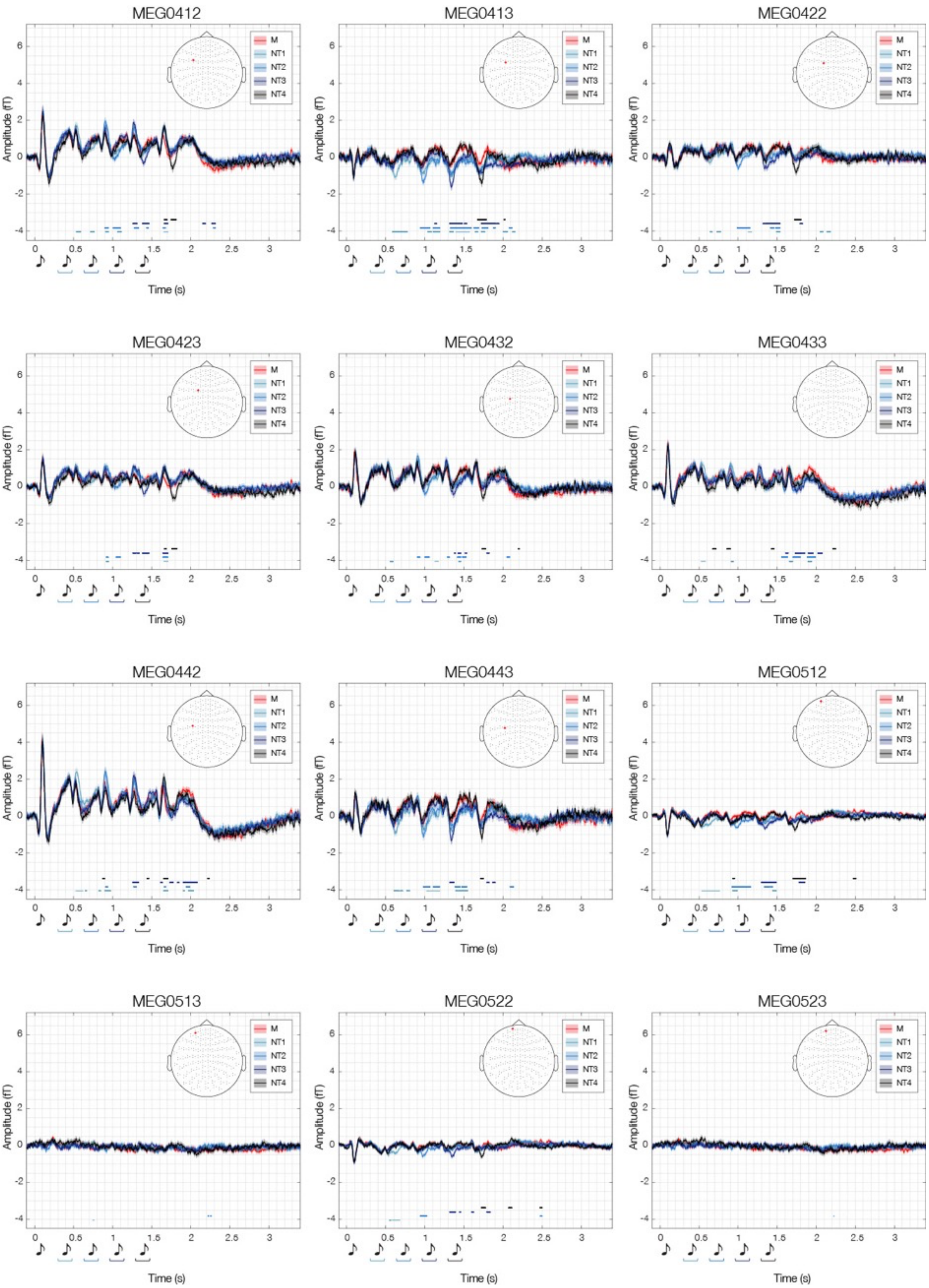
MEG0412
MEG0413
MEG0422
MEG0423
MEG0432
MEG0433
MEG0442
MEG0443
MEG0512
MEG0513
MEG0522
MEG0523
M
NT1
NT2
NT3
NT4
Amplitude (fT)
Time (s)
6
4
2
0
-2
-4
0
0.5
1
1.5
2
2.5
3

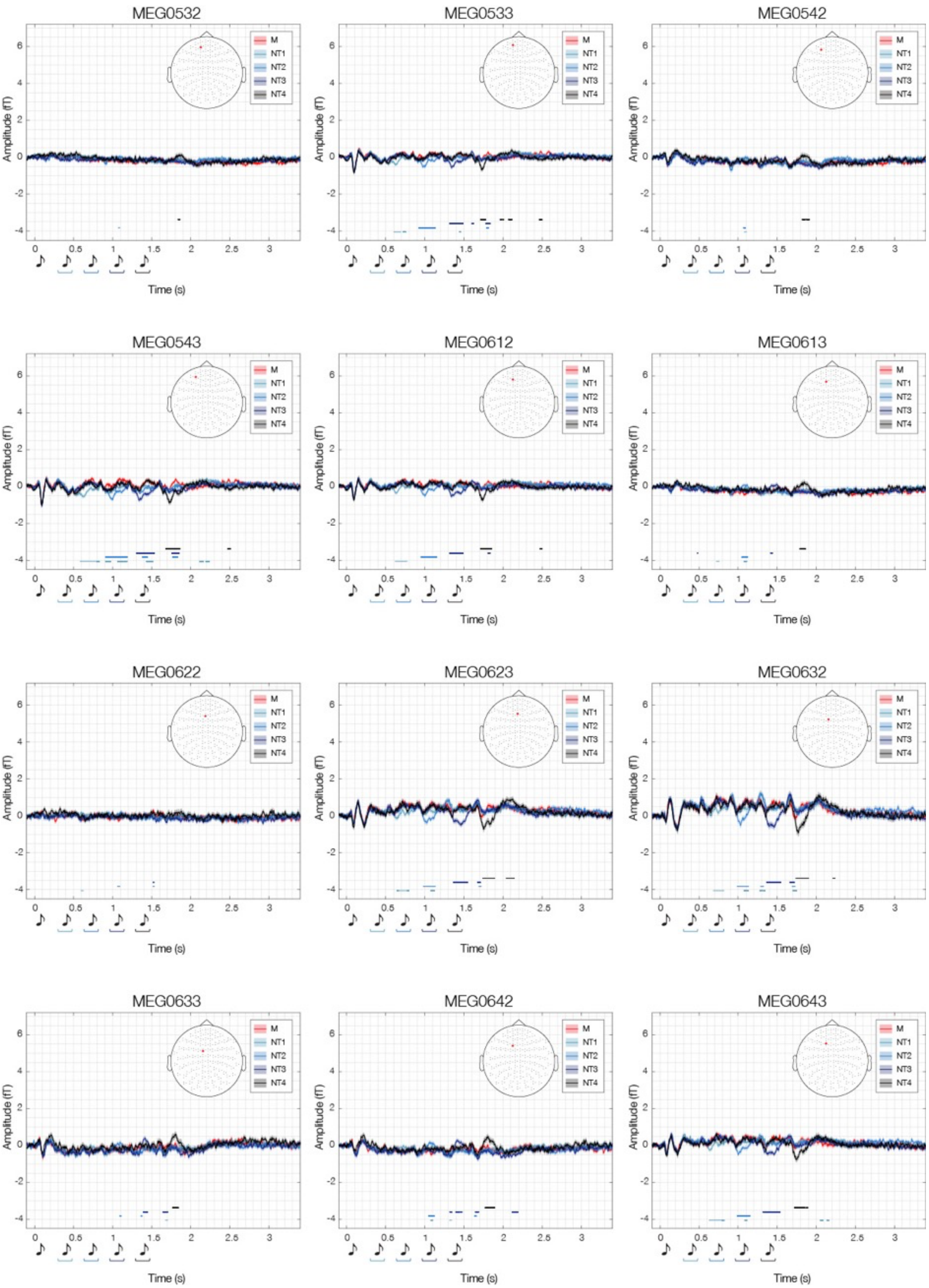
MEG0532
MEG0533
MEG0542
MEG0543
MEG0612
MEG0613
MEG0622
MEG0623
MEG0632
MEG0633
MEG0642
MEG0643
M
NT1
NT2
NT3
NT4
Amplitude (fT)
Time (s)

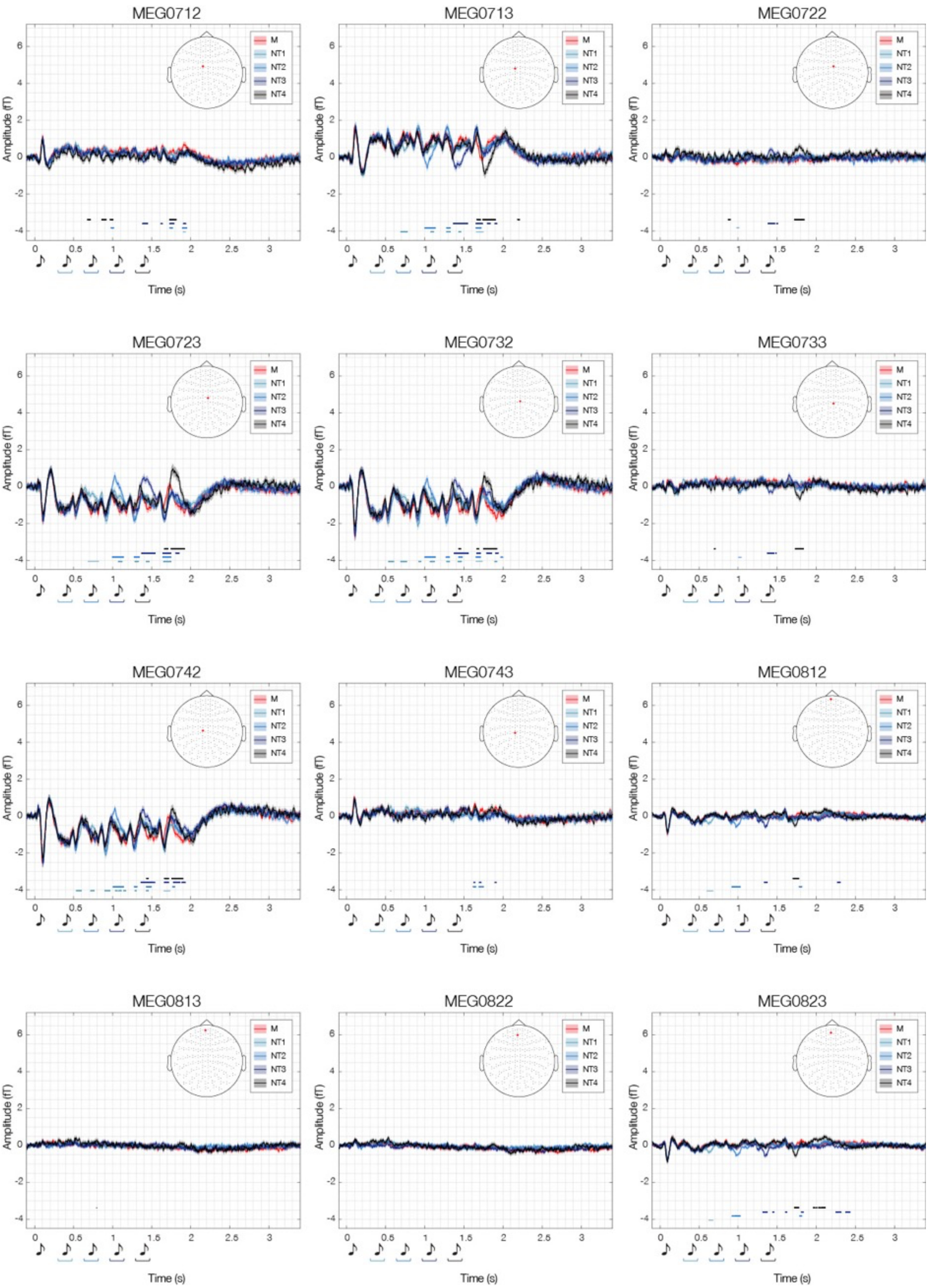
MEG0712
MEG0713
MEG0722
MEG0723
MEG0732
MEG0733
MEG0742
MEG0743
MEG0812
MEG0813
MEG0822
MEG0823
M
NT1
NT2
NT3
NT4
Amplitude (fT)
Time (s)

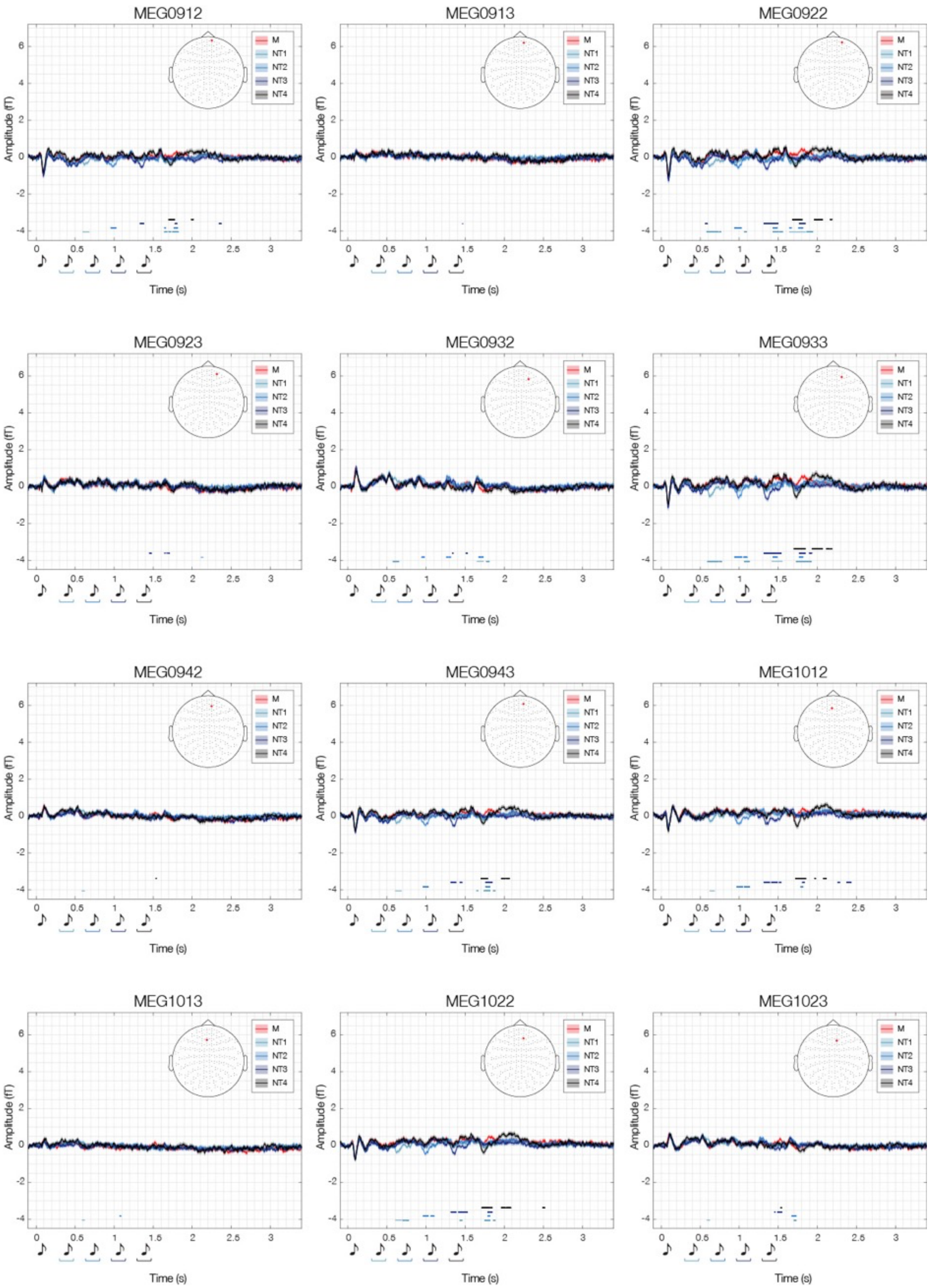
MEG0912
MEG0913
MEG0922
MEG0923
MEG0932
MEG0933
MEG0942
MEG0943
MEG1012
MEG1013
MEG1022
MEG1023
M
NT1
NT2
NT3
NT4
Amplitude (fT)
Time (s)

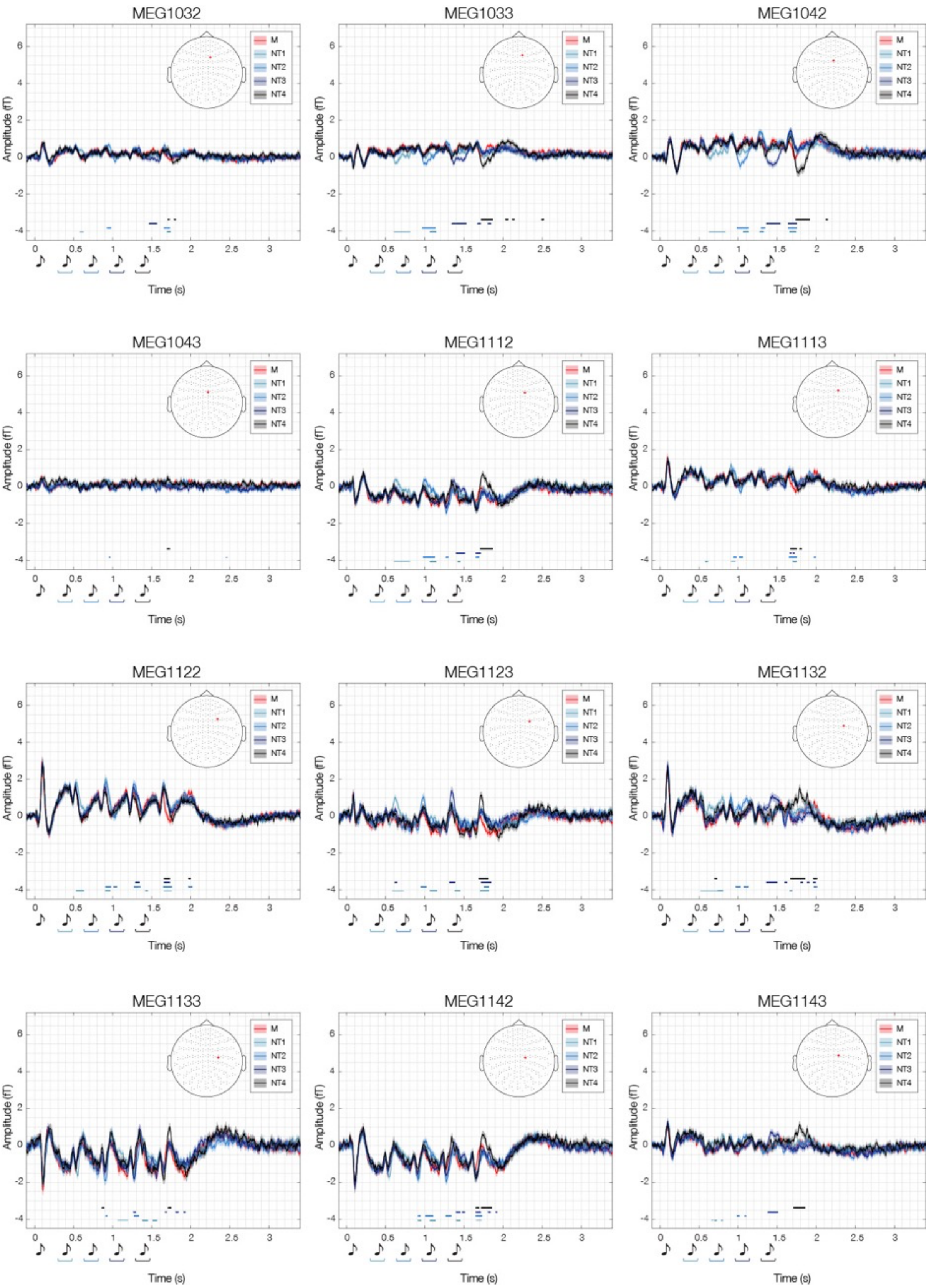
MEG1032
MEG1033
MEG1042
MEG1043
MEG1112
MEG1113
MEG1122
MEG1123
MEG1132
MEG1133
MEG1142
MEG1143
M
NT1
NT2
NT3
NT4
Amplitude (fT)
Time (s)

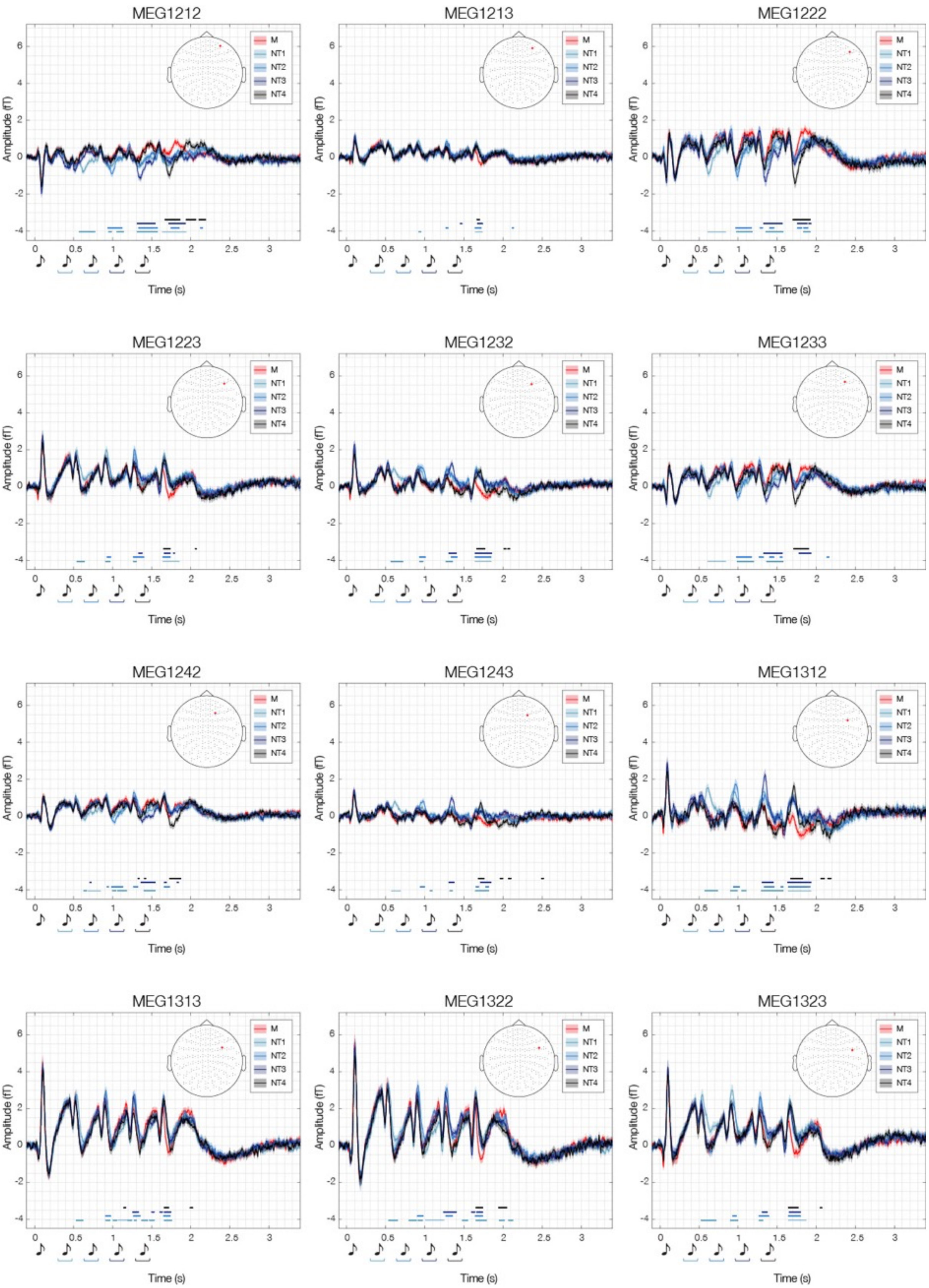
MEG1212
MEG1213
MEG1222
MEG1223
MEG1232
MEG1233
MEG1242
MEG1243
MEG1312
MEG1313
MEG1322
MEG1323
M
NT1
NT2
NT3
NT4
Amplitude (fT)
Time (s)

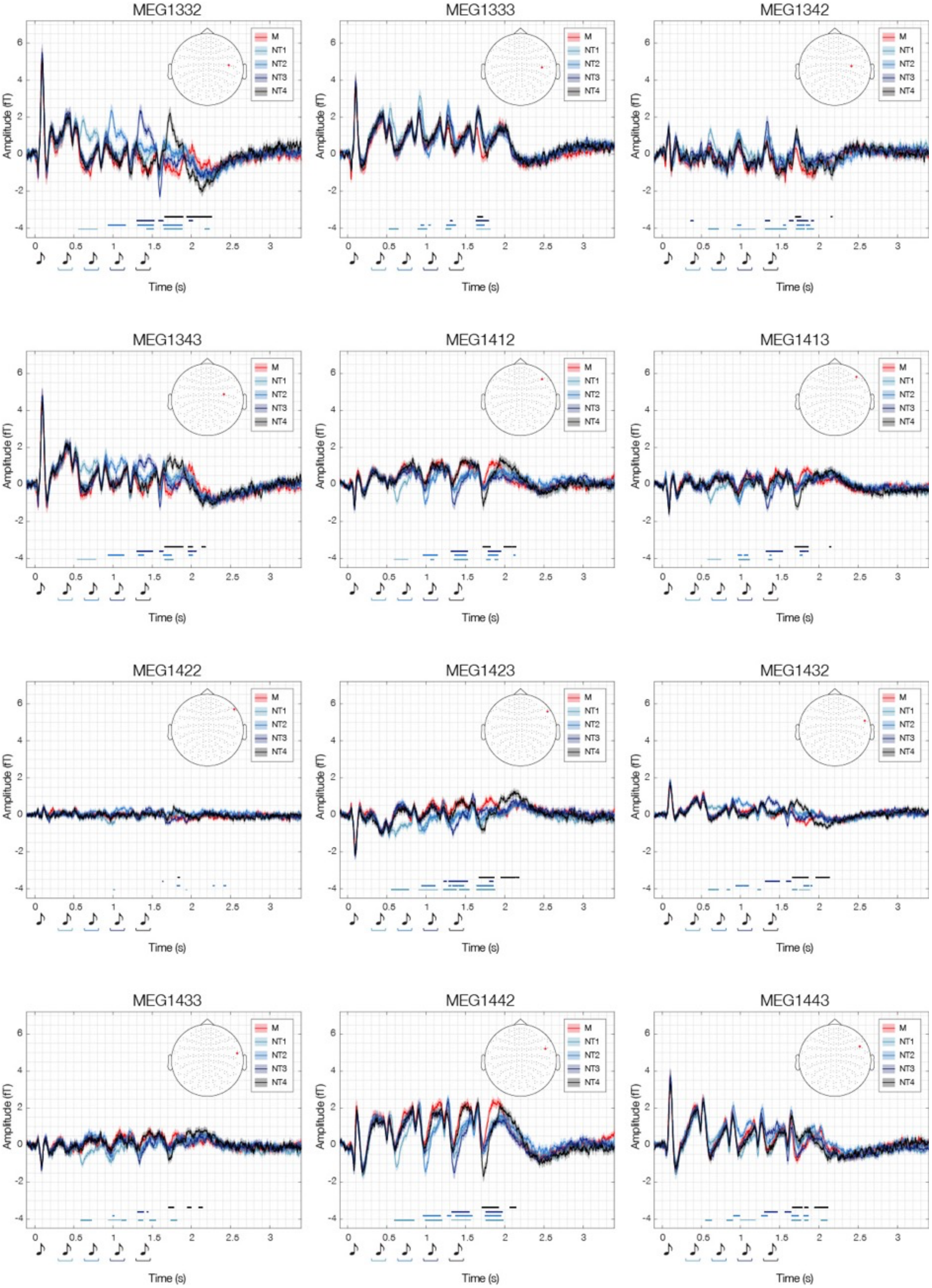
MEG1332
MEG1333
MEG1342
MEG1343
MEG1412
MEG1413
MEG1422
MEG1423
MEG1432
MEG1433
MEG1442
MEG1443
M
NT1
NT2
NT3
NT4
Amplitude (fT)
Time (s)

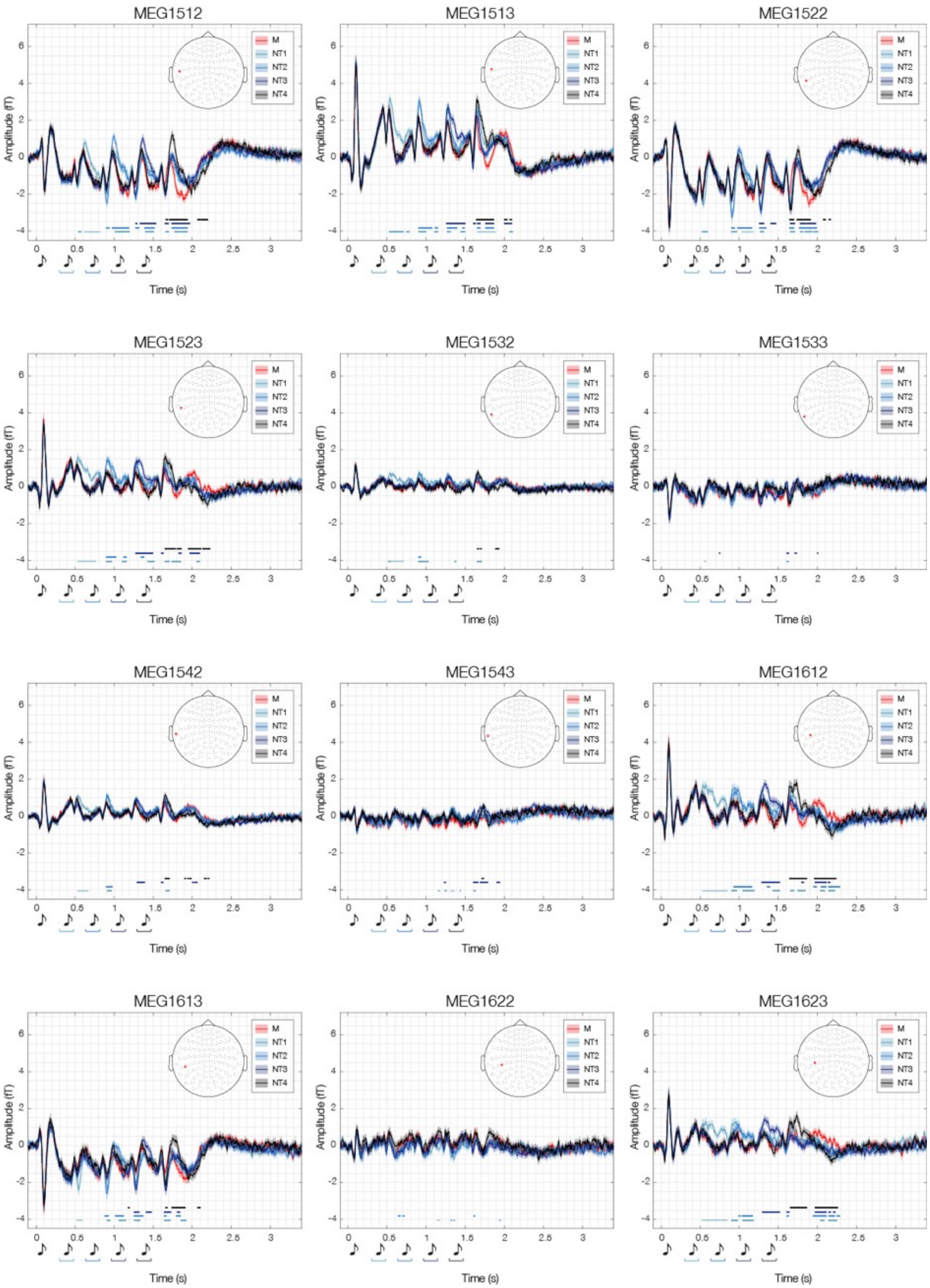

MEG1512
MEG1513
MEG1522
MEG1523
MEG1532
MEG1533
MEG1542
MEG1543
MEG1612
MEG1613
MEG1622
MEG1623
M
NT1
NT2
NT3
NT4
Amplitude (fT)
Time (s)

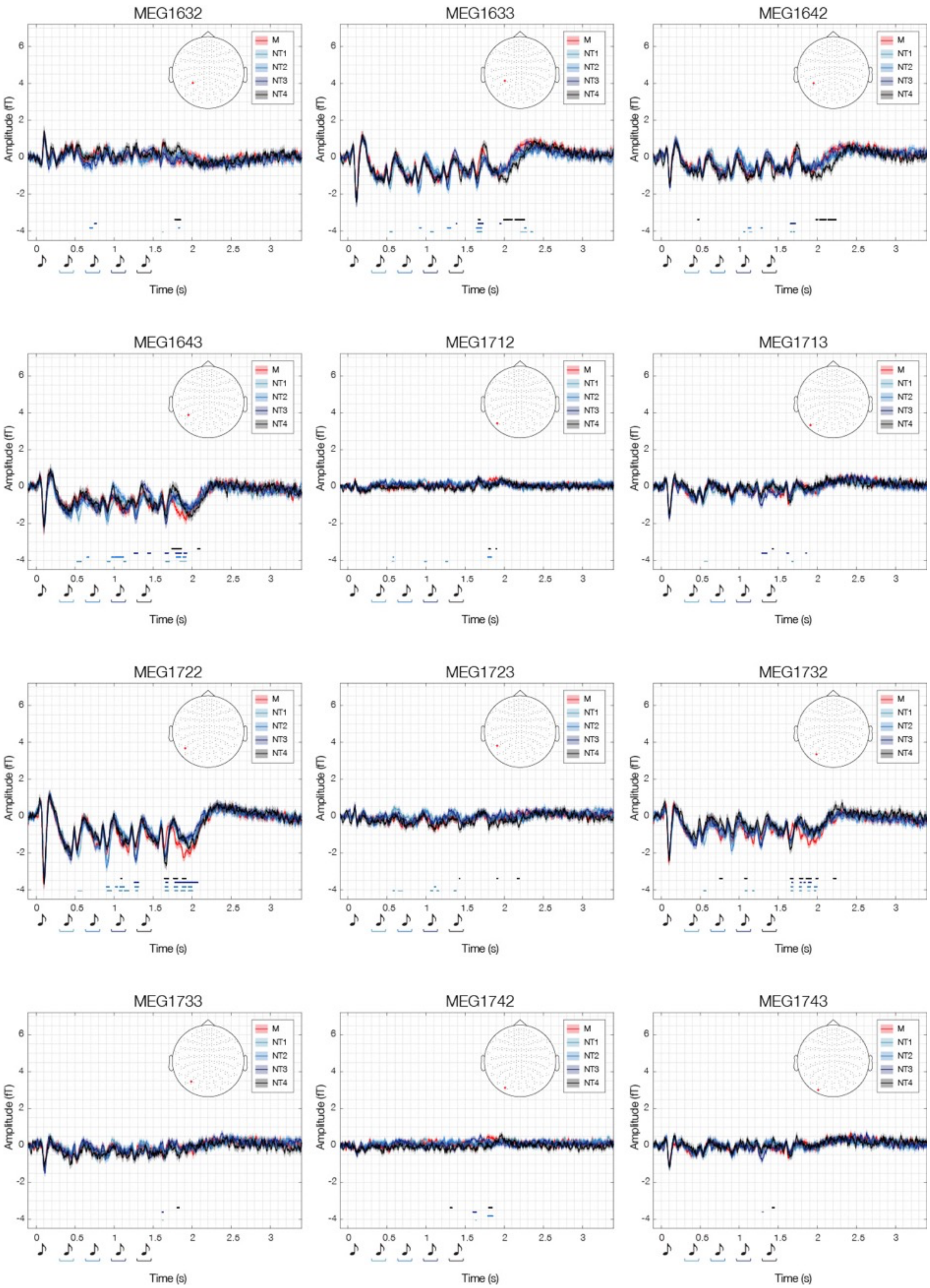
MEG1632
MEG1633
MEG1642
MEG1643
MEG1712
MEG1713
MEG1722
MEG1723
MEG1732
MEG1733
MEG1742
MEG1743
M
NT1
NT2
NT3
NT4
Amplitude (fT)
Time (s)

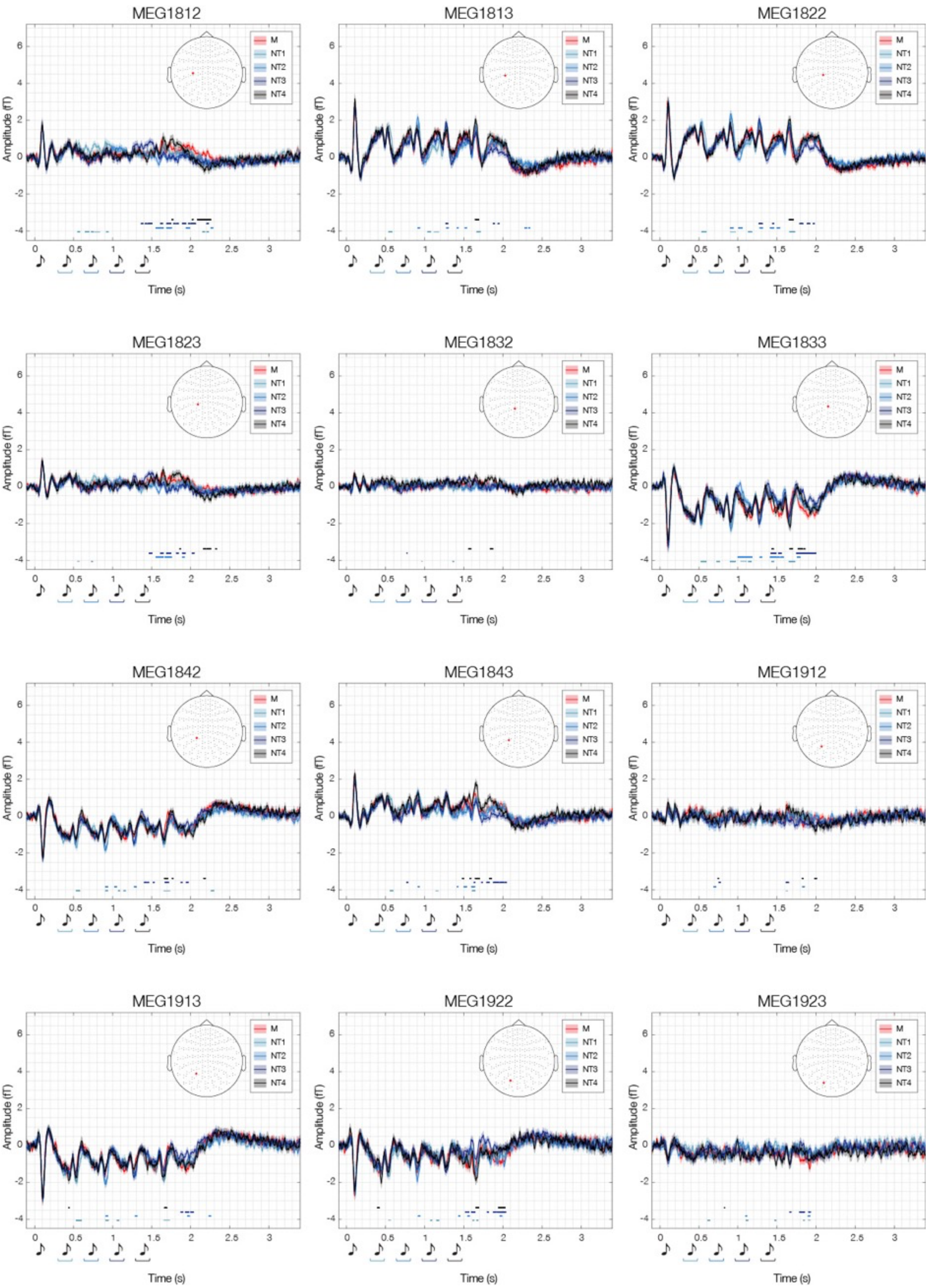
MEG1812
MEG1813
MEG1822
MEG1823
MEG1832
MEG1833
MEG1842
MEG1843
MEG1912
MEG1913
MEG1922
MEG1923
M
NT1
NT2
NT3
NT4
Amplitude (fT)
Time (s)

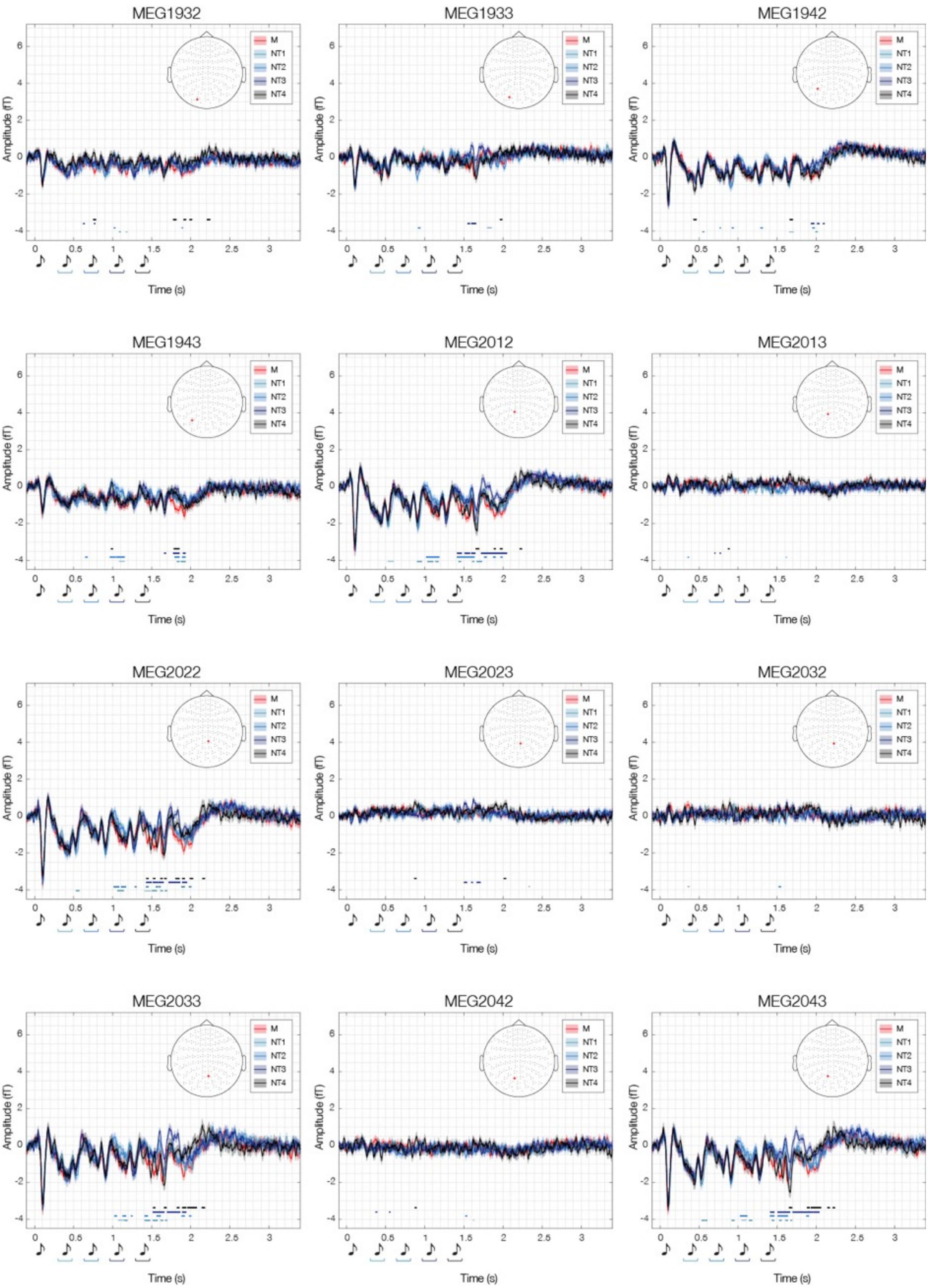
MEG1932
MEG1933
MEG1942
MEG1943
MEG2012
MEG2013
MEG2022
MEG2023
MEG2032
MEG2033
MEG2042
MEG2043
M
NT1
NT2
NT3
NT4
Amplitude (fT)
Time (s)

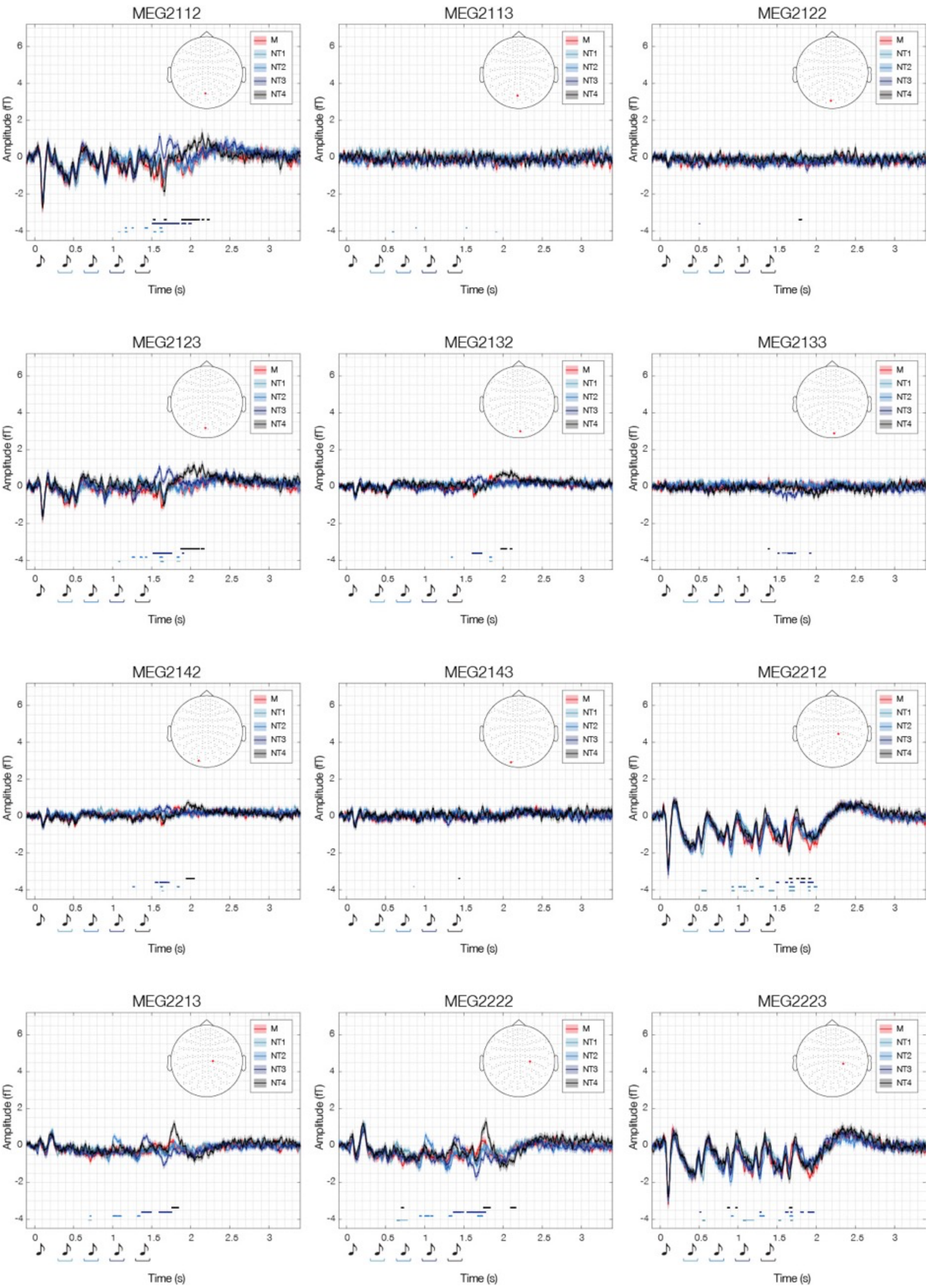
MEG2112
MEG2113
MEG2122
MEG2123
MEG2132
MEG2133
MEG2142
MEG2143
MEG2212
MEG2213
MEG2222
MEG2223
M
NT1
NT2
NT3
NT4
Amplitude (fT)
Time (s)
6
4
2
0
-2
-4
0
0.5
1
1.5
2
2.5
3

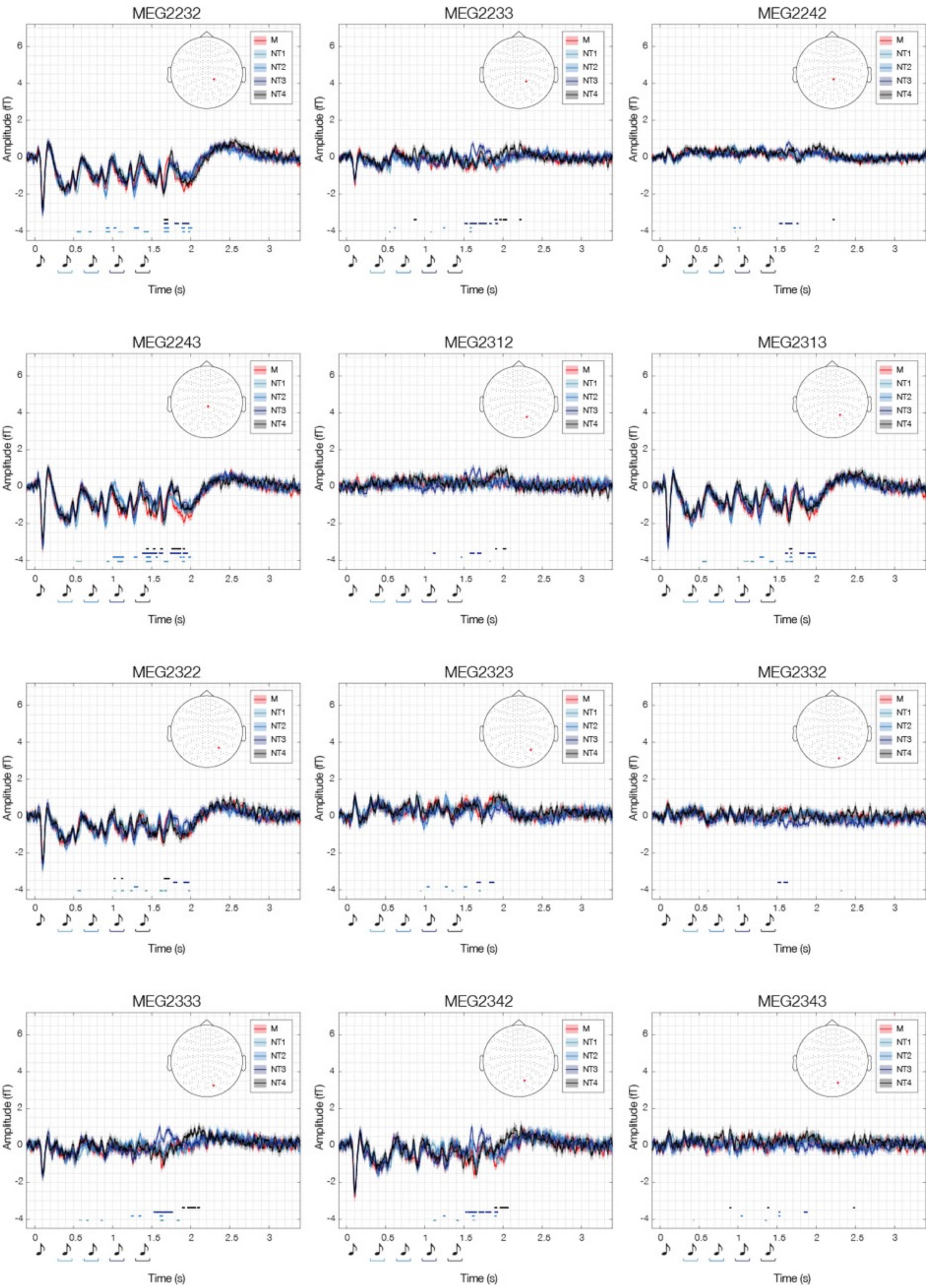
MEG2232
MEG2233
MEG2242
MEG2243
MEG2312
MEG2313
MEG2322
MEG2323
MEG2332
MEG2333
MEG2342
MEG2343
M
NT1
NT2
NT3
NT4
Amplitude (fT)
Time (s)

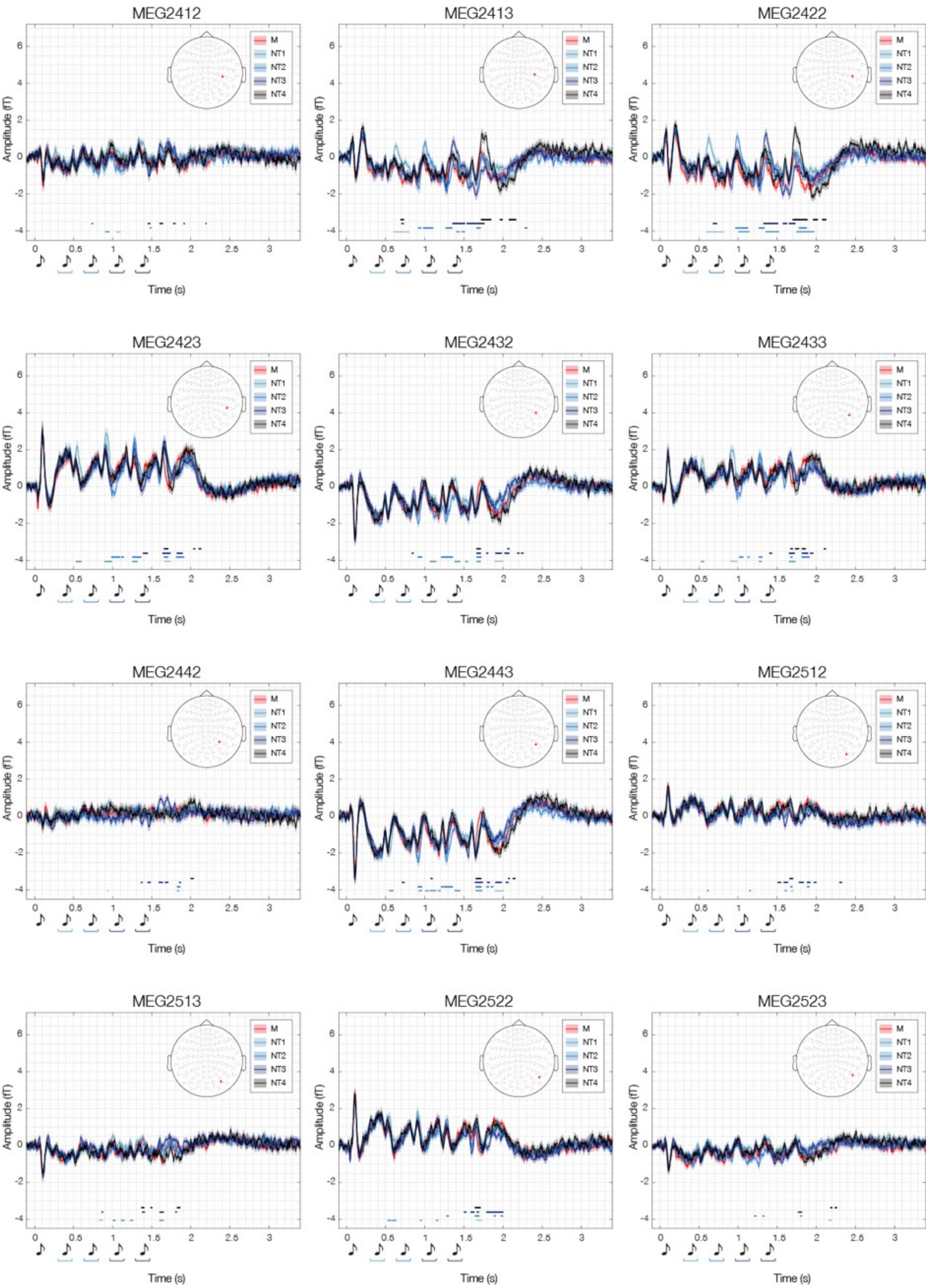
MEG2412
MEG2413
MEG2422
MEG2423
MEG2432
MEG2433
MEG2442
MEG2443
MEG2512
MEG2513
MEG2522
MEG2523
M
NT1
NT2
NT3
NT4
Amplitude (fT)
Time (s)

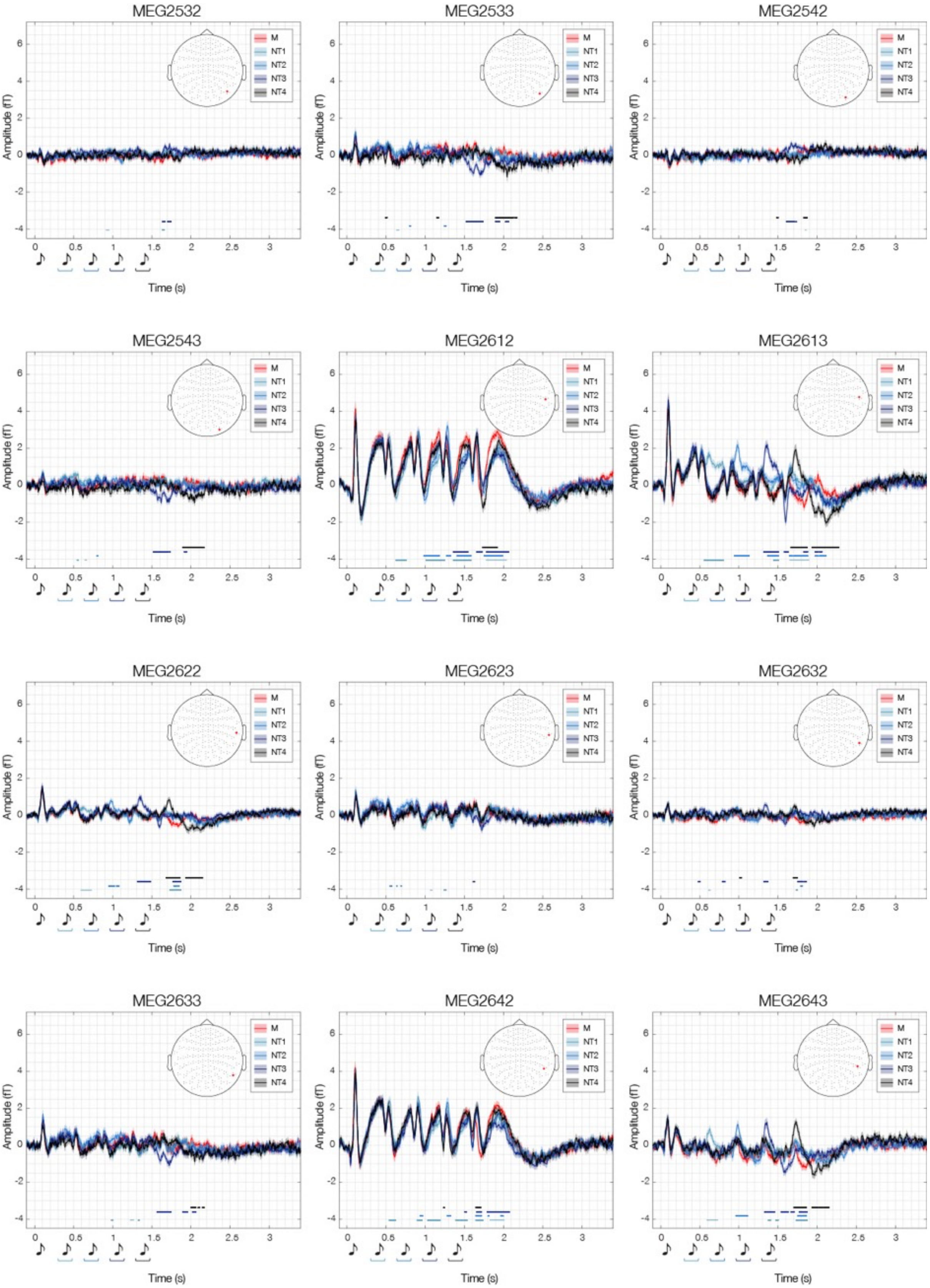
MEG2532
MEG2533
MEG2542
MEG2543
MEG2612
MEG2613
MEG2622
MEG2623
MEG2632
MEG2633
MEG2642
MEG2643
M
NT1
NT2
NT3
NT4
Amplitude (fT)
Time (s)

Figure S5$_{a\text{-}z2}$. Evoked responses across experimental conditions: all magnetoencephalography (MEG) channels

*This figure illustrates the univariate analysis contrasting the brain activity associated with memorised (M) versus each category of novel (N) sequences (i.e. M versus novel T1 [NT1], M versus novel T2 [NT2], M versus novel T3 [NT3], M versus novel T4 [NT4]) computed independently on each magnetometer and gradiometer MEG channel. These contrasts were computed using two-sided t-tests and corrected for multiple comparisons employing cluster-based Monte-Carlo simulations (MCS; α = .05, p-value = .001). The figure shows the time series averaged over participants (n = 83), while the shaded areas represent standard errors. Musical tone sketches indicate the onset of the sounds forming the sequences. Blue-black lines highlight significant differences between M and Ns. Different hues of blue or black correspond to specific M versus N condition comparisons. For example, lightest blue represents M versus NT1, the second lightest blue represents M versus NT2, and so on. The Figure is arranged into 27 subfigures to illustrate each of the 306 MEG channels with adequate resolution. For a detailed statistical report on significant differences between experimental conditions in all MEG channels, consult **Supplementary Data 4**.*

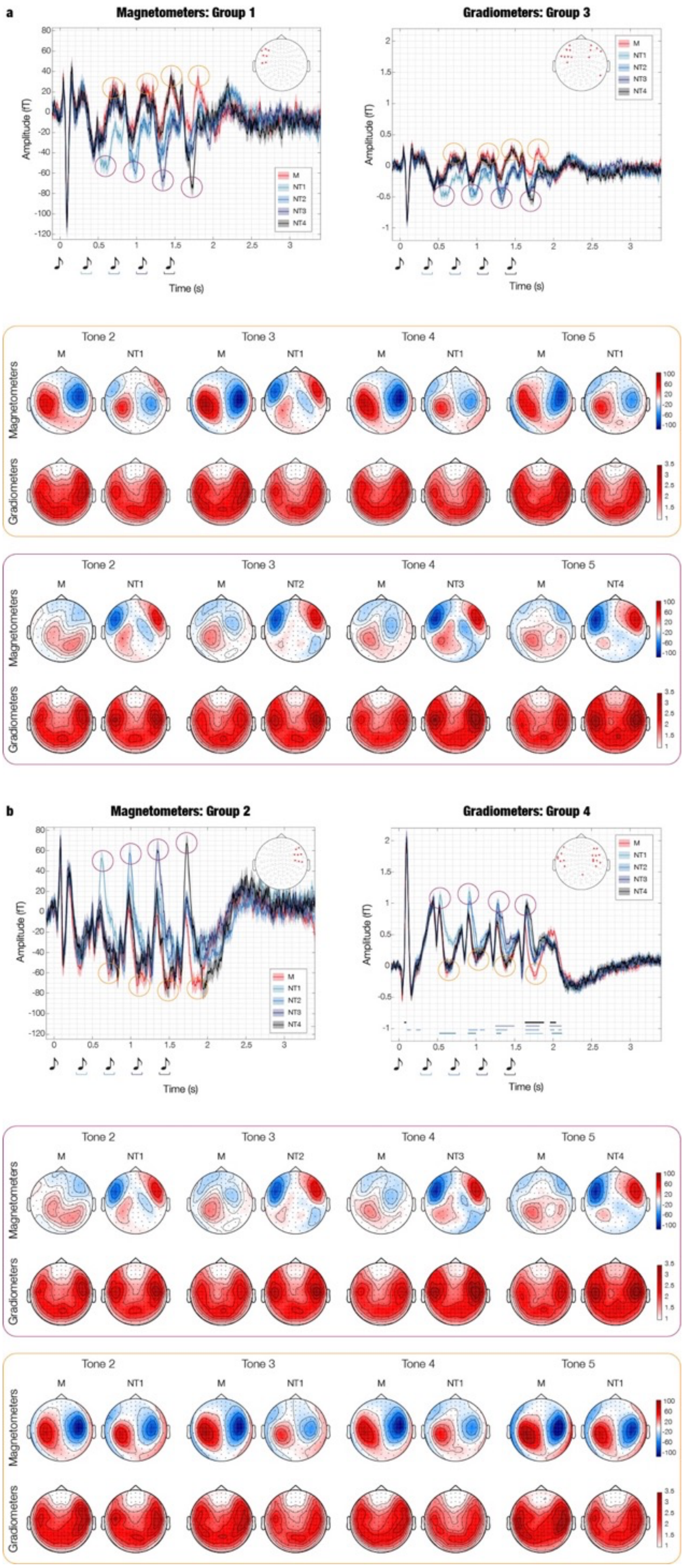
a
Magnetometers: Group 1
Gradiometers: Group 3
Amplitude (fT)
Time (s)
M
NT1
NT2
NT3
NT4
Tone 2
Tone 3
Tone 4
Tone 5
Magnetometers
Gradiometers
b
Magnetometers: Group 2
Gradiometers: Group 4

Figure S6. Topographic maps of the neural activity peaks

a - *Time series averaged over participants (n = 83) and grouped as follows: (i) magnetometers negative N100 (left), and (iii) gradiometers negative N100, exactly as presented in* ***Figure 2****. Shaded areas represent standard errors. M indicates memorised, while NT1, NT2, NT3, NT4 denote novel T1, novel T2, novel T3, novel T4, respectively. Blue-black lines highlight significant differences between M and Ns, obtained by computing two-sided t-tests, independently for each time-point, and correcting for multiple comparisons using one-dimensional Monte-Carlo simulations (MCS; MCS, α = .05, MCS p-value = .001). Notably, here we have marked the maximum and minimum peaks of neural activity following the onset of each tone (excluding the first tone). These peaks occurred approximately at 150-250 ms (purple circle) and 300-350 ms (yellow circle) after each tone's onset. The yellow and purple boxes show the topographic representation of the neural activity averaged within a time window of ±20 ms around each of the peaks indicated by the yellow and purple circles in the time series above. The yellow box shows the magnetometers (fT) and the combined planar gradiometers (fT/mm) for tones 2-5. For each tone, the left topographic maps relate to the M condition, while the left ones to the NT1 condition. The purple box follows a similar concept, but here, each pair of topographic maps within each tone includes the left plots representing the Ns conditions, and the right ones representing the M condition.* ***b –*** *This part replicates the representation in* ***a)*** *on two different groups of MEG channels: (ii) magnetometers positive N100 (left), and (iv): gradiometers positive N100.*

Left auditory cortex

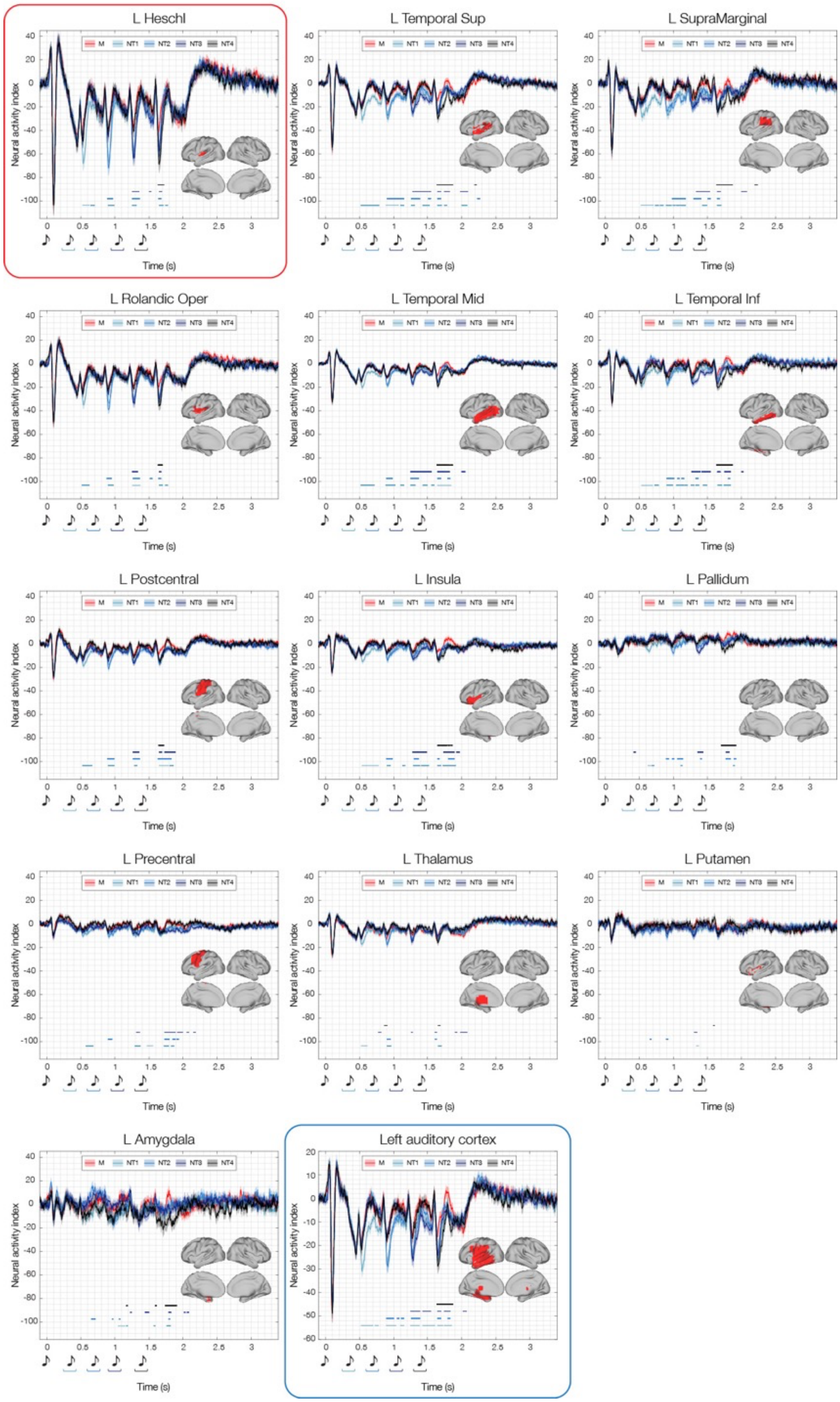

Right auditory cortex

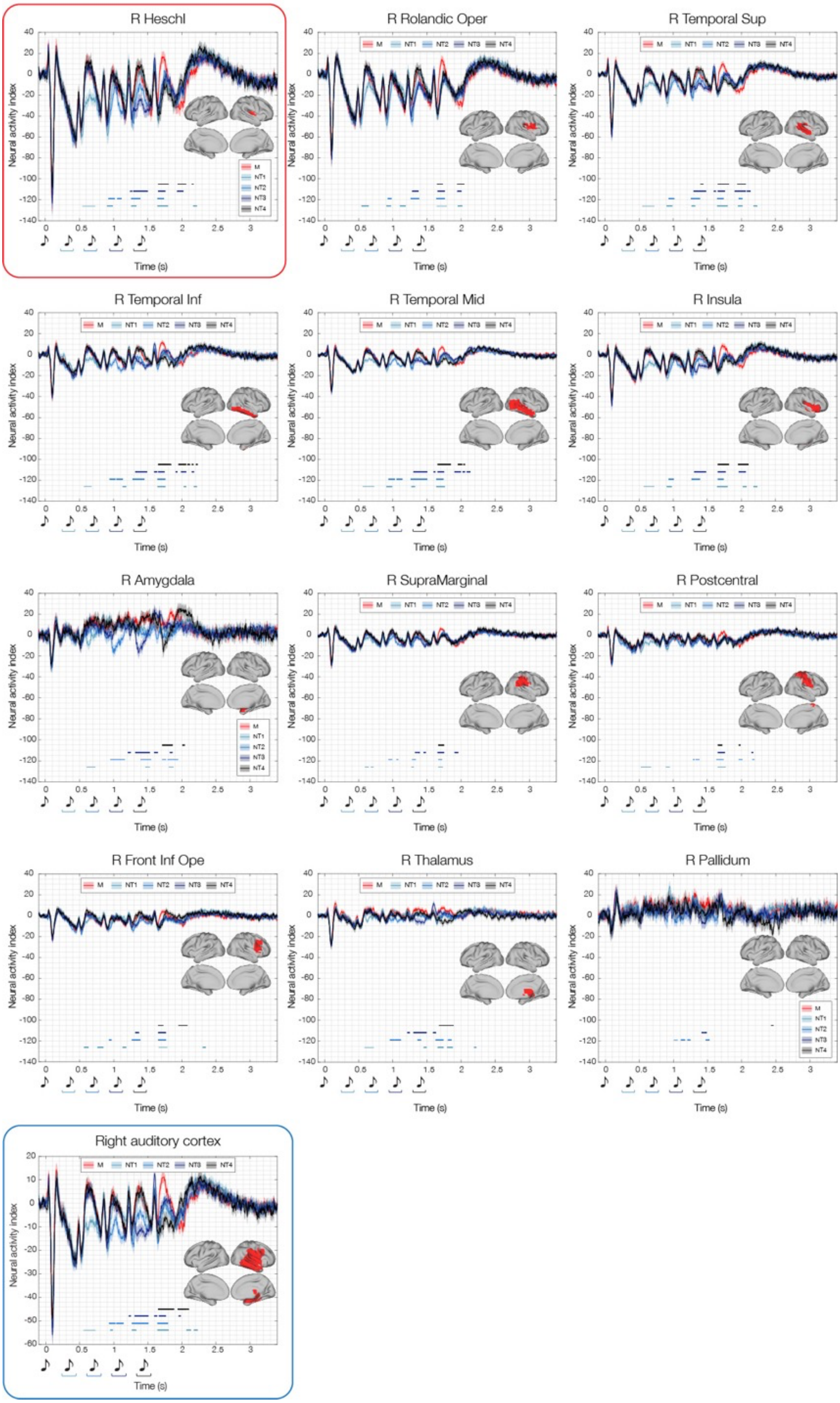

Left hippocampal and inferior temporal regions

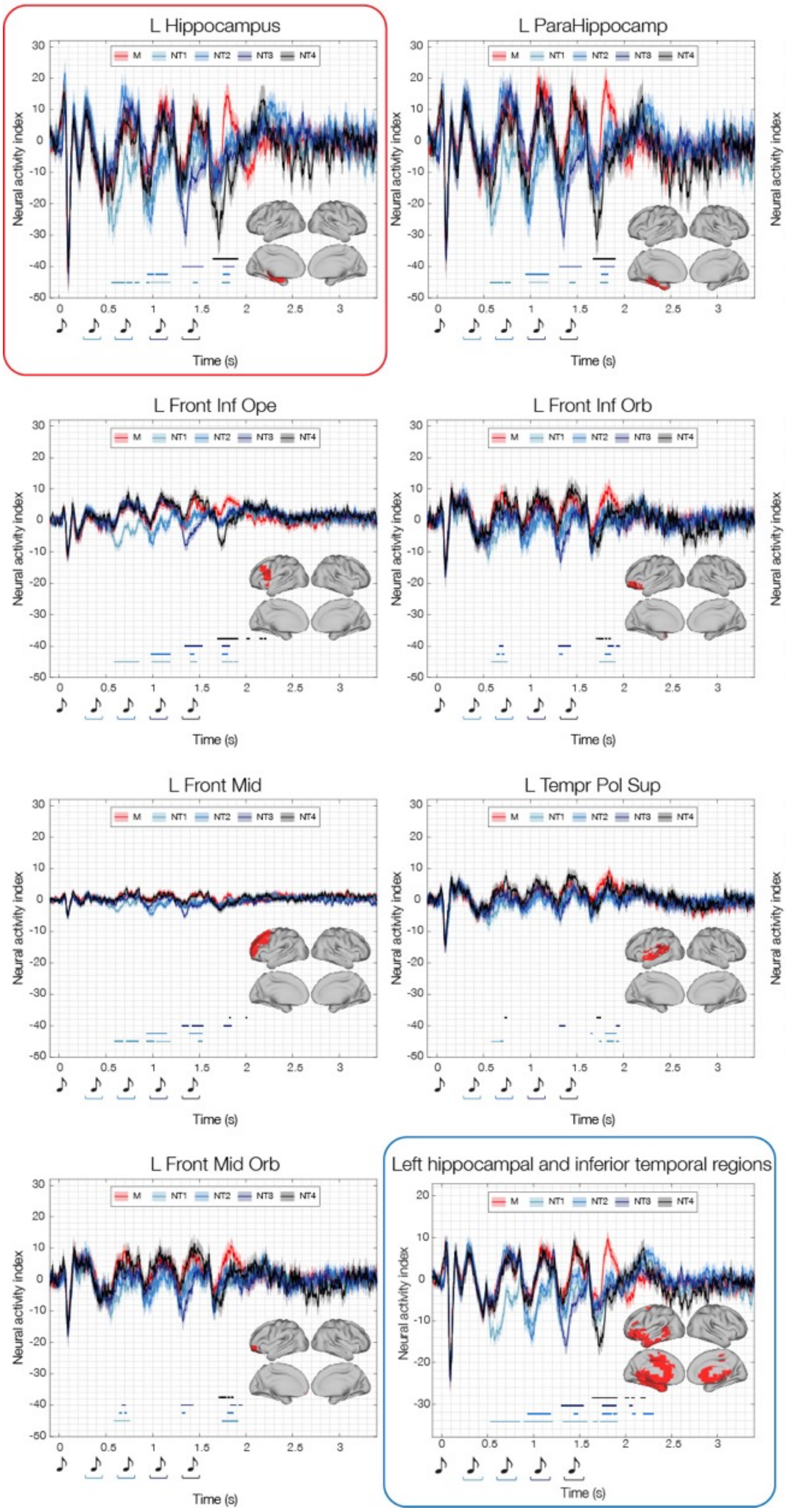

Right hippocampal and inferior temporal regions

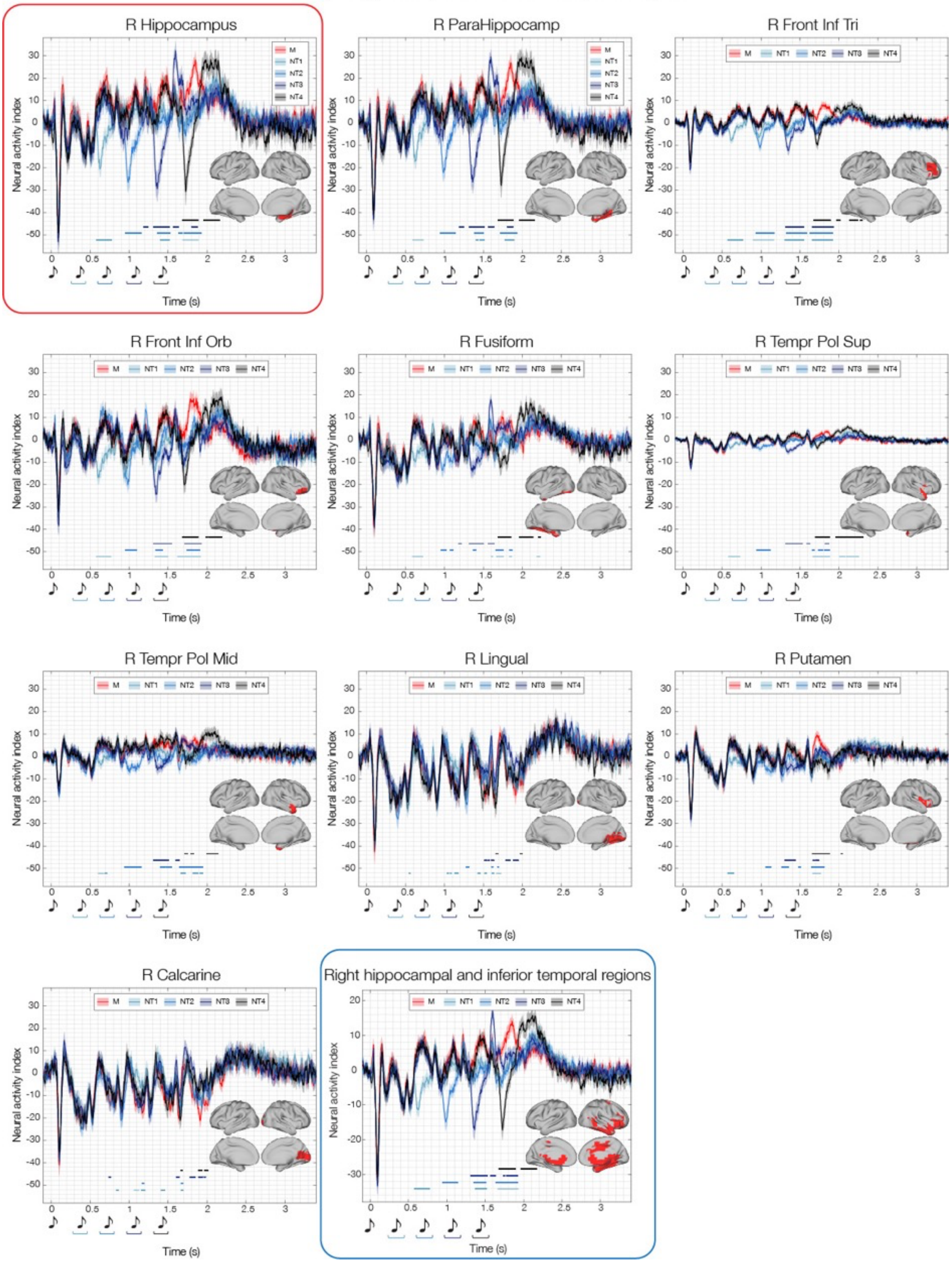

Medial cingulate gyrus

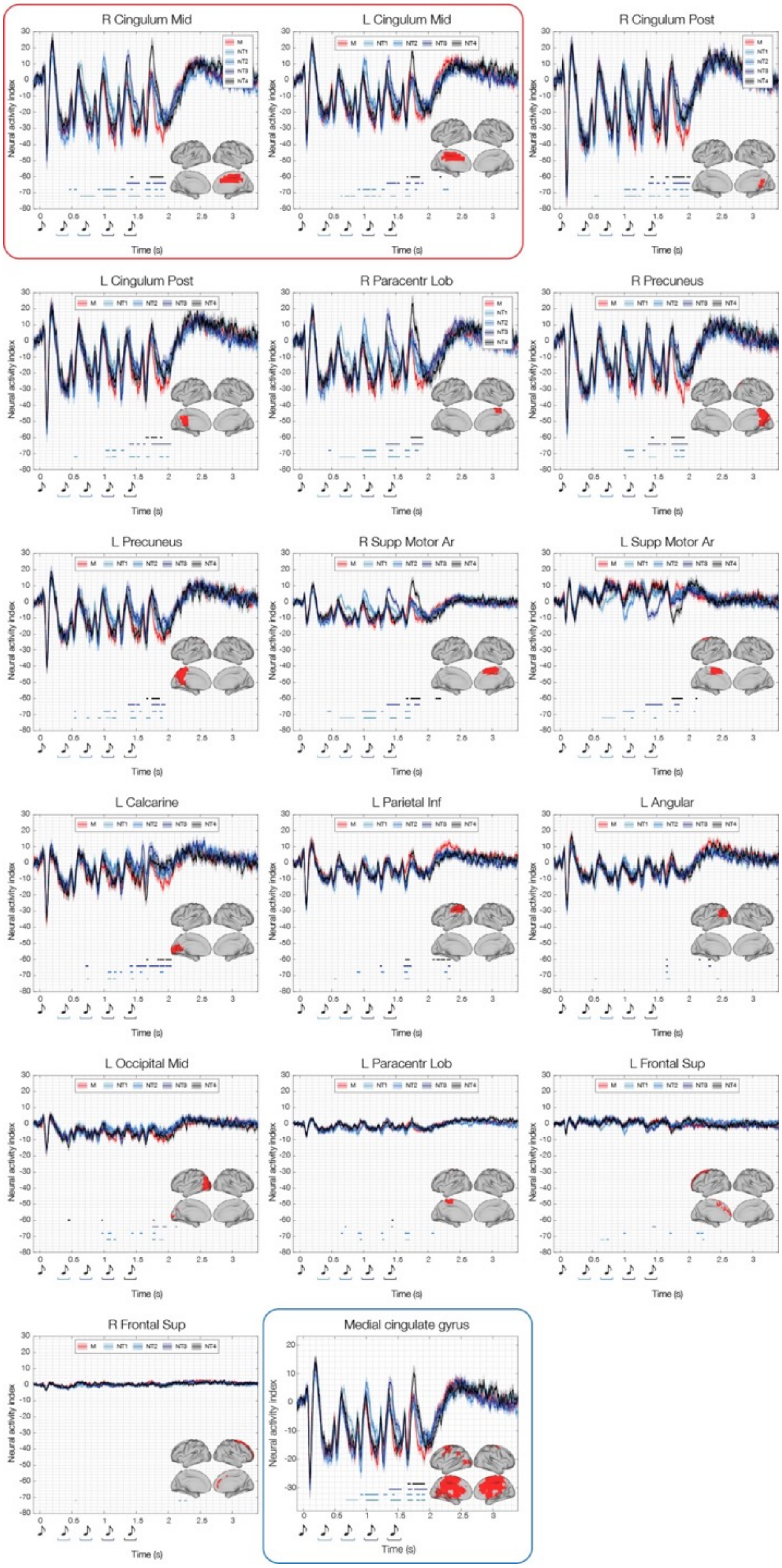

Ventromedial prefrontal cortex

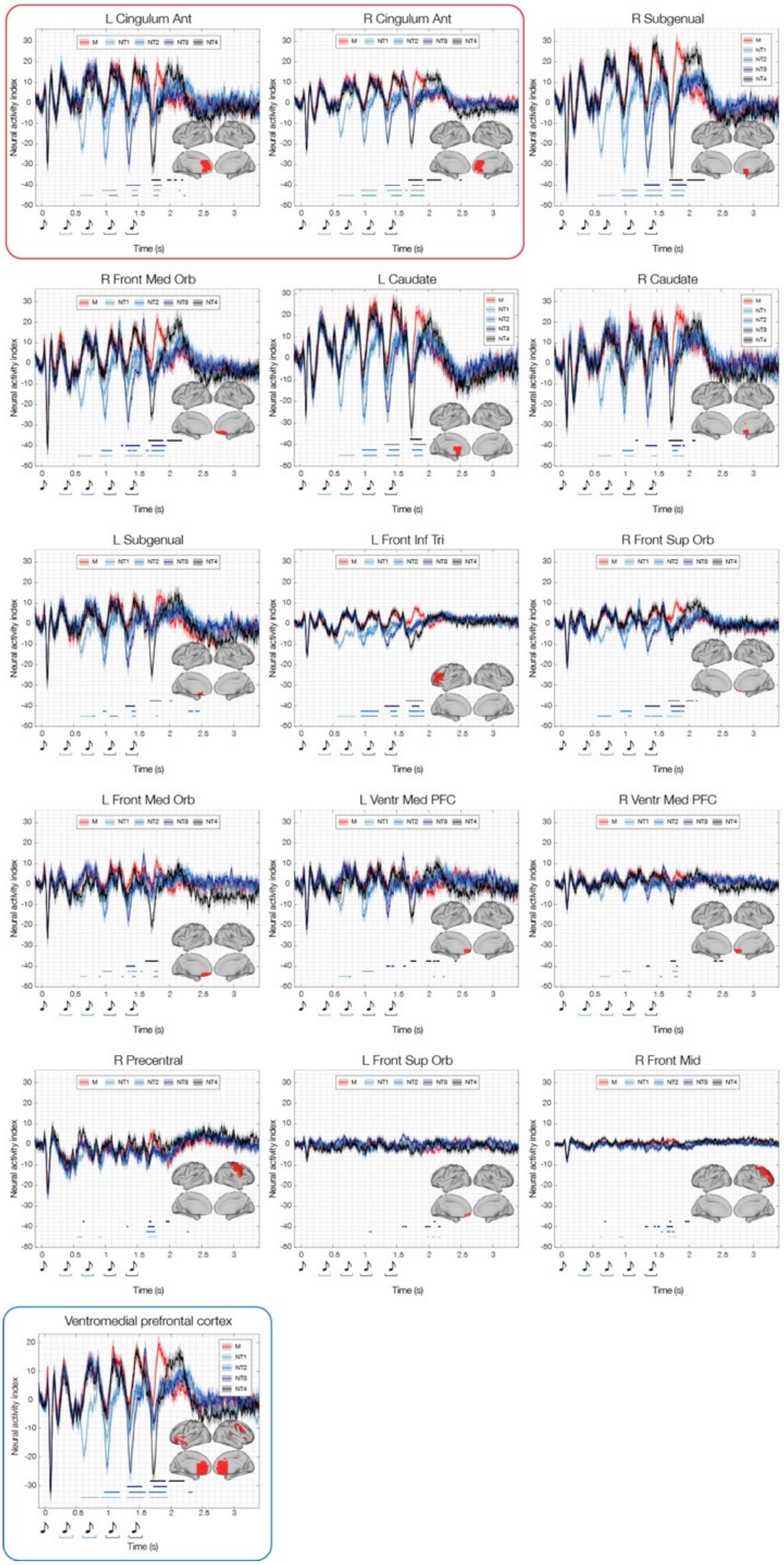

Additional AAL ROIs

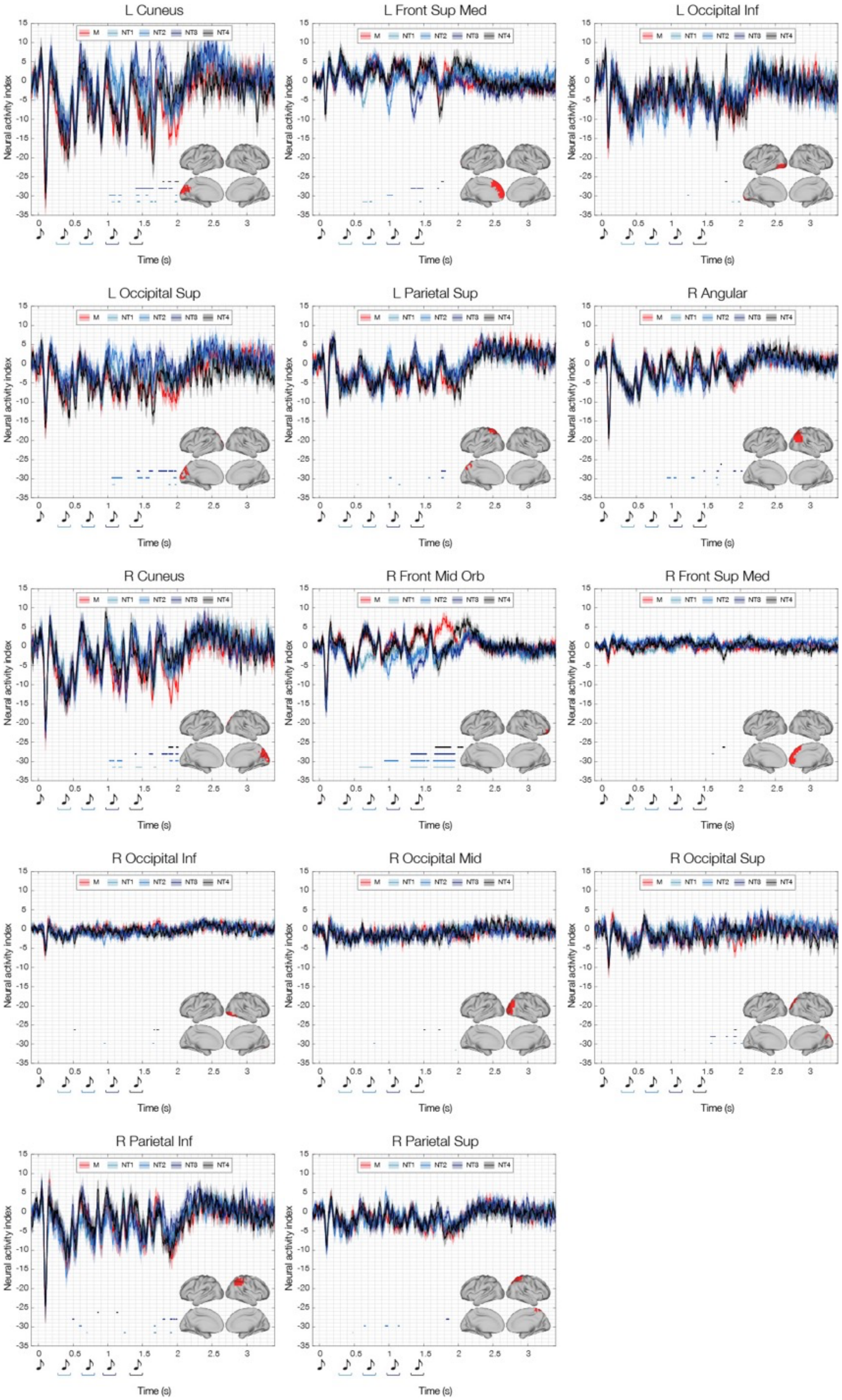

***Figure S7$_{a-g}$*. Source-localised differences in evoked responses across experimental conditions: all automated anatomical labelling (AAL) regions of interest (ROIs)**

*This figure illustrates the source-localised brain activity averaged over participants (n = 83) for each experimental condition (memorised [M], novel T1 [NT1], novel T2 [NT2], novel T3 [NT3], novel T4 [NT4]) within all AAL ROIs. Shaded areas represent standard errors. Musical tone sketches indicate the onset of the sounds forming the sequences. Brain templates depict the spatial extent of the selected ROIs. Blue-black lines highlight significant differences between M and N conditions, obtained by computing two-sided t-tests, independently for each time-point, and correcting for multiple comparisons using one-dimensional Monte-Carlo simulations (MCS; MCS, α = .05, MCS p-value = .001). Different hues of blue or black correspond to specific M versus N condition comparisons. For example, the lightest blue represents M versus NT1, the second lightest blue represents M versus NT2, and so on. The Figure is arranged into seven subfigures to illustrate all non-cerebellar AAL ROIs time series as well as the mapping between the AAL ROIs and the six ROIs of the functional parcellation described in the supplementary material. Here, the AAL ROIs reported in **Figure 4** of the main manuscript are enclosed in a red box, while the ROIs of the functional parcellation are enclosed in a blue box. The seventh subfigure comprises the AAL ROIs which did not have a direct correspondence to the six functional ROIs. For a detailed statistical report on significant differences between experimental conditions in all AAL ROIs, consult **Supplementary Data 6**.*

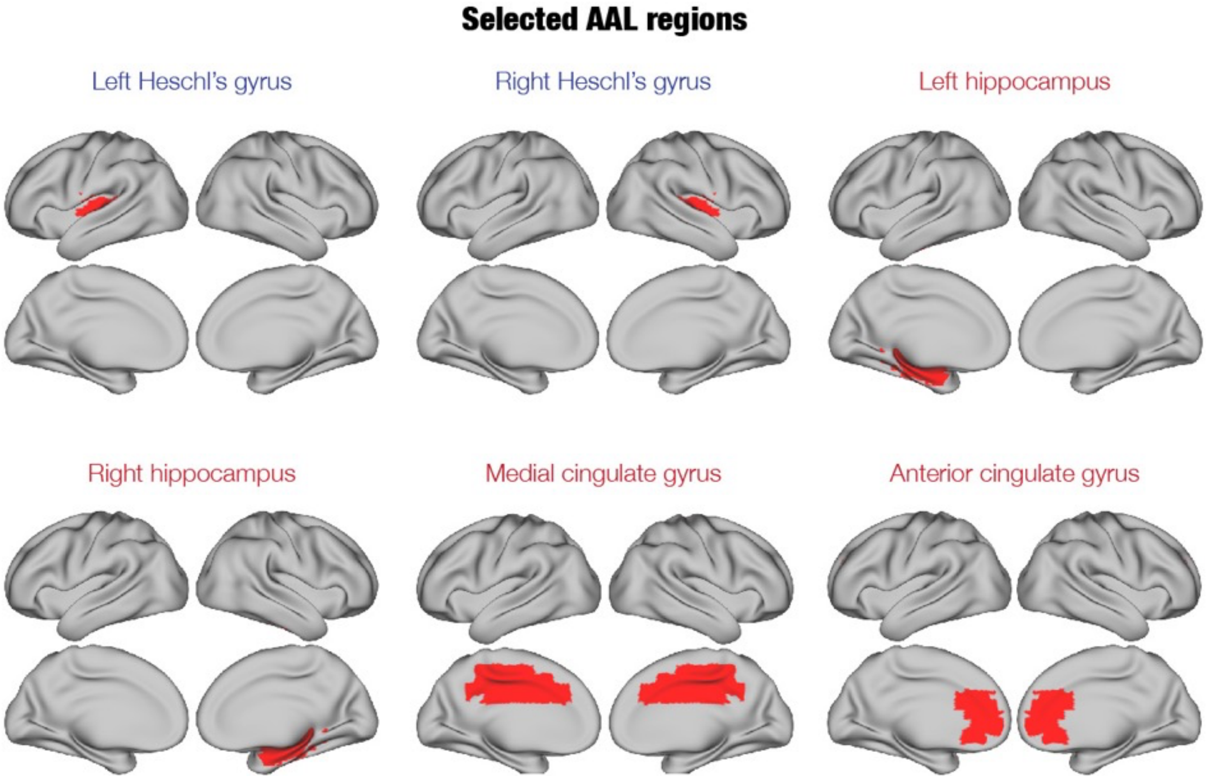

Figure S8. Selected automated anatomical labelling (AAL) regions of interest (ROIs)

Selected array of ROIs which were particularly relevant for this study. Here, we identified the two ROIs that showed the strongest reconstructed activity among auditory regions (i), medial temporal lobe (ii) and cingulate and prefrontal cortices (iii). We selected these broad regions based on the cognitive processes involved in the experimental task used in this study: (i) audition, (ii) memory, and (iii) evaluation and decision-making. As also shown by ***Figure 4****, these ROIs were left and right Heschl's gyrus (LHG, RHG), left and right hippocampus (LHP, RHP), anterior cingulate gyrus (ACC) and medial cingulate gyrus (MC), respectively.*

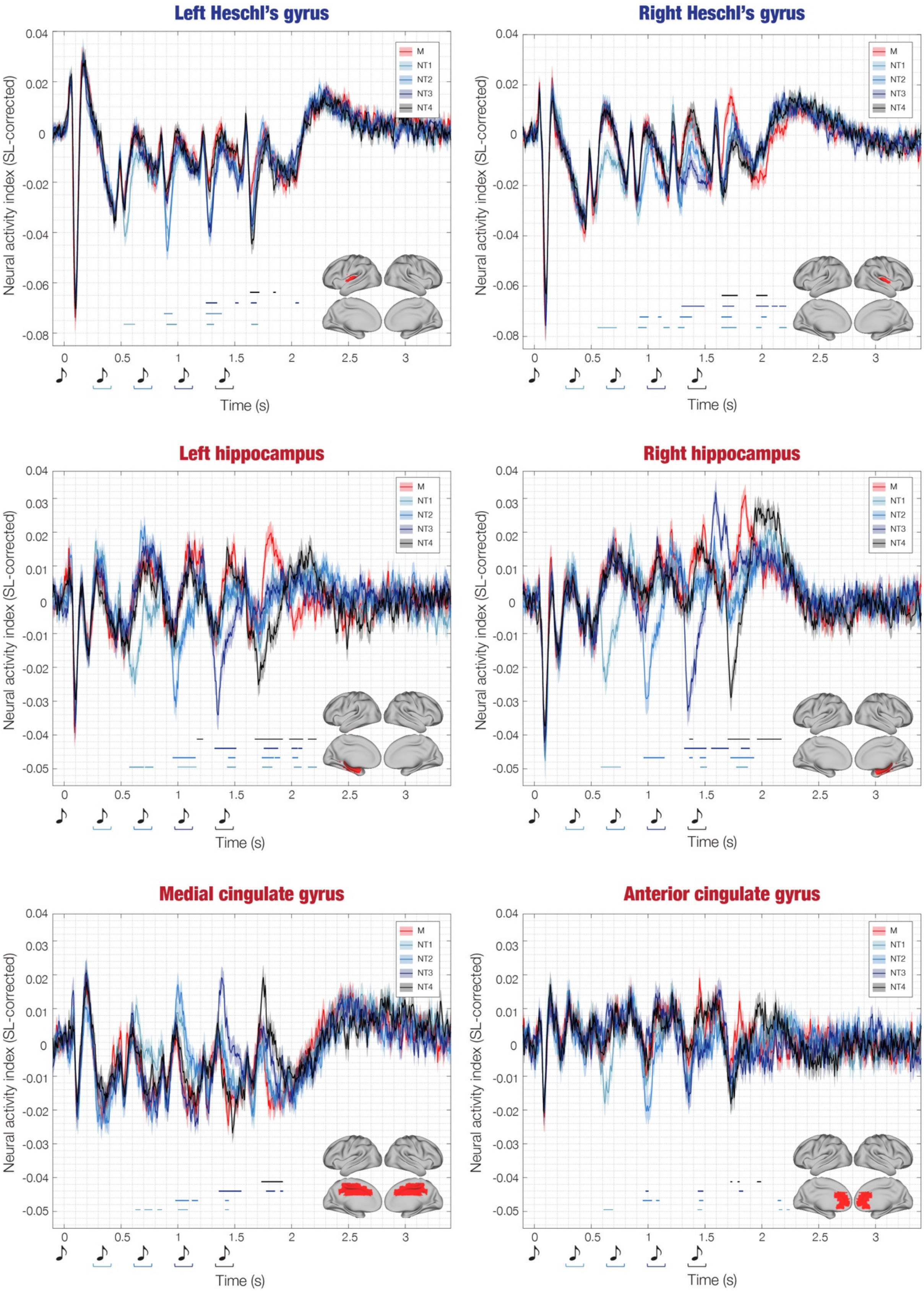

Figure S9. Source-localised differences in evoked responses across experimental conditions – source leakage correction

*Exactly as **Figure 4**, this figure illustrates the source-localised brain activity averaged over participants (n = 83) for each experimental condition (memorised [M], novel T1 [NT1], novel T2 [NT2], novel T3 [NT3], novel T4 [NT4]) within six selected automated anatomical labelling (AAL) regions of interest (ROIs): left Heschl's gyrus (LHG), right Heschl's gyrus (RHG), left hippocampus (LHP), right hippocampus (RHP), anterior cingulate gyrus (ACC), medial cingulate gyrus (MC). Shaded areas represent standard errors. In this case, the time series were corrected for source leakage using a multivariate orthogonalization approach, as proposed by Colclough and colleagues [1]. Musical tone sketches indicate the onset of the sounds forming the sequences. Brain templates depict the spatial extent of the selected ROIs. Blue-black lines highlight significant differences between M and N conditions, obtained by computing two-sided t-tests, independently for each time-point, and correcting for multiple comparisons using one-dimensional Monte-Carlo simulations (MCS; MCS, α = .05, MCS p-value = .001). Different hues of blue or black correspond to specific M versus N condition comparisons. For example, lightest blue represents M versus NT1, the second lightest blue represents M versus NT2, and so on. For a detailed statistical report on significant differences between experimental conditions in all source leakage corrected ROIs time series, consult **Supplementary Data 8***

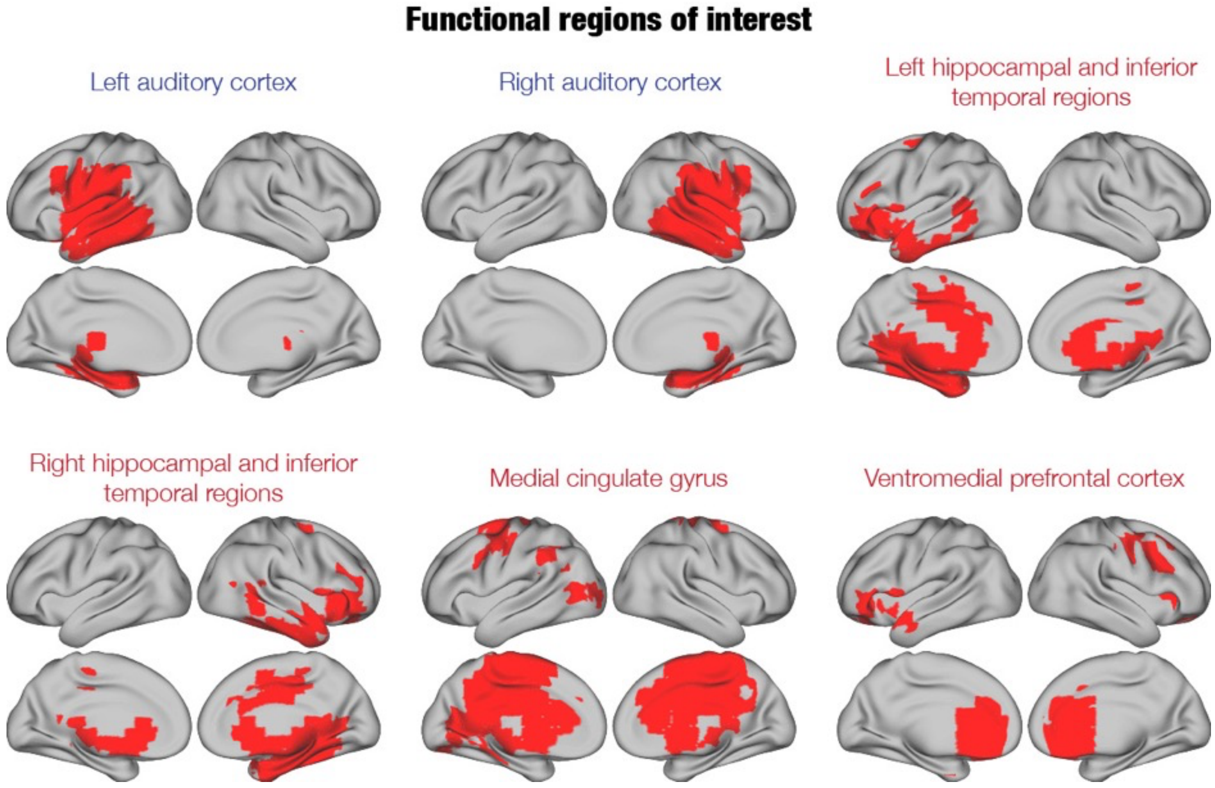

Figure S10. Functional regions of interest (ROIs) derived from the brain activity underlying the task

The main activity during recognition of memorised (M) and novel (N) auditory sequences gave rise to the following six functional ROIs: left (ACL, i) and right auditory cortex (ACR, ii); left (HITL, iii) and right hippocampal regions and inferior temporal cortex (HITR, iv); medial cingulate gyrus (MC, v), and ventromedial prefrontal cortex (VMPFC, vi).

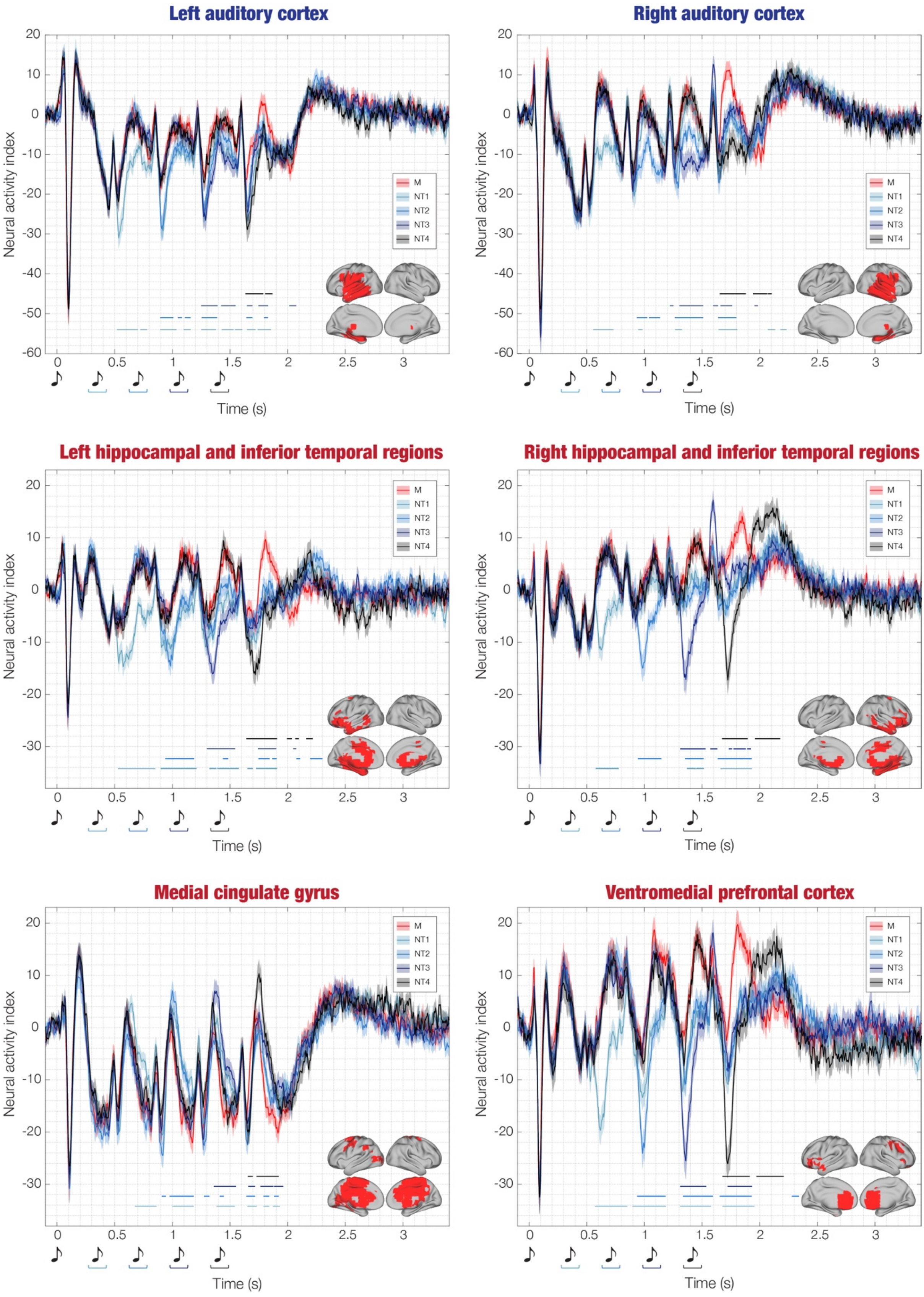
Left auditory cortex
Right auditory cortex
Left hippocampal and inferior temporal regions
Right hippocampal and inferior temporal regions
Medial cingulate gyrus
Ventromedial prefrontal cortex
Neural activity index
Time (s)
M
NT1
NT2
NT3
NT4

Figure S11. Source-localised differences in evoked responses across experimental conditions – functional regions of interest (ROIs)

This figure illustrates the source-localised brain activity averaged over participants (n = 83) for each experimental condition (memorised [M], novel T1 [NT1], novel T2 [NT2], novel T3 [NT3], novel T4 [NT4]) within the six ROIs of the functional parcellation: left Heschl's gyrus (LHG), right Heschl's gyrus (RHG), left hippocampus (LHP), right hippocampus (RHP), anterior cingulate gyrus (ACC), medial cingulate gyrus (MC). Shaded areas represent standard errors. Musical tone sketches indicate the onset of the sounds forming the sequences. Brain templates depict the spatial extent of the selected ROIs. Blue-black lines highlight significant differences between M and N conditions, obtained by computing two-sided t-tests, independently for each time-point, and correcting for multiple comparisons using one-dimensional Monte-Carlo simulations (MCS; MCS, α = .05, MCS p-value = .001). Different hues of blue or black correspond to specific M versus N condition comparisons. For example, the lightest blue represents M versus NT1, the second lightest blue represents M versus NT2, and so on. For a detailed statistical report on significant differences between experimental conditions in the functional ROIs, consult ***Supplementary Data 9****.*

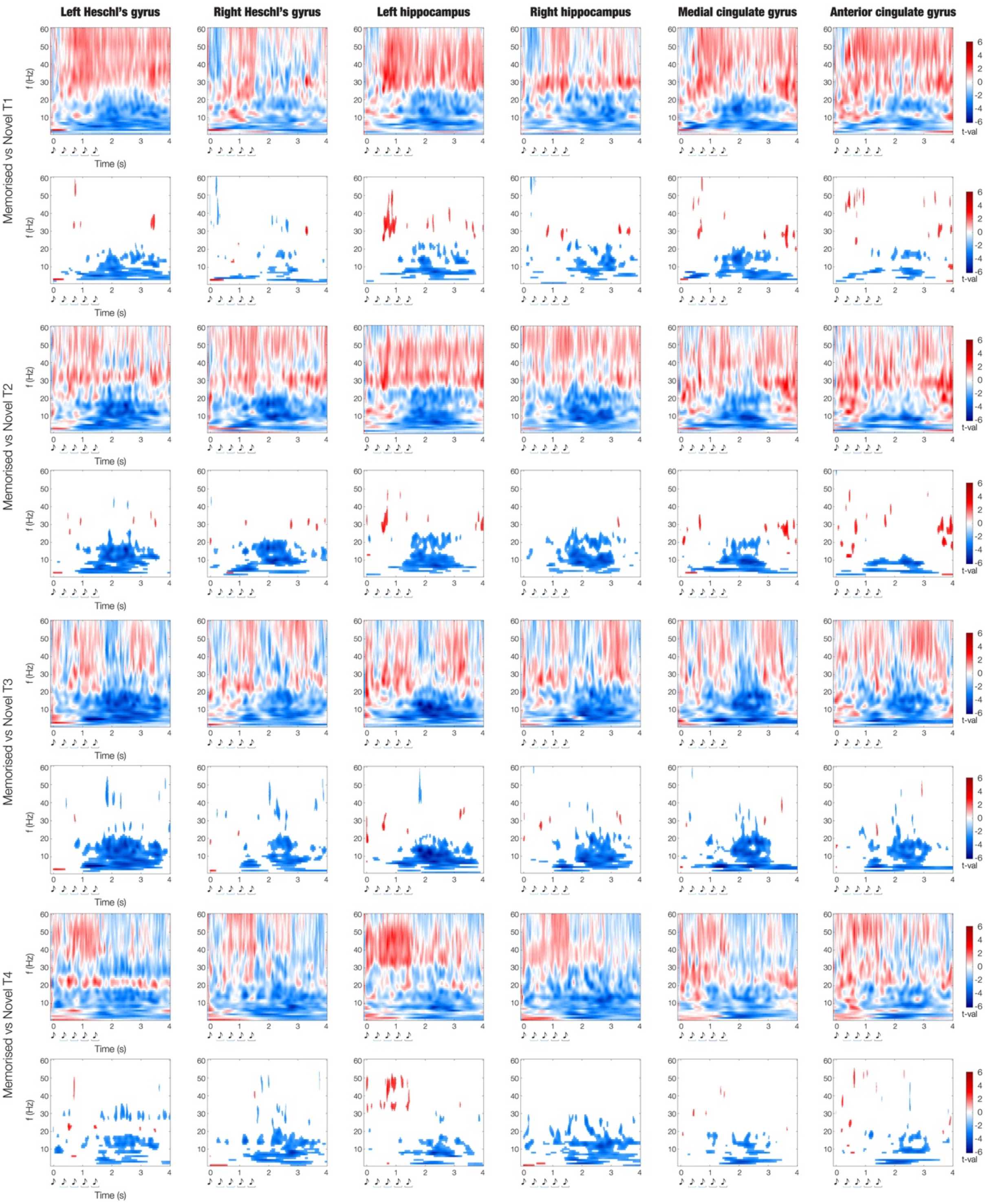

Figure S12. Contrasts for induced responses of selected automated anatomical labelling (AAL) regions of interest (ROIs) for memorised (M) versus all categories of novel (N) sequences

For each of the selected AAL ROIs, two plots are presented for each contrast (i.e. M versus novel T1 [NT1], M versus novel T2 [NT2], M versus novel T3 [NT3], M versus novel T4 [NT4]). The first one represents the contrast between the power spectra of M versus each category of N, one at a time. This contrast is computed using two-sided t-tests (n = 83 participants) and cluster-based Monte-Carlo simulations (MCS; MCS, α = .05, MCS p-value

= .001) correction for multiple comparisons. The second plot only illustrates the significant results emerged from the contrasts. In all cases, the colorbars indicate the t-values obtained by contrasting M versus N.

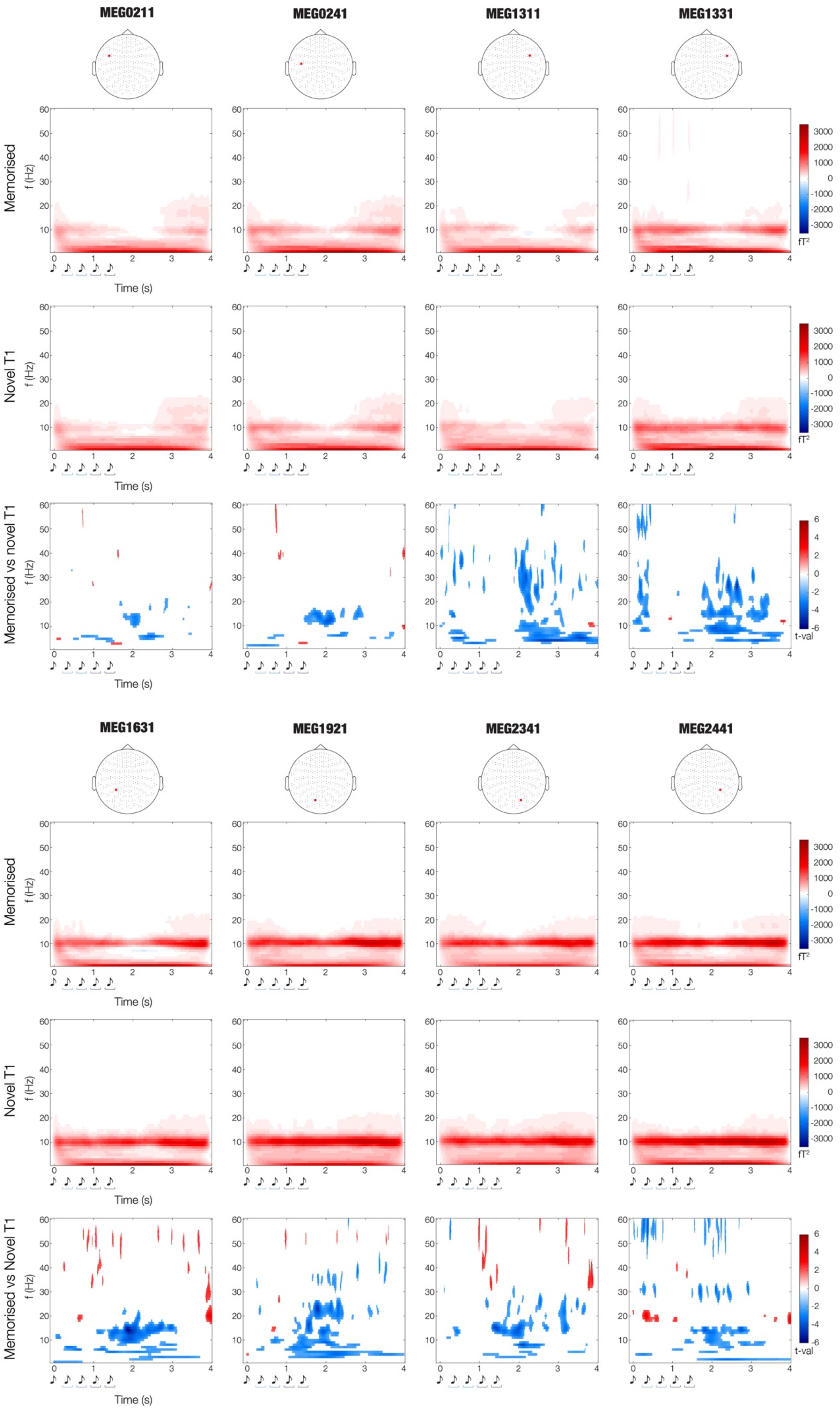
MEG0211
MEG0241
MEG1311
MEG1331
Memorised
Novel T1
Memorised vs novel T1
f (Hz)
Time (s)
fT²
t-val
MEG1631
MEG1921
MEG2341
MEG2441
Memorised
Novel T1
Memorised vs Novel T1
f (Hz)
Time (s)
fT²
t-val

Figure S13. Induced responses of selected magnetoencephalography (MEG) channels during the recognition of memorised (M) and novel T1 (NT1) sequences

For each of the selected MEG channels (0211, 0241, 1311, 1331, 1631, 1921, 2341, 2441), four plots are provided. First, a graphical depiction of the location of the MEG channel is shown. Second, we illustrate the power spectrum computed using complex Morlet wavelet transform for the M and NT1 conditions. Here, the colorbar shows squared femtotesla (fT^2). Third, we depicted the significant results emerged from the contrasts between the power spectra of M versus NT1. This contrast is computed using two-sided t-tests (n = 83 participants) and cluster-based Monte-Carlo simulations (MCS; MCS, α = .05, MCS p-value = .001) correction for multiple comparisons. In this case, the colorbar indicates the t-values obtained by contrasting M versus NT1.

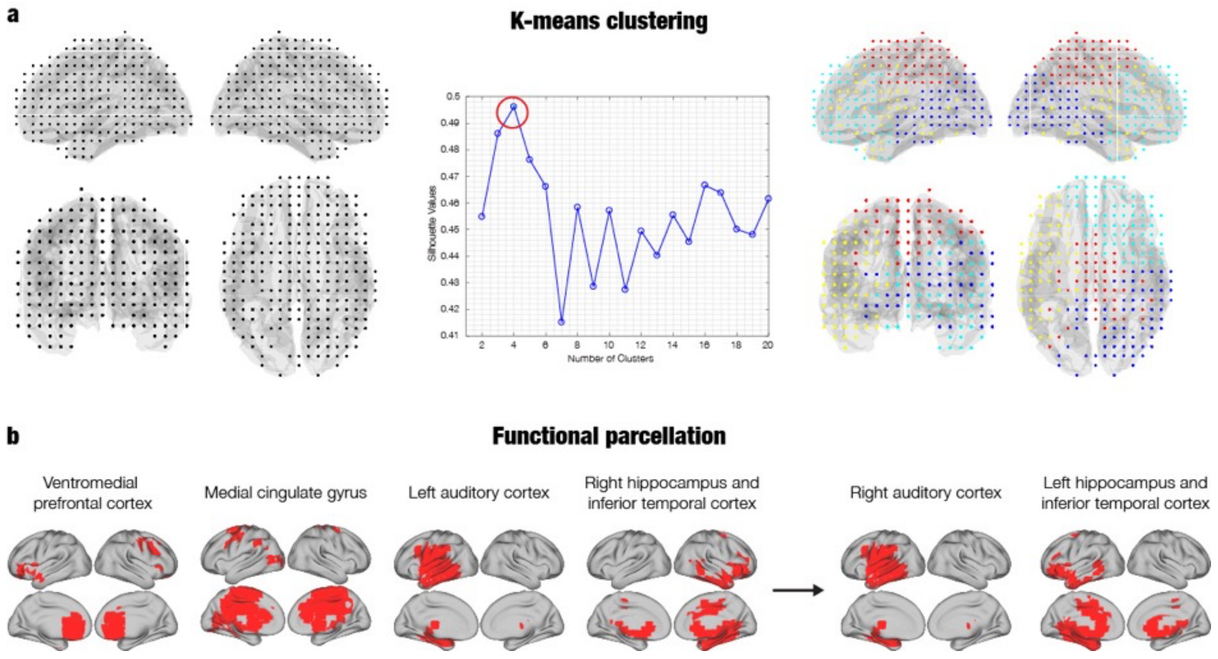

Figure S14. K-means clustering in functional parcellation computation

a - *First, the significant brain voxels emerged from the contrasts depicted in* ***Figure 3*** *were labelled 1 (depicted in the left plot), while the remaining voxels were labelled 0. Then, we computed a series of k-means clusters (clustering solutions from k = 1 to k = 20) on the Montreal Neurological Institute (MNI) spatial coordinates of the brain voxels labelled as '1'. The best clustering solution (k = 4, middle plot) was established using the silhouette coefficient, which is a value (ranging from −1 to +1) showing the similarity of an element with its own cluster (cohesion) when compared to other clusters (separation). As depicted by the right plot, the resulting four clusters roughly corresponded to four broad regions of interest (ROIs): bilateral medial cingulate gyrus (MC), right hippocampal area and inferior temporal cortex (HITR), left auditory cortex (ACL), and bilateral ventromedial prefrontal cortex (VMPFC).* ***b -*** *Following this, as described in detail in the supplementary material, we refined the spatial extent of these four broad ROIs. Finally, we created mirrored ROIs to account for potential brain hemispheric differences (HITL and ACR; there was no need to do that for MC and VMPFC since they were identified already across the two hemispheres).*

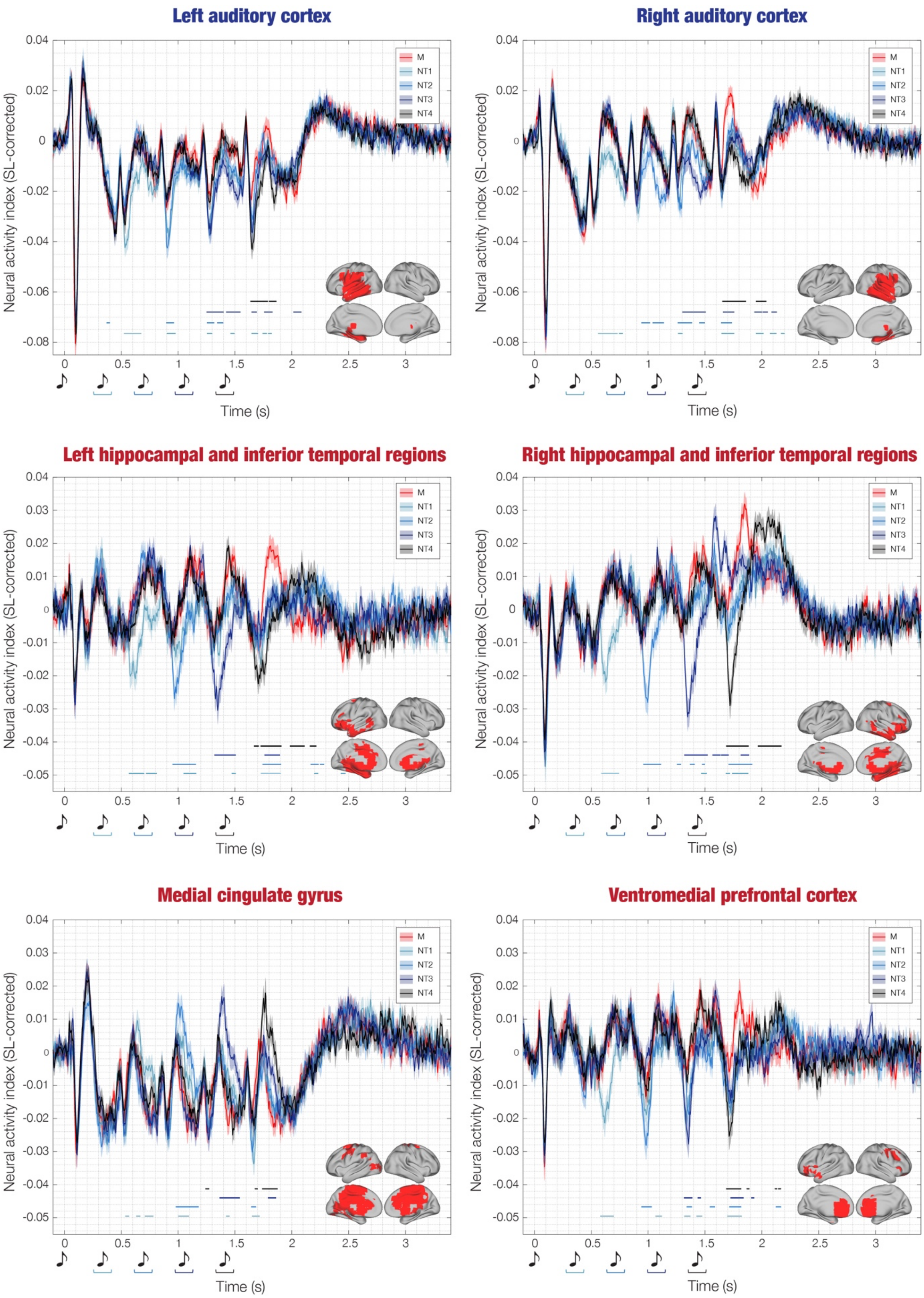

Figure S15. Source-localised differences in evoked responses across experimental conditions – functional regions of interest (ROIs) – source leakage correction

*Exactly as **Figure S11**, this figure illustrates the source-localised brain activity averaged over participants (n = 83) for each experimental condition (memorised [M], novel T1 [NT1], novel T2 [NT2], novel T3 [NT3], novel T4 [NT4]) within the six ROIs of the functional parcellation: left auditory cortex (ACL), right auditory cortex (ACR), left hippocampus and inferior temporal cortex (HITL), right hippocampus and inferior temporal cortex (HITR), medial cingulate (MC), ventromedial prefrontal cortex (VMPFC). Shaded areas represent standard errors. In this case, the time series were corrected for source leakage using a multivariate orthogonalization approach, as proposed by Colclough and colleagues [1]. Musical tone sketches indicate the onset of the sounds forming the sequences. Brain templates depict the spatial extent of the selected ROIs. Blue-black lines highlight significant differences between M and N conditions, obtained by computing two-sided t-tests, independently for each time-point, and correcting for multiple comparisons using one-dimensional Monte-Carlo simulations (MCS; MCS, α = .05, MCS p-value = .001). Different shades of blue or black correspond to specific M versus N condition comparisons. For example, the lightest blue represents M versus NT1, the second lightest blue represents M versus NT2, and so on. For a detailed statistical report on significant differences between experimental conditions in the functional ROIs time series corrected for source leakage, consult **Supplementary Data 13**.*

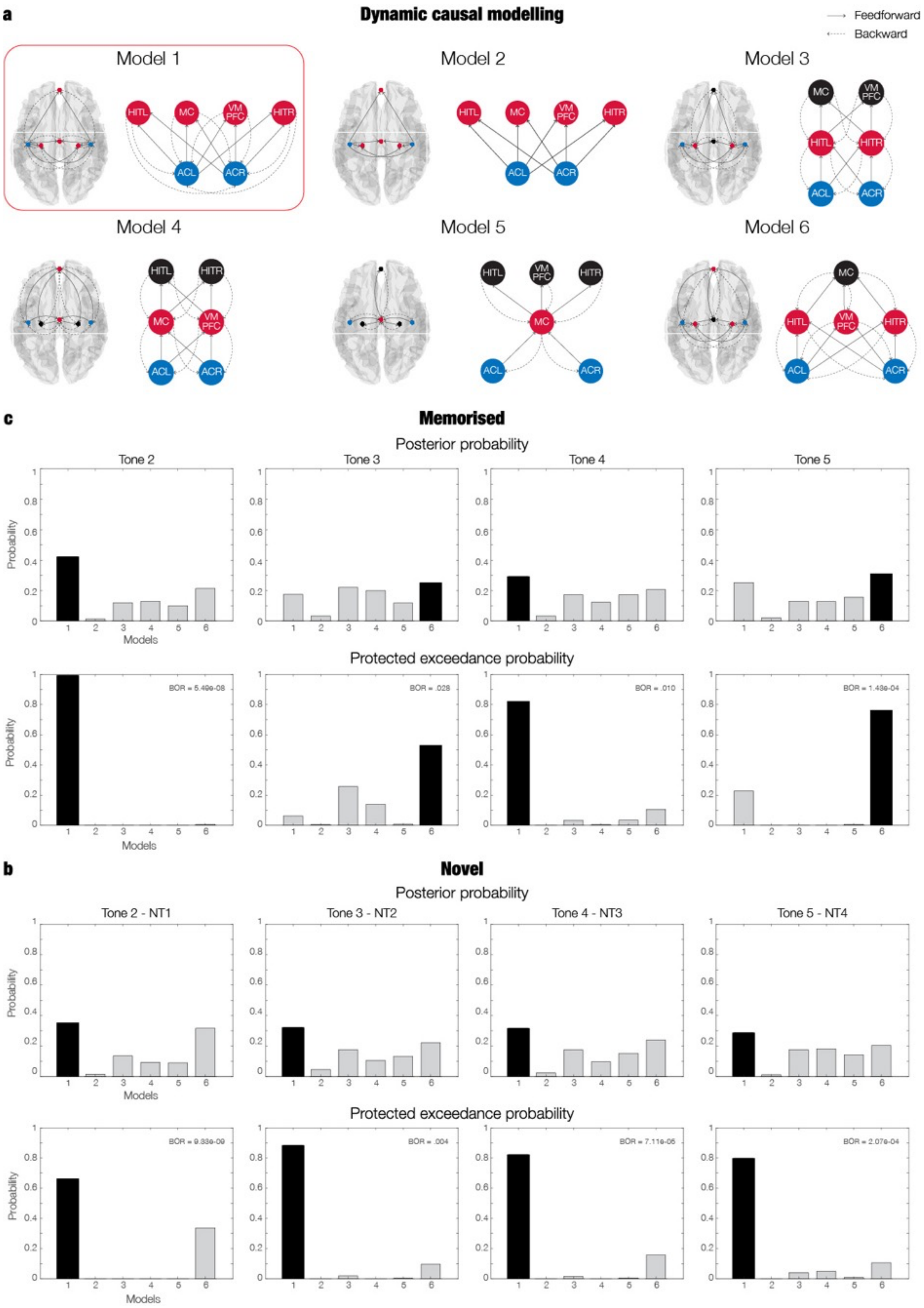
a
Dynamic causal modelling
Feedforward
Backward
Model 1
Model 2
Model 3
Model 4
Model 5
Model 6
HITL
HITR
MC
VM PFC
ACL
ACR
c
Memorised
Posterior probability
Tone 2
Tone 3
Tone 4
Tone 5
Probability
Models
Protected exceedance probability
BOR = 5.49e-08
BOR = .028
BOR = .010
BOR = 1.43e-04
b
Novel
Posterior probability
Tone 2 - NT1
Tone 3 - NT2
Tone 4 - NT3
Tone 5 - NT4
Protected exceedance probability
BOR = 9.33e-09
BOR = .004
BOR = 7.11e-05
BOR = 2.07e-04

Figure S16. Brain hierarchies during recognition of auditory sequences revealed by Dynamic Causal Modelling (DCM) – functional regions of interest (ROIs)

a - *Graphical depiction of the six alternative models employed in DCM analysis. The connections between the six functional ROIs (left auditory cortex [ACL], right auditory cortex [ACR], left hippocampus and inferior temporal cortex [HITL], right hippocampus and inferior temporal cortex [HITR], medial cingulate [MC], ventromedial prefrontal cortex [VMPF]) are depicted within a brain template, and through a graphical representation. Our hypothesised model of brain hierarchies during recognition of memorised and varied musical sequences is enclosed in a red box. Blue, red and black circles illustrate subsequent levels of brain hierarchy.* ***B –*** *Posterior probability, protected exceedance probability and Bayesian omnibus risk (BOR) indicating the model with the highest evidence across the population (inference computed using random-effects Bayesian model selection) for the memorised (M) sequence. We conducted four independent DCM analyses, one for each tone (excluding the first tone, which was shared across all experimental conditions).* ***c –*** *Posterior probability, protected exceedance probability and BOR indicating the model with the highest evidence across the population (n = 83 participants; inference computed using random-effects Bayesian model selection) for the novel (N) conditions. Here, we focused on the first tone which introduced the variation in the sequence (i.e. tone 2 for novel T1 [NT1], tone 3 for novel T2 [NT2], tone 4 for novel T3 [NT3], tone 5 for novel T4 [NT4]).*

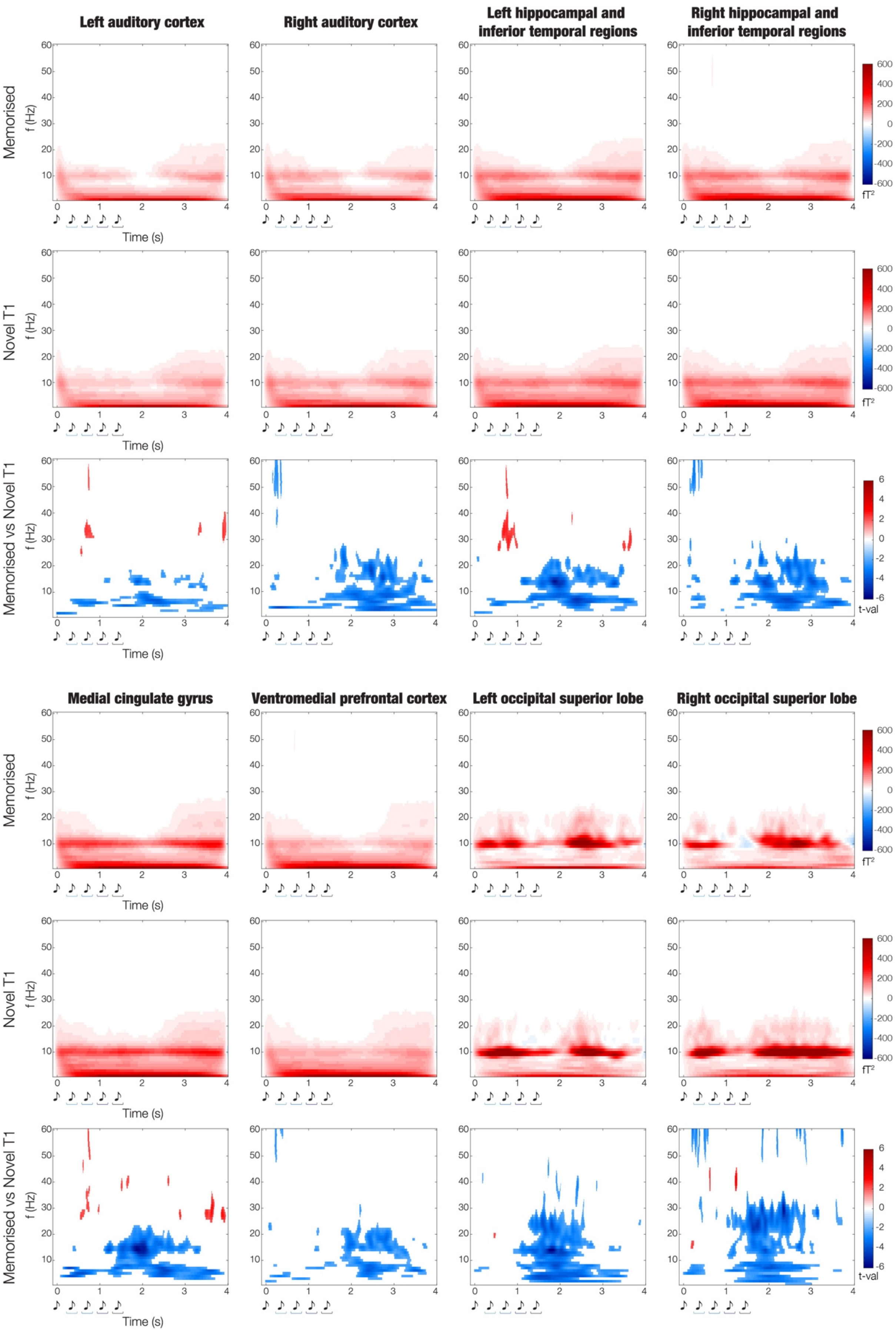
Left auditory cortex
Right auditory cortex
Left hippocampal and inferior temporal regions
Right hippocampal and inferior temporal regions
Medial cingulate gyrus
Ventromedial prefrontal cortex
Left occipital superior lobe
Right occipital superior lobe
Memorised
Novel T1
Memorised vs Novel T1
f (Hz)
Time (s)
fT²
t-val

Figure S17. Induced responses of the functional regions of interest (ROIs) during the recognition of memorised (M) and novel T1 (NT1) sequences

For each of the functional ROIs (plus left and right occipital superior lobe), three plots are provided. First, we illustrated the power spectrum computed using complex Morlet wavelet transform for the M and NT1 conditions. Here, the colorbar shows squared femtotesla (fT^2). Second, we depicted the significant results emerging from the contrasts between the power spectra of M versus NT1 (two-sided t-tests [n = 83 participants] and cluster-based Monte-Carlo simulations [MCS; MCS, α = .05, MCS p-value = .001] correction for multiple comparisons). In this case, the colorbar indicates the t-values obtained by contrasting M versus NT1.

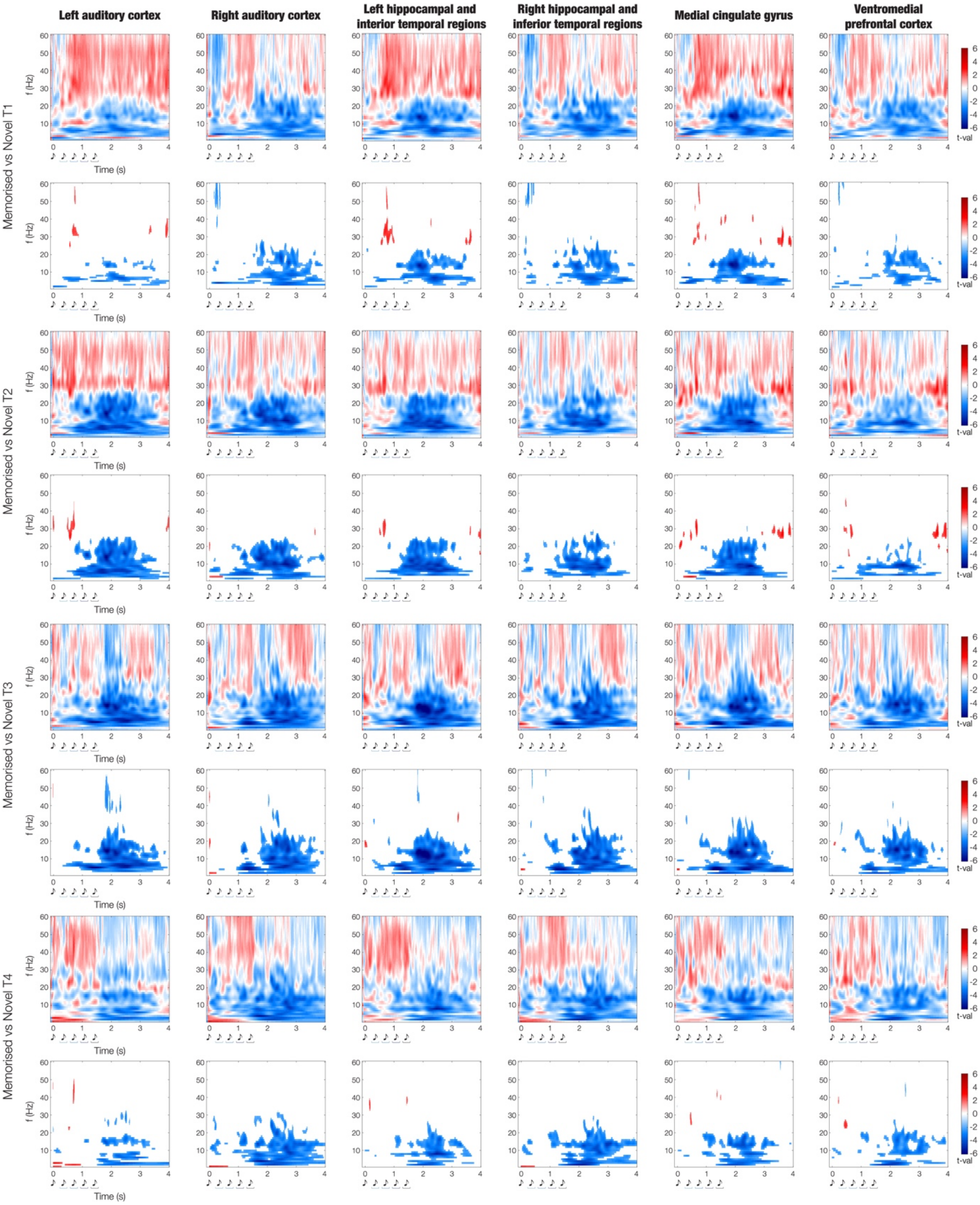

Figure S18. Contrasts for induced responses of functional regions of interest (ROIs) for memorised (M) versus all categories of novel (N) sequences

For each of the functional ROIs, two plots are presented for each contrast (i.e. M versus novel T1 [NT1], M versus novel T2 [NT2], M versus novel T3 [NT3], M versus novel T4 [NT4]). First, we illustrated the contrast between the power spectra of M versus each category of N, one at a time. This contrast is computed using two-sided t-tests (n = 83 participants) and cluster-based Monte-Carlo simulations (MCS; MCS, α = .05, MCS p-value = .001)

correction for multiple comparisons. Second, we depicted the significant results emerged from the contrasts. In all cases, the colorbars indicate the t-values obtained by contrasting M versus N.

Supplementary references

1. Colclough, G.L., Brookes, M.J., Smith, S.M. & Woolrich, M.W. A symmetric multivariate leakage correction for MEG connectomes. *Neuroimage* **117**, 439-448 (2015).
2. Daubechies, I. *Ten lectures on wavelets* (SIAM, 1992).
