# Supplementary material for: Spatiotemporal brain hierarchies of auditory memory recognition and predictive coding
Source: Nat Commun. 2024 May 21;15:4313. doi: 10.1038/s41467-024-48302-4 (PMC11109219; doi:10.1038/s41467-024-48302-4)
Supplement: Supplementary file 3 — Description of Additional Supplementary Files [file 41467_2024_48302_MOESM3_ESM.pdf]

Description of Additional Supplementary Files:

Supplementary Data 1: Pairwise decoding. Binary time series showing the false discovery rate (FDR)-corrected significant time-points (1s) of the decoding time series (i.e., when the algorithm successfully classified memorised [M] versus novel [N]). The first row shows time (in seconds), while the other rows refer to the four contrasts of this study (M versus NT1, M versus NT2, M versus NT3, M versus NT4).

Supplementary Data 2: Temporal generalisation. Cluster-based Monte-Carlo simulations (MCS) on temporal generalisation decoding results computed independently for the four following contrasts: memorised (M) versus novel T1 (NT1), M versus NT2, M versus NT3, M versus NT4. The table shows size, MCS p-value and temporal extent of the cluster (both training and testing sets). Here, the significance was tested using a two-sided signed permutation test against the chance level (50%) for each time-point. Then, we corrected for multiple comparisons using two-dimensional (2D) cluster-based Monte-Carlo simulations (MCS, $\alpha = .05$, MCS p-value = .001).

Supplementary Data 3: Univariate analysis computed on four groups of magnetoencephalography (MEG) channels. Univariate analysis computed on the magnetometers and gradiometers which primarily contributed (one standard deviation plus the mean) to the decoding algorithm. After averaging these channels in four independent groups (see Methods for details), two-sided t-tests were conducted for each time-point and memorised (M) versus novel (NTs) pair (i.e., M versus novel T1 [NT1], M versus novel T2 [NT2], M versus novel T3 [NT3], M versus novel T4 [NT4]), and corrected for multiple comparisons using one-dimensional (1D) cluster-based Monte-Carlo simulations (MCS; $\alpha = .05$, MCS p-value = .001). The significant clusters outputted by the MCS are reported showing cluster size, p-value, temporal extent of the cluster and peak t-value within the cluster.

Supplementary Data 4: Univariate analysis computed independently on all magnetoencephalography (MEG) channels. Univariate analysis computed independently on all magnetometers and non-combined gradiometers MEG channels. Here, for each MEG channel, two-sided t-tests were conducted for each time-point and memorised (M) versus novel (NTs) pair (i.e., M versus novel T1 [NT1], M versus novel T2 [NT2], M versus novel T3 [NT3], M versus novel T4 [NT4]), and corrected for multiple comparisons using one-dimensional (1D) cluster-based Monte-Carlo simulations (MCS; $\alpha = .05$, MCS p-value = .001). The significant clusters outputted by the

MCS are reported showing cluster size, p-value, temporal extent of the cluster and peak t-value within the cluster.

Supplementary Data 5: Contrasts in magnetoencephalography (MEG) 8-mm source space on the neural activity peaks recorded by magnetometers. Within a time-window of ±20 ms around each peak recorded by the magnetometers illustrated in Figure 3a, we averaged the brain activity across all 3559 reconstructed brain voxels for each condition. Then, we computed contrasts between memorised (M) and each category of novel (N) (i.e. M versus novel T1 [NT1], M versus novel T2 [NT2], M versus novel T3 [NT3], M versus novel T4 [NT4]). These contrasts were done by using two-sided t-tests and false discovery rate (FDR) to correct for multiple comparisons. Results are reported with the correspondent automated anatomical labelling (AAL) label of each of the significant voxels, as well as their hemisphere, t-value and Montreal Neurological Institute (MNI) coordinates. Results are depicted in brain templates in Figure 3b.

Supplementary Data 6: Source-localised differences in evoked responses across experimental conditions: all automated anatomical labelling (AAL) regions of interest (ROIs). Univariate analysis computed independently on all AAL ROIs. Here, for each ROI, two-sided t-tests were conducted for each time-point and memorised (M) versus novel (NTs) pair (i.e., M versus novel T1 [NT1], M versus novel T2 [NT2], M versus novel T3 [NT3], M versus novel T4 [NT4]), and corrected for multiple comparisons using one-dimensional (1D) cluster-based Monte-Carlo simulations (MCS; α = .05, MCS p-value = .001). The significant clusters outputted by the MCS are reported showing cluster size, p-value, temporal extent of the cluster and peak t-value within the cluster. These results are illustrated in Figure S $7_{a\text{-}g}$.

Supplementary Data 7: Source-localised differences in evoked responses across experimental conditions: selected anatomical labelling (AAL) regions of interest (ROIs). Univariate analysis computed independently on six selected AAL ROIs: left Heschl's gyrus (LHG), right Heschl's gyrus (RHG), left hippocampus (LHP), right hippocampus (RHP), anterior cingulate gyrus (ACC). Here, for each ROI, two-sided t-tests were conducted for each time-point and memorised (M) versus novel (NTs) pair (i.e., M versus novel T1 [NT1], M versus novel T2 [NT2], M versus novel T3 [NT3], M versus novel T4 [NT4]), and corrected for multiple comparisons using one-dimensional (1D) cluster-based Monte-Carlo simulations (MCS; α = .05, MCS p-value = .001). The significant clusters outputted by the MCS are reported showing cluster size, p-value, temporal extent of the cluster and peak t-value within the cluster. These results are illustrated in Figure 4.

Supplementary Data 8: Source-localised differences in evoked responses across experimental conditions after source leakage correction: selected anatomical labelling (AAL) regions of interest (ROIs). Univariate analysis computed independently on the time series of six selected AAL ROIs (left Heschl's gyrus [LHG], right Heschl's gyrus [RHG], left hippocampus [LHP], right hippocampus [RHP], anterior cingulate gyrus [ACC]) which were corrected for source leakage using the multivariate orthogonalization method proposed by Colclough and colleagues [1]. Here, for each ROI, two-sided t-tests were conducted for each time-point and memorised (M) versus novel (NTs) pair (i.e., M versus novel T1 [NT1], M versus novel T2 [NT2], M versus novel T3 [NT3], M versus novel T4 [NT4]), and corrected for multiple comparisons using one-dimensional (1D) cluster-based Monte-Carlo simulations (MCS; $\alpha = .05$, MCS p-value = .001). The significant clusters outputted by the MCS are reported showing cluster size, p-value, temporal extent of the cluster and peak t-value within the cluster. These results are illustrated in Figures S9.

Supplementary Data 9: Source-localised differences in evoked responses across experimental conditions: functional regions of interest (ROIs). Univariate analysis computed independently on the six ROIs of the functional parcellation: left auditory cortex (ACL), right auditory cortex (ACR), left hippocampus and inferior temporal cortex (HITL), right hippocampus and inferior temporal cortex (HITR), medial cingulate (MC), ventromedial prefrontal cortex (VMPFC). Here, for each ROI, two-sided t-tests were conducted for each time-point and memorised (M) versus novel (NTs) pair (i.e., M versus novel T1 [NT1], M versus novel T2 [NT2], M versus novel T3 [NT3], M versus novel T4 [NT4]), and corrected for multiple comparisons using one-dimensional (1D) cluster-based Monte-Carlo simulations (MCS; $\alpha = .05$, MCS p-value = .001). The significant clusters outputted by the Monte-Carlo simulations (MCS) are reported showing cluster size, p-value, temporal extent of the cluster and peak t-value within the cluster. These results are illustrated in Figure S11. The table also shows the Montreal Neurological Institute (MNI) coordinates of each voxel forming the functional ROIs, as depicted in Figure S10.

Supplementary Data 10: Post-hoc comparisons for prediction error responses in selected automated anatomical labelling (AAL) regions of interest (ROIs). This table presents the results of post-hoc comparisons conducted for each of the one-sided analyses of variance (ANOVAs) computed to assess whether prediction error responses within each of the selected AAL ROIs (left Heschl's gyrus [LHG], right Heschl's gyrus [RHG], left hippocampus [LHP], right hippocampus [RHP], anterior cingulate gyrus [ACC]) to different varied tones were statistically significant. The table reports the results independently for novel T1 (NT1), novel T2 (NT2) and novel T3 (NT3) (i.e. the novel conditions

comprising at least two varied tones). For each comparison (e.g., tone two versus tone three), the table includes information on the lower and upper limits of the comparison, the mean difference between tones, and the p-value. The p-values are Tukey-Kramer corrected for multiple comparisons.

Supplementary Data 11: Time-frequency results of induced responses: selected automated anatomical labelling (AAL) regions of interest (ROIs). Significant clusters of differential power in different frequency bands (1 – 60Hz) computed using complex Morlet wavelet transform. Here, a two-sided t-test for each ROI, frequency and time-point was computed for the following four contrasts: M versus novel T1 [NT1], M versus novel T2 [NT2], M versus novel T3 [NT3], M versus novel T4 [NT4]. The emerging p-values were binarized ($\alpha = .05$) and submitted to two-dimensional (2D) Monte-Carlo simulations to correct for multiple comparisons (MCS p-value = .001). Results are reported independently for the six selected AAL ROIs (left Heschl's gyrus [LHG], right Heschl's gyrus [RHG], left hippocampus [LHP], right hippocampus [RHP], anterior cingulate gyrus [ACC]) and for the four contrasts (M versus NT1, M versus NT2, M versus NT3, M versus NT4). Each entry in the table includes details such as cluster size, p-value, and the temporal and frequency extent of the clusters. Additionally, results for M versus NT1 are also reported for the left and right occipital superior lobe. These results are illustrated in Figures 7 and S12.

Supplementary Data 12: Time-frequency results of induced responses: selected magnetoencephalography (MEG) channels. Significant clusters of differential power in different frequency bands (1 – 60Hz) computed using complex Morlet wavelet transform. Here, a two-sided t-test for each MEG channel, frequency and time-point was computed for the following four contrasts: M versus novel T1 [NT1], M versus novel T2 [NT2], M versus novel T3 [NT3], M versus novel T4 [NT4]. The emerging p-values were binarized ($\alpha = .05$) and submitted to two-dimensional (2D) Monte-Carlo simulations to correct for multiple comparisons (MCS p-value = .001). Results are reported independently for eight fronto-temporal and occipital MEG channels (0211, 0241, 1311, 1331, 1631, 1921, 2341, 2441) and for the four contrasts (M versus NT1, M versus NT2, M versus NT3, M versus NT4). Each entry in the table includes details such as cluster size, p-value, and the temporal and frequency extent of the clusters. These results are illustrated in Figure S13.

Supplementary Data 13: Source-localised differences in evoked responses across experimental conditions after source leakage correction: functional regions of interest (ROIs). Univariate analysis computed independently on the six ROIs of the functional parcellation (left auditory cortex [ACL], right auditory cortex [ACR], left hippocampus and inferior temporal cortex [HITL], right

hippocampus and inferior temporal cortex [HITR], medial cingulate MC], ventromedial prefrontal cortex [VMPFC]) which were corrected for source leakage using the multivariate orthogonalization method proposed by Colclough and colleagues [1]. Here, for each ROI, two-sided t-tests were conducted for each time-point and memorised (M) versus novel (NTs) pair (i.e., M versus novel T1 [NT1], M versus novel T2 [NT2], M versus novel T3 [NT3], M versus novel T4 [NT4]), and corrected for multiple comparisons using one-dimensional (1D) cluster-based Monte-Carlo simulations (MCS; α = .05, MCS p-value = .001). The significant clusters outputted by the MCS are reported showing cluster size, p-value, temporal extent of the cluster and peak t-value within the cluster. These results are illustrated in Figure S15.

Supplementary Data 14: Time-frequency results of induced responses: functional regions of interest (ROIs). Significant clusters of differential power in different frequency bands (1 – 60Hz) computed using complex Morlet wavelet transform. Here, a two-sided t-test for each ROI, frequency and time-point was computed for the following four contrasts: M versus novel T1 [NT1], M versus novel T2 [NT2], M versus novel T3 [NT3], M versus novel T4 [NT4]. The emerging p-values were binarized (α = .05) and submitted to two-dimensional (2D) Monte-Carlo simulations to correct for multiple comparisons (MCS p-value = .001). Results are reported independently for the six functional ROIs (left auditory cortex [ACL], right auditory cortex [ACR], left hippocampus and inferior temporal cortex [HITL], right hippocampus and inferior temporal cortex [HITR], medial cingulate MC], ventromedial prefrontal cortex [VMPFC]) and for the four contrasts (M versus novel NT1, M versus NT2, M versus NT3, M versus NT4). Each entry in the table includes details such as cluster size, p-value, and the temporal and frequency extent of the clusters. These results are illustrated in Figures S17 and S18.
